# The mechanism of ferroptosis and its related diseases

**DOI:** 10.1186/s43556-023-00142-2

**Published:** 2023-10-16

**Authors:** Shijian Feng, Dan Tang, Yichang Wang, Xiang Li, Hui Bao, Chengbing Tang, Xiuju Dong, Xinna Li, Qinxue Yang, Yun Yan, Zhijie Yin, Tiantian Shang, Kaixuan Zheng, Xiaofang Huang, Zuheng Wei, Kunjie Wang, Shiqian Qi

**Affiliations:** 1 Department of Urology and Institute of Urology (Laboratory of Reconstructive Urology), State Key Laboratory of Biotherapy and Cancer Center, West China Hospital, Sichuan University, Chengdu, People’s Republic of China; 2 Chengdu Jinjiang Jiaxiang Foreign Languages High School, Chengdu, People’s Republic of China

**Keywords:** Ferroptosis, Lipid peroxidation, Iron metabolism, Regulatory networks, Therapeutic strategies, Diseases

## Abstract

Ferroptosis, a regulated form of cellular death characterized by the iron-mediated accumulation of lipid peroxides, provides a novel avenue for delving into the intersection of cellular metabolism, oxidative stress, and disease pathology. We have witnessed a mounting fascination with ferroptosis, attributed to its pivotal roles across diverse physiological and pathological conditions including developmental processes, metabolic dynamics, oncogenic pathways, neurodegenerative cascades, and traumatic tissue injuries. By unraveling the intricate underpinnings of the molecular machinery, pivotal contributors, intricate signaling conduits, and regulatory networks governing ferroptosis, researchers aim to bridge the gap between the intricacies of this unique mode of cellular death and its multifaceted implications for health and disease. In light of the rapidly advancing landscape of ferroptosis research, we present a comprehensive review aiming at the extensive implications of ferroptosis in the origins and progress of human diseases. This review concludes with a careful analysis of potential treatment approaches carefully designed to either inhibit or promote ferroptosis. Additionally, we have succinctly summarized the potential therapeutic targets and compounds that hold promise in targeting ferroptosis within various diseases. This pivotal facet underscores the burgeoning possibilities for manipulating ferroptosis as a therapeutic strategy. In summary, this review enriched the insights of both investigators and practitioners, while fostering an elevated comprehension of ferroptosis and its latent translational utilities. By revealing the basic processes and investigating treatment possibilities, this review provides a crucial resource for scientists and medical practitioners, aiding in a deep understanding of ferroptosis and its effects in various disease situations.

## Introduction

**Fig. 1:**
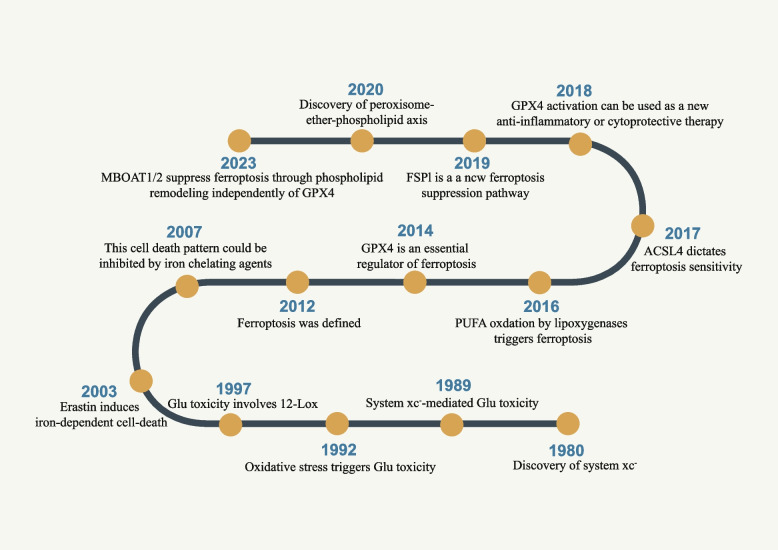
Timeline diagram depicting essential discoveries in the field of ferroptosis research. The exploration of ferroptosis originated from the identification of system xc-, which was initially reported in 1980. Nevertheless, the specific term "ferroptosis" was officially coined and introduced in the scientific community in 2012

Programmed cell death (PCD) plays a critical role in various cellular processes, including embryogenesis, cell growth, cellular immunity, and other biological processes [1–3]. Over the past half-century, several pathways of cell death, such as apoptosis, pyroptosis, and autophagy, have been discovered [4]. In 2003, Erastin and RAS-selective lethal 3 (RSL3) were found to effectively inhibit NRAS-mutant HT-1080, but without the release of mitochondrial cytochrome c, activation of caspase, or fragmentation of chromatin in human foreskin fibroblasts [5]. It was not until 2012 when Dr. Brent R. Stockwell discovered that iron chelation weakens the effect of Erastin in NRAS-mutant HT-1080, leading to the identification of "ferroptosis", an iron-dependent cell death form [6, 7] (Fig. 1).

With the progress of research, ferroptosis was identified as an iron-dependent programmed cell death. Distinct from apoptosis, necrosis, and autophagy, the morphological feature of cells in ferroptosis include mitochondria shrinkage and membrane density increased [7, 8]. The unique process of ferroptosis is the dysregulation of iron metabolism and the accumulation of reactive oxygen species (ROS) [9, 10]. The sufficient concentration oxidation of polyunsaturated fatty acids (PUFAs) and phospholipids, the dysregulation of iron metabolism, and the loss of antioxidant defense system execute the ferroptosis [11] and the mechanism of ferroptosis involves a complicated interplay between multiple cellular pathways, including iron metabolism, lipid metabolism, and antioxidant defense mechanisms [12].

**Fig. 2:**
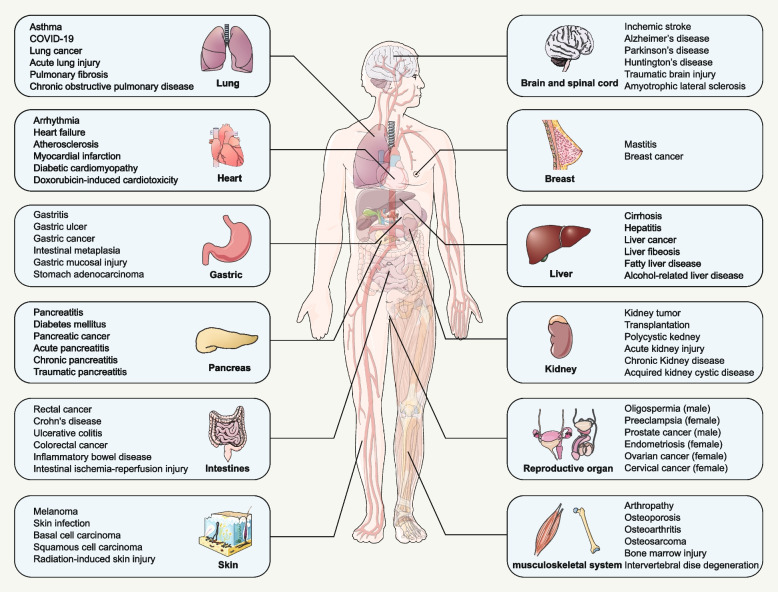
The involvement of ferroptosis in various human diseases. Ferroptosis has played important roles in multiple system diseases, such as lung diseases, nervous system diseases, heart diseases, breast diseases, gastric diseases, liver diseases, pancreatic diseases, kidney diseases, intestines diseases, reproductive diseases, skin diseases, musculoskeletal system diseases and so on

Due to involving various and complicated signaling, ferroptosis plays an important role in the occurrence and development of major chronic diseases and different roles in different disease contexts. A growing body of evidence suggests that the imbalance of ferroptosis affects, development and aging [13, 14], and is closely related to the tumor [8, 15, 16], ischemic diseases [17–22], neurodegenerative disease [23, 24], organ transplantation [25, 26], cardiovascular disease [27–29], autoimmune functions [15, 30], infection [31, 32], iron-overload disease [33], and so on (Fig. 2). Of note, inducing ferroptosis can significantly enhance the sensitivity of chemotherapy drugs to suppress tumor [34, 35], on the other hand, the occurrence of ferroptosis can aggravate the severity of the disease [20, 36]. Although many compounds targeting the key ferroptosis regulators, like glutathione peroxidase 4 (GPX4) and solute carrier family 7 member 11 (SLC7A11, Cystine transporter, also commonly known as xCT), no compounds targeting ferroptosis can be applied to any diseases clinically. Recently, the structure of erastin-bound xCT-4F2hc (4F2 cell-surface antigen heavy chain, SLC3A2, also called CD98) complex had been solved [37], which provides a molecular basis for drugs development targeting on SLC3A2.

In the subsequent sections, our attention converges on the explication of ferroptosis mechanisms, coupled with the accentuation of its pertinent disease-associated targets and bioactive compounds. This assumes pivotal importance, given its potential to create innovative avenues for therapeutic interventions within disorders wherein ferroptosis assumes a key position. This review uncovered the hidden insights about ferroptosis, with the main goal of highlighting its important status as a newly recognized therapeutic target and its deep relevance to various disease states, and aiding researchers in achieving a clearer comprehension of the initiation, progression, and involvement of ferroptosis in various diseases.

## Mechanisms of ferroptosis

**Fig. 3:**
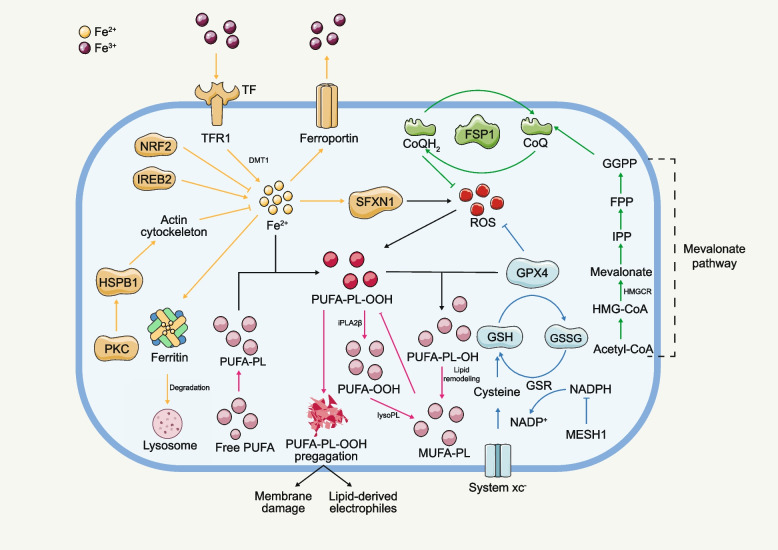
Several intrinsic or cell-autonomous mechanisms profoundly impact cellular susceptibility to ferroptosis. This non-exhaustive compilation encompasses metabolic pathways that intricately regulate iron levels, polyunsaturated fatty acids (PUFA), glutathione peroxidase 4 (GPX4), and ferroptosis suppressor protein 1 (FSP1). Abbreviations: TF: transferrin; TFR1: transferrin receptor 1; NRF2: nuclear factor erythroid 2–related factor 2; IREB2: Iron Responsive Element Binding Protein 2; HSPB1: heat shock protein beta 1; PKC: protein kinase C; Actin cytockeleton: a collection of actin filaments with their accessory and regulatory proteins; Ferritin: a protein that stores iron; SFXN1: siderofexin 1; MUFA: Monounsaturated fatty acids; Acetyl-CoA: acetyl coenzyme; HMG-CoA: 3-hydroxy-3-methylglutaryl coenzyme; IPP: isopentenyl pyrophosphate; FPP: Fertilization promoting peptide; GGPP: geranylgeranyl pyrophosphate; CoQ: coenzyme-Q; CoQH2: reduced coenzyme Q; ROS: Reactive oxygen species; GSH: glutathione; GSSG: glutathione disulfide; NADPH: nicotinamide adenine dinucleotide phosphate; NADP + : Nicotinamide Adenine Dinucleotide Phosphate; MESH1: metazoan SpoT homolog-1

Distinct from conventional cell death forms like apoptosis and necrosis, ferroptosis uniquely hinges on dysregulated iron metabolism and ROS generation [9, 10], featuring an intricate interplay across multiple cellular pathways encompassing iron and lipid metabolism, alongside antioxidant defenses [12]. Dysregulated iron metabolism, characterized by the accumulation of labile iron ions in the cytoplasm, plays a central role in ferroptosis by catalyzing the Fenton reaction, which leads to the production of highly reactive hydroxyl radicals (•OH) from hydrogen peroxide (H_2_O_2_) [38, 39]. These •OH entities interact with cellular membrane PUFAs, kindling lipid peroxidation and ensuing oxidative impairment [40]. Central to ferroptosis, lipid peroxidation arises from PUFAs accruement in cellular membranes [41, 42], predominantly in phospholipids like phosphatidylethanolamine (PE), phosphatidylcholine (PC), and cardiolipin (CL) [43–45]. This accrual engenders lipid peroxidation process, thus destabilizing cellular membranes, leading to cellular damage and ultimately cell death [46]. The cellular antioxidant defense system [47, 48], including enzymes such as superoxide dismutase (SOD) [10, 49], GPX4 [42, 50], catalase [51], alongside non-enzymatic antioxidants such as glutathione (GSH) [50] and vitamin E [52], orchestrates ferroptosis regulation. These constituents synergistically counteract ROS and lipid hydroperoxides, forestalling lipid peroxidation and consequent ferroptosis (Fig. 3). Beyond iron and lipid metabolism, and antioxidant defense mechanisms, several other pathways contribute to ferroptosis modulation. These encompass cellular metabolism [53], the activity of lipid metabolism enzymes [54], and the modulation of cellular redox status [55]. Furthermore, the interplay between ferroptosis and other types of cell death is an active area of research that continues to expand our understanding of the mechanism of ferroptosis [49, 56, 57]. A deeper understanding of the molecular and cellular mechanisms underlying ferroptosis increase the potential to uncover novel therapeutic targets and strategies for the treatment of various diseases associated with dysregulated iron metabolism and oxidative stress.

### The roles of iron metabolism

Dietary iron, predominantly in oxidized ferric (Fe^3+^) form, is assimilated by duodenal and proximal jejunal enterocytes through the divalent metal transporter 1 (DMT1) [58–60]. To be physiologically absorbed, Fe^3+^ must be converted to a ferrous (Fe^2+^) form or bind to co-factors, such as heme [60]. Upon entry into cells, Fe^2+^ associates with transferrin (Tf), which facilitates the translocation of iron into circulation via the iron exporter ferroportin (FPN). Inside the cells, iron is internalized in endosomes via transferrin receptor 1 (TfR1) and then translocated to the cytosol by DMT1, constituting the labile iron pool (LIP)—a crucial source of Fe^2+^ and a key regulator of iron metabolism [61–66]. Mitochondrial iron comes from endosomes through the DMT1 and mitoferrin interaction, or from the LIP, facilitated by DMT1, mitoferrin, and siderofexin (SFXN1) [67–69]. Superfluous iron from the LIP is sequestered in ferritin, of which the lysosomal degradation can replenish the LIP. Cellular iron efflux is mediated by FPN, with hepatocytes and spleen macrophages acting as pivotal iron storage sites [70]. Among the multitude of processes and signaling pathways regulating systemic iron metabolism, the hepcidin-mediated ferroportin internalization and degradation, or the hepcidin-FPN axis, is the paramount mechanism, governing dietary iron absorption and senescent red blood cell recycling [71].

Integral to the basic physiological processes such as oxygen transport, energy synthesis, immune response, DNA replication, and the tricarboxylic acid cycle (TCA), iron's centrality is indisputable [72, 73]. Intriguingly, this iron-sulfur cluster (ISC) -dependent electron transport concurrently augments endogenous ROS generation within mitochondria [72]. While ROS plays an essential role in preserving cellular equilibrium and signaling, the overload of ROS initiates oxidative damage and deleterious outcomes [74, 75]. Concomitantly, iron can also catalyze reactions to induce excessive ROS production via the Fenton reaction, underscoring the delicate balancing of iron metabolism [74, 75]. Therefore, any disturbance in the dynamics of iron import, sequestration, or export can destabilize cellular iron homeostasis, impacting the propensity toward ferroptosis. Substantial evidence suggests that amplified iron import, ferritin degradation (a key iron storage protein), and iron derivative accumulation contribute to ROS production together, thereby igniting the ferroptosis cascade [76, 77].

The orchestration of ROS production via the iron-catalyzed Fenton reaction serves is critical to ferroptosis. Notably, iron-bearing proteins such as Cytochrome P450 enzymes, Nicotinamide adenine dinucleotide phosphate (NADPH) oxidases (NOXs), and subunits of the mitochondrial electron transport chain generate superoxide radicals (O_2_•^−^). Following this, SOD facilitates the conversion of O_2_•^−^ to H_2_O_2_. As a result, heme and containing proteins are oxidized by O_2_•^−^ and H_2_O_2_, leading to the release of reactive Fe^2+^ and the expansion of LIP. This catalysis prompts the Fenton reaction, which, in turn, yields •OH. These •OH then interact with polyunsaturated lipids, causing lipid radicals (L•), lipid peroxidation, and final ferroptosis [78]. Thereafter, L• reacts with additional polyunsaturated lipids, generating lipid hydroperoxide (LOOH) and more L•. Upon interaction with Fe^2+^ and Fe^3+^, LOOH converts into LO• and lipid peroxy radical (LOO•) [79, 80]. Arachidonate-15-lipoxygenase and other iron-containing lipoxygenases (LOXs) catalyze the reaction between O_2_ and polyunsaturated lipids, forming LOOH, with iron integral to the catalytic subunit of LOX. Ferroptosis is typically triggered by iron-dependent LOXs and expanded by the iron-fueled Fenton reaction. Nonetheless, the concentration of iron to initiate ferroptosis remains unclear, necessitating further investigation.

Iron intricately interweaves with the foundational metabolism of glucose, lipids, and amino acids, all of which exhibit pertinent links to ferroptosis [81]. Iron insufficiency is recognized to influence glucose metabolism by affecting glucose utilization, amplifying glucose absorption and transportation via glucose transporter protein type 1(GLUT1). In contrast, iron surplus induces a decrease in insulin sensitivity and the emergence of insulin resistance, culminating in diminished glucose uptake and transport in vitro, but a contrasting impact in vivo [82–87]. Although the explicit role of iron in glucose metabolism remains elusive, these insights imply that glucose is the major metabolic regulator during iron perturbations. Concurrently, iron deficiency impinges on lipid metabolism, which attenuates the rate-limiting enzyme in fatty acid oxidation—Carnitine palmitoyl transferase 1 (CPT-1)—in fetal liver [88]. Moreover, iron surplus initiates the inhibition of hepatic expression of peroxisome proliferator-activated receptor α, while hydroxyl radicals and nitrate anions implicated in the oxidation of PUFAs are also products of the Fenton reaction [89]. Thus, iron deficiency undermines fatty acid oxidation and desaturation while fostering lipogenesis [88–91]. Iron also engages in amino acid transport and synthesis, e.g., 4-hydroxyproline is derived from proline through the iron-dependent dioxygenase prolyl-4-hydroxylase, and cysteine dioxygenase, a key player in cysteine catabolism, is iron enzyme [92, 93]. Though iron plays a critical role in amino acid metabolism, the regulatory details await further exploration [79, 92–95].

**Fig. 4:**
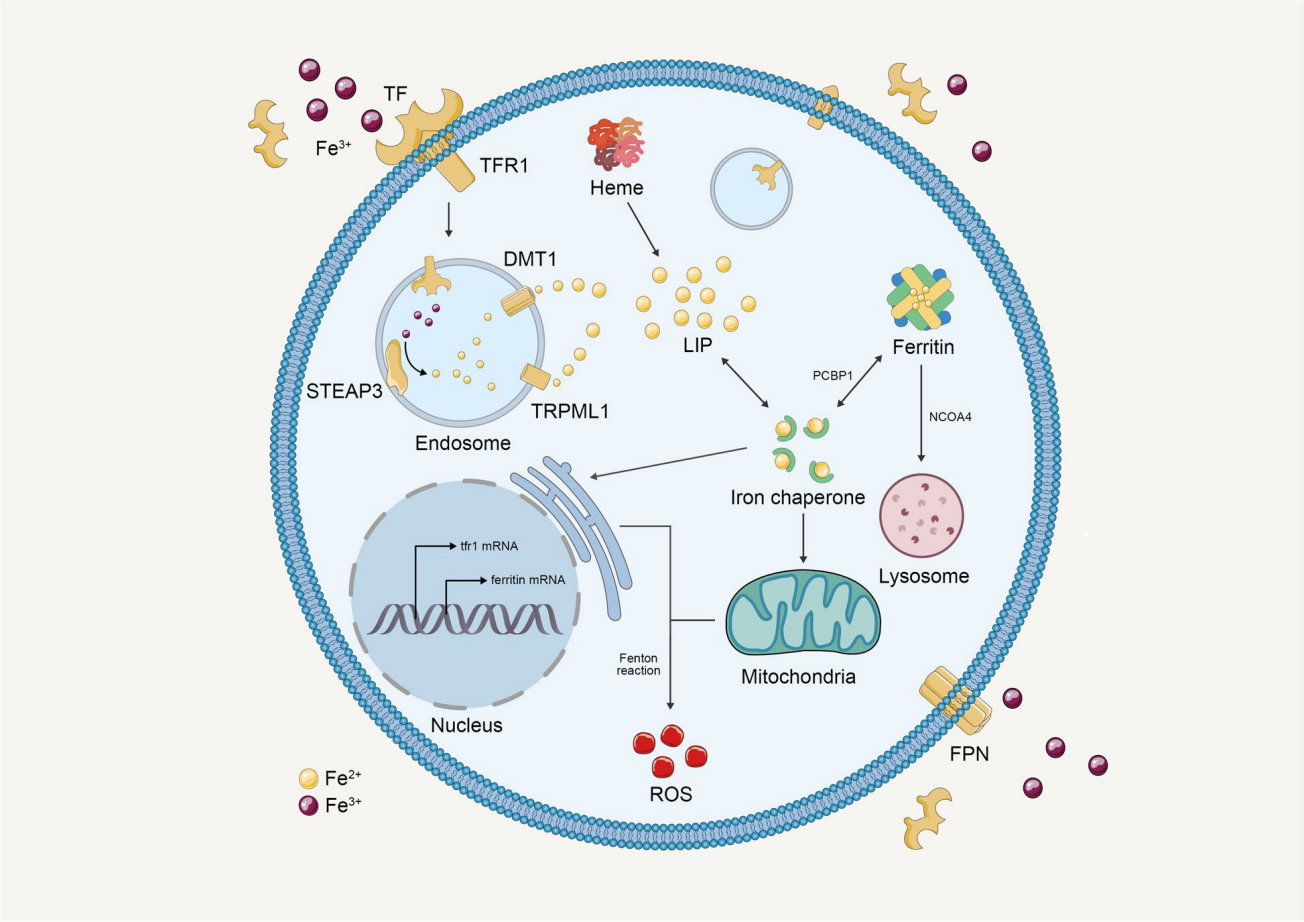
Iron metabolism in ferroptosis. Abbreviations: STEAP3: Six-Transmembrane Epithelial Antigen of Prostate 3; TRPML1: transient receptor potential mucolipin 1; DMT-1: divalent metal transporter 1; NCOA4: Nuclear receptor coactivator 4; FPN: ferroportin

Numerous iron-associated metabolic pathways have been pinpointed to either promote or inhibit ferroptosis. Following iron uptake and the subsequent conversion of Fe^3+^ to Fe^2+^, facilitated by the Six-Transmembrane Epithelial Antigen of Prostate 3 (STEAP3), free Fe^2+^ concentrations escalate, which triggers ferroptosis by propelling the Fenton reaction and lipid peroxidation [74]. Ferritinophagy, the process of ferritin degradation, also yields free Fe^2+^ capable of inducing ferroptosis [96]. Additionally, increased cytoplasmic Fe^2+^ level, caused by ferritinophagy, have been discovered to enhance the expression of SFXN1 on the mitochondrial membrane [96]. SFXN1, reciprocally, expedites the transfer of Fe^2+^ from the cytoplasm to the mitochondria, precipitating mitochondrial ROS production and ferroptosis [97]. Apelin-13, a peptide hormone, is reported to increase the expression of SFXN1 and nuclear receptor coactivator 4 (NCOA4), inducing ferroptosis via ferritinophagy and the shuttling of Fe^2+^ into mitochondria [98, 99] (Fig. 4).

While progress has been made in exploring the mechanisms of iron homeostasis, the functions of iron are not fully understood yet. The roles of iron-mediated ROS production and iron-containing enzymes in this process are still uncertain. The roles of iron homeostasis and proteins following lipid peroxidation in ferroptosis are still elusive, of which, however, the involvement in various diseases like cancer, neurodegenerative diseases, and ischemia–reperfusion injury-related diseases has been noted. Hence, treatments to suppress ferroptosis signals could potentially benefit iron overload diseases. Iron chelating agents are being studied as potential therapies for ferroptosis diseases, though more in vivo studies are needed to clarify the mechanisms and the effect. Future challenges include developing an effective and safe iron chelator. Further studies into the mechanisms of iron-dependent lipid peroxidation are required to identify more treatment targets for diseases associated with ferroptosis, as well as whether iron overload alone can cause ferroptosis in different cells or tissues.

### Lipid peroxidation

Lipid peroxidation, a critical mechanism in ferroptosis, is a procedure in which oxidizing agents, like free radicals, target lipids that possess carbon–carbon double bonds, particularly in PUFAs [100–103]. Lipid peroxidation includes three sequential phases: inception, perpetuation, and cessation [104–106]. Initiating with the inception phase, prooxidants, such as hydroxyl radicals, pluck an electron from allylic hydrogen, yielding a carbon-centric L•. Transiting to the perpetuation phase, this lipid radical swiftly amalgamates with oxygen, thus generating a LOO•. Subsequently, the LOO• detaches a hydrogen atom from a distinct lipid molecule, producing a nascent lipid radical and LOOH, which perpetuates the chain reaction. Ending in the cessation phase, antioxidants, like vitamin E, donate a hydrogen atom to the LOO•, thus producing a corresponding vitamin E radical. This nascent radical then interacts with another LOO•, resulting in the synthesis of non-radical derivatives. It is noteworthy that, once catalyzed, lipid peroxidation induces a cascade of chain reactions until cessation derivatives are generated [104, 107, 108].

The link between lipid peroxidation and ferroptosis arises from the fact that the accumulation of lipid peroxides to lethal levels during the ferroptosis process [43, 50]. Specifically, the oxidation of PUFAs is crucial for the execution of ferroptosis [40, 45, 109]. The process is facilitated by lipoxygenases and iron [44]. Importantly, lipid peroxidation in ferroptosis is delicately regulated by several systems, including the glutathione/GPX4 system and the ferroptosis suppressor protein 1 (FSP1)/CoQ10 system, which neutralize peroxidized lipids and thus inhibit ferroptosis [9]. One of the obvious results of lipid dysregulation is ferroptosis, therefore, investigating lipid peroxidation holds significance in regulating ferroptosis.

**Fig. 5:**
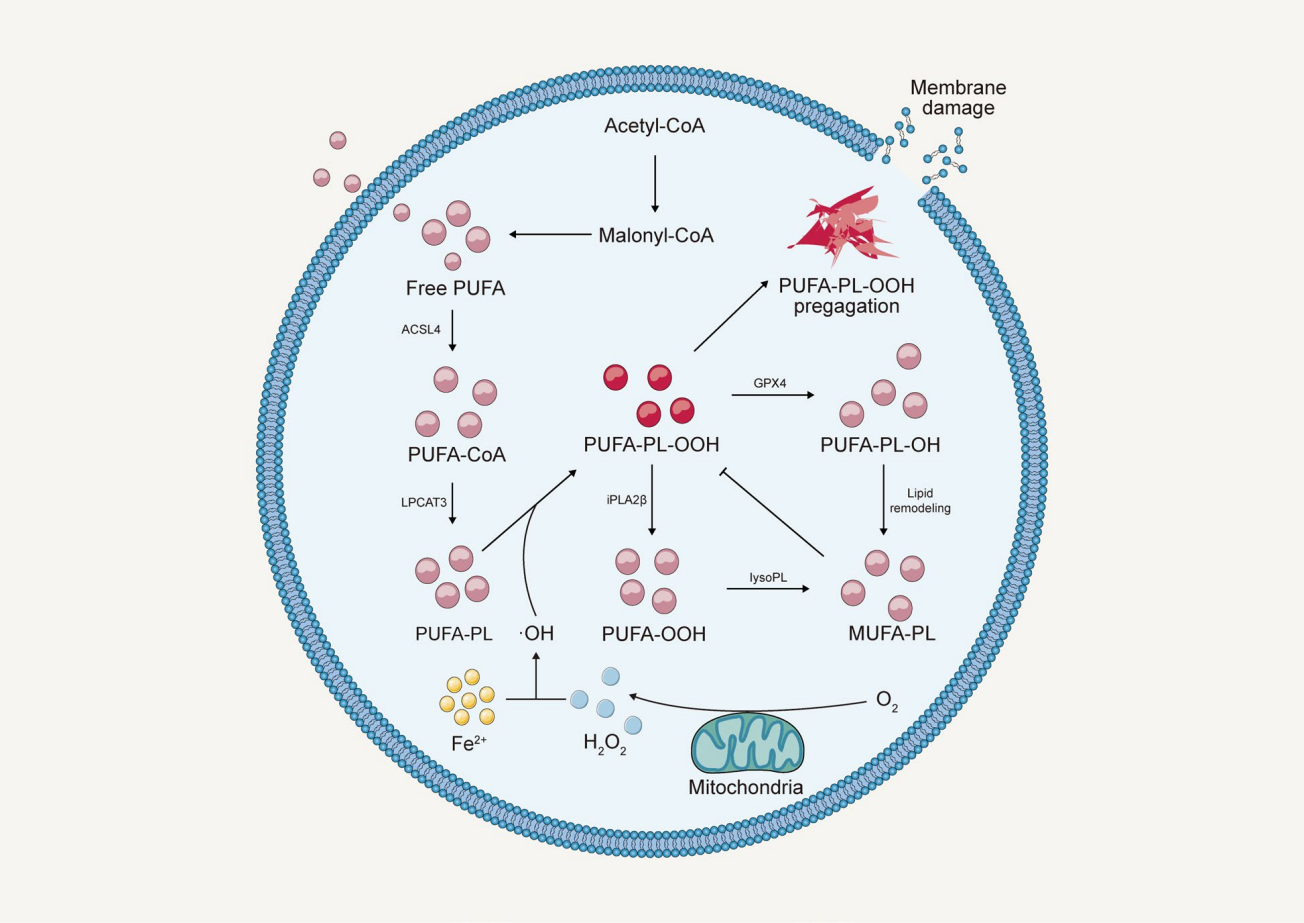
Lipid peroxidation in ferroptosis. Abbreviations: ACSL-4: acyl-CoA synthetase long chain family member 4; LPCAT3: lysophosphatidylcholine acyltransferase 3; LysoPL: lysophospholipase

However, ferroptosis and lipid peroxidation are intertwined yet distinct biological processes. Ferroptosis constitutes a specialized form of regulated cell death marked by the iron-dependent accumulation of lipid peroxides, eventually results in cell membrane deterioration and cell death [9]. In contrast, lipid peroxidation encompasses a broader biochemical phenomenon involving the oxidative breakdown of lipids within cell membranes, often instigated by various oxidative stresses, such as toxins, ultraviolet etc. [107]. While ferroptosis is a specific outcome resulting from disrupted cellular redox balance, lipid peroxidation is a multifaceted process that can occur under diverse conditions, not always leading to cell death. Ferroptosis is thus a subset of the broader lipid peroxidation landscape, characterized by intricate molecular mechanisms and distinctive cellular consequences (Fig. 5).

#### Fatty acids

Fatty acids command a cardinal role in ferroptosis. As indispensable nutrients, they play critical functions in cellular and physiological processes, encompassing energy metabolism and signaling pathways [110]. Four major categories of fatty acids exist: saturated, monounsaturated, polyunsaturated, and trans fats, with PUFAs peroxides reported to exhibit a close association with ferroptosis [111]. PUFAs, containing multiple double bonds (C = C), are predominantly derived from the diet and are pivotal components of cell membranes [112, 113]. They participate in diverse processes, such as inflammation, vascular function, platelet aggregation, synaptic plasticity, cellular growth, immune response, and cellular proliferation [114, 115]. The abundance of double bonds in PUFAs enhances their vulnerability to oxidation, given the susceptibility of the C-H bond in PUFAs to such an oxidative attack [116]. Investigations have underscored that membrane PUFAs are the principal targets of oxidative stress caused by ROS. PUFAs have been found to produce free radicals during their interaction with these ROS, subsequently triggering a cascade that amplifies the extent of damage [78, 104, 117].

Noteworthily, the exogenous introduction of monounsaturated fatty acids (MUFAs), which directly contest with PUFAs, has exhibited an impressive capacity to attenuate erastin-induced ferroptosis [115, 118]. This implies that PUFAs, in contrast to MUFAs, assume a critical role in lipid peroxidation processes and ferroptosis. It has been reported that MUFAs can maintain a state of ferroptosis resistance by curtailing lipid peroxidation in a long-chain acyl-coenzyme A synthases (ACSLs)-dependent manner [9]. Further investigations validated that ACSL3, specifically, is in charge of producing ferroptosis resistance and attenuating saturated fatty acid lipotoxicity [119]. These findings suggest that exogenous MUFAs may change the constitution of the cell membrane by replacing membrane PUFAs and that the replaced PUFAs may be sequestered in cytoplasmic lipid droplets, restraining their pro-ferroptosis activity [120]. Additionally, it has been demonstrated that PUFAs can provoke cancer cell death through escalated ROS production and formation of lipid peroxides [121, 122]. Consequently, the scrupulous regulation of PUFAs and the development of targeted delivery methodologies for PUFAs, as well as techniques to amplify or inhibit ROS and lipid peroxidation production in specific contexts, could provide promising trajectories for therapeutic interventions in various ailments.

#### Ether phospholipids

Ether phospholipids (ePLs), by their unique properties and susceptibility to peroxidation, have been implicated within the matrix of ferroptosis. As a divergent class of phospholipids from the ester phospholipids, ePLs harbor an ether bond at the sn-1 position of the glycerol backbone which is the major difference with an ester bond [123]. Investigations provide a deep understanding of the role of ePLs, particularly plasmalogens, in regulating lipid peroxidation and ferroptosis [123, 124].

ePLs are notably vulnerable to peroxidation by lipoxygenases, potentially catalyzing the accumulation of lipid peroxides and contributing to ferroptosis. This susceptibility hinges on the presence of an ether bond at the sn-1 position of the glycerol backbone of ether phospholipids, which is more vulnerable to ROS assault than the ester bond found in other typical phospholipids [125, 126]. The metabolic reduction of oxidized ether phospholipids, the elimination of lipid peroxides from the membrane, and the suppression of the ether lipid peroxidase have been shown to guard against ferroptosis [127]. The selective vulnerability of certain cells or tissues to ferroptosis is also tied to the levels of ether phospholipids within their membranes.

The proteins related to ePLs are also investigated intensively. Cui et al. reported that sensitization to ferroptosis across various cancer cell lines following TMEM189 deletion. This suggests an unanticipated anti-ferroptosis role for TMEM189, distinguishing it from other ePL biosynthesis genes like glyceronephosphate O-acyltransferase (GNPAT), fatty Acyl-CoA Reductase 1 (FAR1), alkylglycerone phosphate synthase (AGPS), and 1-acylglycerol-3-phosphate o-acyltransferase 3 (AGPAT3) [123, 128]. Cui et al. postulated a mechanistic link where plasmalogens produced by TMEM189 downgrade FAR1 via negative feedback regulation, resulting in the suppression of ferroptosis [123, 128]. However, Zou et al. claimed that TMEM189 deficiency showed no significant link to ferroptosis [124]. The root of this discrepancy seems to lie in the cell lines utilized in the two studies. Further lipidomic analyses in these TMEM189-depleted cell lines will offer clarity on its precise role in the modulation of ferroptosis [129]. Recently, Liang et al. constructed a comprehensive whole-genome CRISPR activation screen and subsequent mechanistic investigation, identified phospholipid-modifying enzymes MBOAT1 and MBOAT2 as potent suppressors of ferroptosis [130]. These enzymes inhibit ferroptosis by reshaping the cellular phospholipid composition, independently of GPX4 or FSP1. Their transcriptional upregulation is governed by sex hormone receptors, estrogen receptor (ER) and androgen receptor (AR). Employing ER or AR antagonists in tandem with ferroptosis induction effectively impedes the growth of ER + breast cancer and AR + prostate cancer, even in cases of resistance to individual hormonal therapies. In summary, the interplay between ether phospholipids and ferroptosis is intricate, involving a delicate balance between susceptibility to lipid peroxidation and protective mechanisms against it. To fully understand the role of ether phospholipids in ferroptosis and their potential as therapeutic targets for diseases characterized by dysregulation of this process, further research is necessary.

#### ACSL4 and LPCAT3

Enzymes catalyzing the incorporation of PUFAs into phospholipids, such as ACSL4 and lysophosphatidylcholine acyltransferase 3 (LPCAT3), are paramount in the orchestration of ferroptosis [45, 131, 132]. ACSL4 plays an fundamental role in the metabolic process of membrane PUFAs, notably arachidonic acid (AA) and adrenic acid (ADA) [133]. This enzyme is critical in the conversion of these fatty acids into their respective CoA thioesters, which subsequently integrate into phospholipids under the guidance of LPCAT3. Both in vivo and in vitro evidence demonstrates that disruption of these enzymatic functions results in heightened resistance to ferroptosis stimuli [45]. Importantly, in the context of hepatocellular carcinoma, ACSL4-dependent mechanisms may have both tumor-promoting and tumor-inhibitory effects [134]. Additionally, evidence derived from both in vivo and in vitro studies corroborate that the ablation of LPCAT3 render a resilience against RSL3-mediated ferroptosis [43, 45, 132]. Therefore, the roles of these enzymes in cellular susceptibility to ferroptosis are pivotal, with implications for cancer progression and therapeutic interventions [135, 136].

#### LOXs and PEBP1

In general, two pathways could regulate lipid peroxidation, non-enzymatic autoxidation and enzyme-mediated reactions [44, 104, 137, 138]. In the presence of free Fe^2+^ and H_2_O_2_, Fe^3+^ is generated and hydroxyl radicals initiate the lipid peroxidation process by abstracting hydrogen from the bis-allylic position of PUFAs [107, 139, 140]. LOXs are non-heme iron-containing dioxygenases that catalyze the stereospecific addition of oxygen onto PUFAs, such as AA and linoleic acids, resulting in lipid peroxidation [141]. Structurally, LOX possesses a unique U-shaped fatty acid binding channel that allows easy access to PUFA substrates [142, 143]. Although several studies have shown that LOX inhibitors/knockout effectively inhibit ferroptosis in various disease models [137, 144], study have also reported that LOX inhibitors/knockout failed to inhibit RSL3-induced ferroptosis in renal carcinoma cells [44]. Further research is still needed to elucidate whether LOXs also participate in GPX4 inhibition during ferroptosis.

The well-known tumor suppressor protein p53 has been implicated in the intricate regulation of ferroptosis. p53 functions include amplifying ferroptosis by impeding the transcription of SLC7A11-an integral constituent of system xc^−^ or by upregulating both spermidine/spermine N1-acetyltransferase 1 (SAT1) and glutaminase 2 [8, 144–146]. Conversely, p53 is also capable of curtailing ferroptosis via the suppression of dipeptidyl-peptidase 4 (DPP4) activity or through the elicitation of Cyclin-dependent kinase inhibitor 1A/p21 (CDKN1A/p21) transcription [147, 148], e.g., p53 can upregulate 15-LOX and thereby increase the sensitivity of cells to induced ferroptosis [144]. p53-mediated ferroptosis in response to TBH is independent of ACSL4, and the specific phospholipids accountable for p53-linked ferroptosis remain unidentified [149].

While LOXs predominantly target free PUFAs for oxidation, phospholipids embedded within the cellular membrane housing PUFAs transpire as the main targets during ferroptosis [44]. Notwithstanding this knowledge, the precise mechanistic pathway employed by LOXs to manipulate membrane phospholipids remains elusive. Preliminary data suggest a robust interaction between 15-LOX and phosphatidylethanolamine-binding protein 1 (PEBP1), a protein proposed to modulate the Raf-1-facilitated mitogen-activated protein kinase (MAPK) signaling cascade [150, 151]. Subsequent investigations hypothesize a stable complex formed between 15-LOX and PEBP1 that can modulate PUFAs, thus invoking ferroptosis [137]. Locostatin, a compound known to escalate oxidized PE concentrations and promote ferroptosis upon RSL3 treatment, is postulated to bolster the formation of the 15-LOX/PEBP1 complex [137]. Various disease models also revealed the accumulation of 15-LOX/PEBP1 complex resulted in elevated oxidized PEs and ferroptosis [137]. Further validation of PEBP1’s integral role in orchestrating ferroptosis arises from the observation that selective ferroptosis inhibitors-ferrostatin-1 (Fer-1), liproxstatin-1, and α-tocopherol-also engage with the 15-LOX2/PEBP1 complex [7, 152]. Whereas corroborating evidence emphasizes PEBP1's fundamental role in producing oxidized PEs, no discernible effects on free ETE (eicosatetraenoic acid) have been reported. Intriguingly, Fer-1 selectively hinders the formation of 15-hydroperoxy (Hp)-arachidonoyl-phosphatidylethanolamine (15-HpETE-PE) but not 15-HpETE, implying that Fer-1 specifically targets the 15-LOX2/PEBP1 complex, leaving free 15-LOX2 unimpeded [153]. These investigations corroborate that the collaboration between LOXs and PEBP1 is crucial in governing lipid peroxidation and the progression of ferroptosis.

#### Other oxygenases

Other oxygenases, such as NOXs and cytochrome p450 oxidoreductase (POR), are also involved in ferroptosis. While NOXs induce superoxide radicals, the extent of their requirement for ferroptosis remains contested [147, 154–157]. POR, identified as a ferroptosis contributor, facilitates electron transfer from NADPH to cytochrome p450, possibly promoting lipid peroxidation. Notably, POR's ubiquitous presence in various cancer cell lines suggests its potential significance in lipid peroxidation and ferroptosis [158, 159]. Further, an ER-resident oxidoreductase, NADH-cytochrome b5 reductase 1 (CYB5R1), and POR have been implicated in lipid peroxidation through H_2_O_2_ production and iron-dependent Fenton reaction [160]. Despite the common belief that LOXs primarily induce lipid peroxidation, their expression is limited in certain cancer cell lines. Intriguingly, POR is expressed in most cancer cells, suggesting an underestimation of POR's role in ferroptosis [159]. A comprehensive understanding of each enzyme's contribution to ferroptosis could pave the way for developing targeted therapeutic agents for related diseases.

### Role of GPX4

Glutathione is a small molecule found in most cells. It is made up of three amino acids: glutamate, cysteine, and glycine. Glutathione is one of the most important antioxidants in cells, as it is responsible for neutralizing a variety of harmful substances [161, 162]. Glutathione exists in reduced GSH and oxidized (GSSG) states [163]. In the reduced state, glutathione can donate a reducing equivalent to unstable molecules like ROS. Once the electron is donated, glutathione becomes oxidized and is turned into GSSG. The ratio of GSH to GSSG within cells is usually used as a measure of cellular oxidative stress [164]. Glutathione serves as a cofactor for the enzyme GPX4, which helps to reduce lipid peroxides and prevent lipid peroxidation [50]. When glutathione is depleted, GPX4 cannot function effectively, leading to an accumulation of lipid peroxides and increased susceptibility to ferroptosis.

System xc^−^/GSH/GPX4 axis is the main mechanism responsible for the catalyzation of phospholipid hydroperopxides [7, 165, 166]. The key component of the xc^−^/GSH/GPX4 axis is system xc^−^, which is a highly selective uptake system for cystine (oxidized cysteine) and cystathionine [167–169]. System xc^−^ exchanges cysteine and glutamate in and out of the cell at a 1:1 ratio [7]. The xCT light chain, which is the substrate-related subunit of system xc^−^, is subject to complicated transcriptional control. Under oxidative stress and cysteine deprivation conditions, xCT is upregulated by apoptosis-inducing factor-4 (ATF4) [170]. It has also been reported that p53 can inhibit xCT expression and increase sensitivity to ferroptosis [8, 171].

Once cystine was taken up by the cell, it is converted to cysteine by GSH and/or thioredoxin reductase 1, which is then used for GSH synthesis [172]. Besides, other mechanisms, such as the transsulfuration pathway and the neutral amino acid transporter, also contribute to cysteine production [173, 174]. Cysteine plays a significant role by contributing the essential redox-active thiol group central to its multifaceted functions. Within cells where GSH is produced, intracellular cysteine concentrations are relatively modest, thereby typically governing GSH synthesis due to the confined availability of cysteine. During instances of heightened demand for GSH synthesis, there is an intensified cellular uptake of cysteine from the more abundant extracellular environment. Interestingly, the predominant extracellular form of cysteine is cystine, characterized by its oxidized state. Subsequent to cellular entry, cystine can undergo reduction to cysteine by cystine reductase, thereafter being channeled towards GSH or protein synthesis. The distinctive recognition of these compounds by specific transporters plays a pivotal role, as the relative concentrations of cysteine and cystine in the plasma modulate the ability of cells to import either substance, contingent upon the unique profiles of transporter expression [175, 176].

**Fig. 6:**
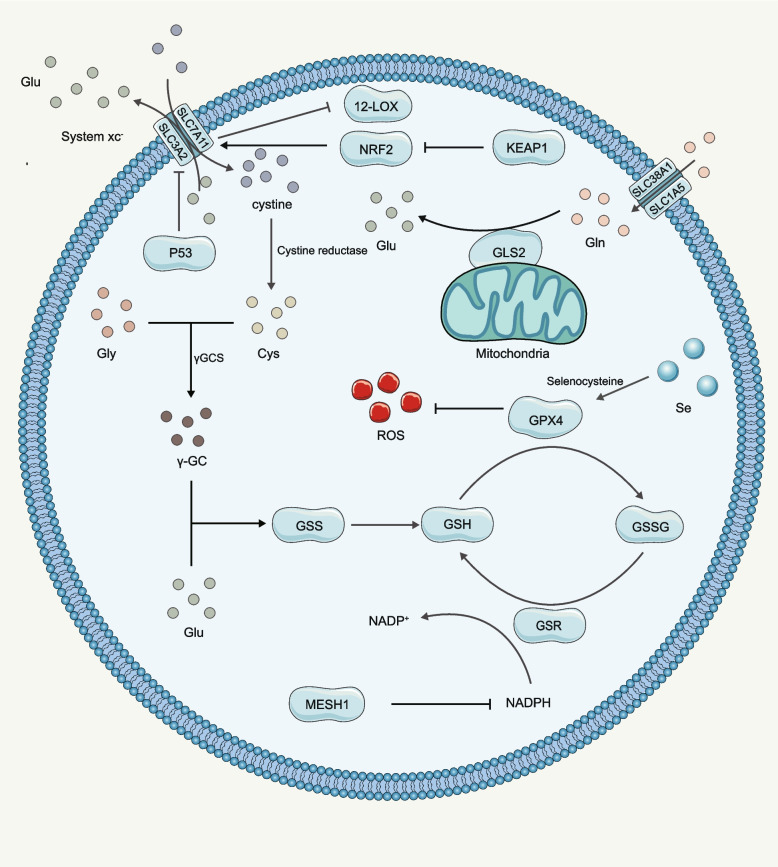
The role of GPX4 in ferroptosis. Abbreviations: Glu: glutamic acid; Gln: Glutamine; Cys: cysteine; Gly: Glycine; P53: a tumor suppressor protein; KEAP1: Kelch-like ECH-associated protein; 12-LOX: 12-lipoxygenase; GLS2: glutaminase 2; γ-GC: γ-glutamylcysteine; GSS: glutathione synthetase; GSR: glutathione reductase

GPX4 takes part in several physiological processes and is considered as the main inhibitory gene of ferroptosis [177]. GPX4 catalyzes lipid peroxides and is crucial for preventing the accumulation of lipid peroxides and subsequent ferroptosis [178]. The GPX4 pathway regulates ferroptosis in several ways: 1) Reduction of lipid peroxides: GPX4 converts lipid peroxides into their corresponding alcohols, which are less toxic and less likely to cause ferroptosis. Inhibition of GPX4 activity leads to the accumulation of lipid peroxides, which triggers ferroptosis. 2) Maintenance of membrane integrity: The cell membrane is particularly susceptible to lipid peroxidation, which can lead to membrane damage and subsequent ferroptosis. GPX4 helps to maintain membrane integrity by reducing lipid peroxides in the cell membrane. 3) Regulation of iron metabolism: Iron is a key mediator of ferroptosis, as it catalyzes lipid peroxidation through the Fenton reaction [178]. GPX4 can also regulate iron metabolism by binding to iron ions and preventing their participation in the Fenton reaction. Overall, the GPX4 pathway plays a crucial role in regulating ferroptosis by reducing lipid peroxides, maintaining membrane integrity, and regulating iron metabolism (Fig. 6).

### Role of FSP1

Studies have indicated that the sensitivity of different cell lines to inhibitors of GPX4 varies significantly, suggesting the existence of unexplored downregulatory mechanisms of ferroptosis beyond GPX4 [179]. Using synthetic lethal CRISPR-Cas9 screening, researchers have identified FSP1 as another key factor in ferroptosis resistance [180, 181]. Initially referred to as AIF-like mitochondrion-associated inducer of death (AMID) or Apoptosis-inducing factor mitochondria-associated 2 (AIFM2, also known as FSP1), FSP1 was the first gene named for ferroptosis [182]. However, unlike AIF, FSP1 is predominantly found in the cytosol, with a potential affinity towards the mitochondrial outer membrane, although it lacks a long N-terminal mitochondrial targeting sequence as seen in AIF [183].

**Fig. 7:**
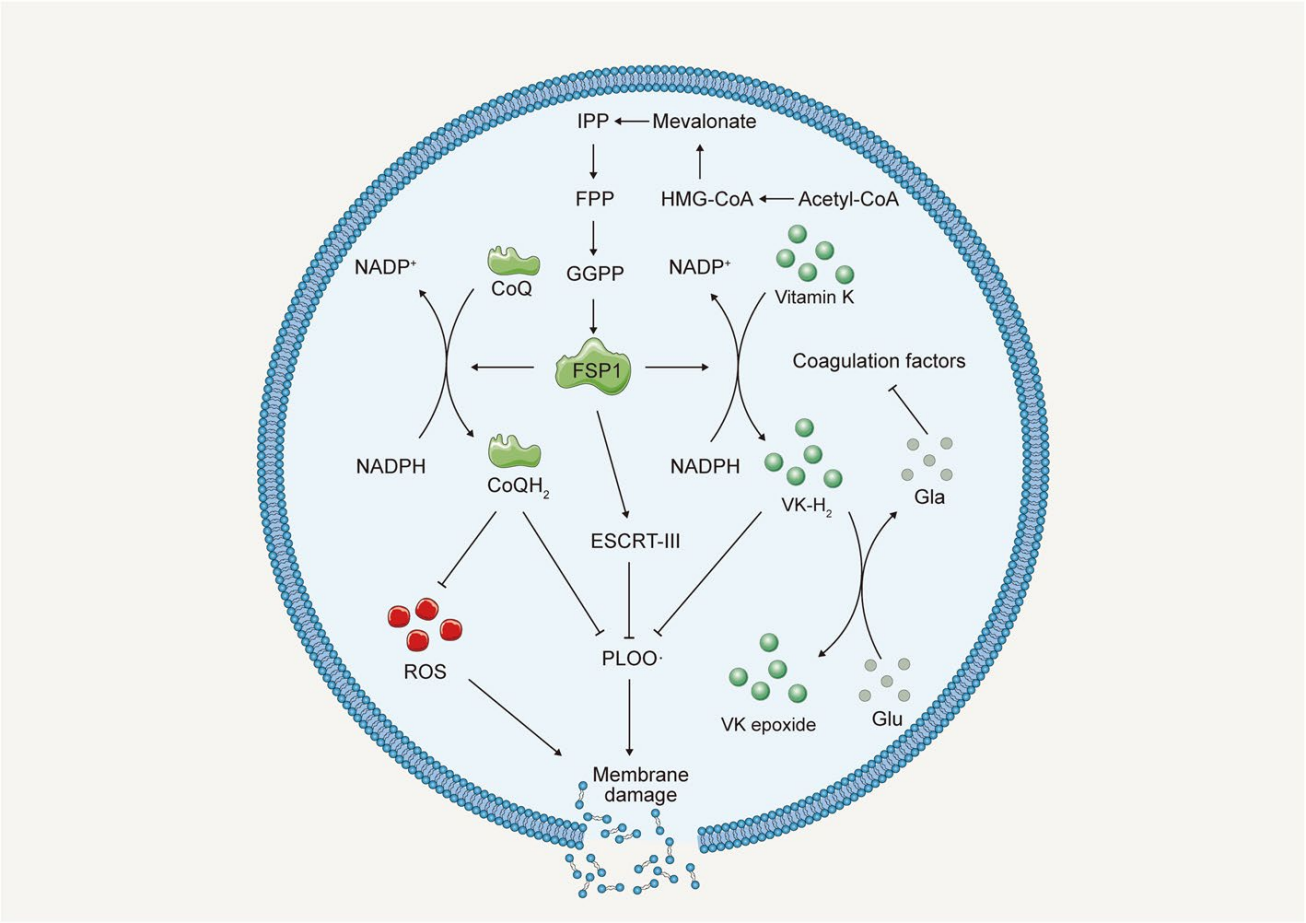
The role of FSP1 in ferroptosis. Abbreviations: VK: Vitamin K

Subsequent studies have confirmed that FSP1 expression confers resistance to ferroptosis but not apoptosis [184]. Further research has revealed that myristoylation of FSP1 accelerates its accumulation on the plasma membrane, where it acts as an oxidoreductase and lipophilic radical-trapping antioxidant, reducing CoQ10 to ubiquinol, thus preventing the peroxidation of PUFAs in the lipid bilayer, and suppressing ferroptosis [181]. Doll’s group has demonstrated that the FSP1-CoQ10-NAD(P)H pathway operates independently with the GPX4 pathway, functioning to either directly scavenge lipid radicals by reducing ubiquinone to ubiquinol, or indirectly regenerate oxidized-tocopheryl radical, thereby suppressing ferroptosis [181]. Such observation elucidates the protective role of extra-mitochondrial ubiquinone in tissues and cells, which has been a long-standing puzzle due to the canonical function of ubiquinone in the mitochondrial electron transport chain [185]. However, the regulation of FSP1 oxidoreductase activity or how its subcellular localization impacts its involvement in various physiological and pathological processes, remains to be further elucidated [180, 181, 183, 186]. Recently, FSP1 was reported that it can convert Vitamin K into the reduced form, hydroquinone (VKH_2_) [187, 188]. Nevertheless, the versatility of FSP1 in oxidizing and reducing substrates, including NADH, NADPH, ubiquinone, and α-tocopherol, implies the sophisticated control of FSP1 activity (Fig. 7).

The prospect of exploiting FSP1 as a therapeutic node to bolster the effectiveness of ferroptosis-based interventions and radiotherapy, notably in the milieu of Kelch-like ECH-associated protein 1 (KEAP1) and Kirsten rat sarcoma virus (KRAS) mutant lung malignancies, has elicited substantial scientific interest [189, 190]. A seminal exploration subjected 30,000 pharmacologically pertinent compounds to rigorous screening, seeking agents capable of precipitating cellular death in cells singularly dependent on FSP1, consequently spotlighting iFSP1 as a robust inhibitor [181]. Another investigation suggested that ferroptosis sensitizer 1 (FSEN1) proficiently inhibits FSP in vitro while also thwarting ferroptosis within the confines of cultured cancer cells [191]. Nonetheless, the necessity for additional investigation is underscored to validate whether FSEN1 can inhibit FSP1 in vivo. It is noteworthy that the applicability of FSEN1 is constricted to human FSP1 [191], thereby decreasing the utility in the scrutiny of mouse FSP1 or neoplastic growth within Genetically Engineered Mouse Models. Anticipated investigative endeavors must strive to establish whether other FSP1 inhibitors unearthed in this study can inhibit mFSP1 and their repercussions on preclinical tumor progression paradigms [191]. Conversely, amplifying FSP1 activity within models of traumatic pathologies, such as ischemia–reperfusion injury, carries immense therapeutic promise. Yet, this field remains relatively unexplored, emphasizing the urgency for concentrated research endeavors to bridge this knowledge gap.

### Other pathways regulating ferroptosis

While the central mechanism governing ferroptosis centers around iron metabolism, lipid peroxidation, GPX4, and FSP1 pathway, it is increasingly apparent that a multitude of ancillary pathways also significantly contribute to the modulation of this distinctive form of cellular death. Recent investigations have unveiled the role of the Hippo-Yes-associated protein (YAP) pathway, AMP-activated protein kinase (AMPK) signaling, and hypoxia pathway in ferroptosis. Fascinatingly, cells cultured at heightened densities demonstrate escalated resistance to ferroptosis triggered by cysteine deprivation and GPX4 inhibition [192–194]. The Hippo-YAP pathway, illustrious for its orchestration of cell proliferation, stress recognition, and organ size moderation, has been scrutinized for its correlation with ferroptosis [195, 196]. Findings delineate that E-cadherin-mediated cell–cell contacts kindle the Hippo signaling pathway via the neurofibromatosis 2 (NF2) tumor suppressor protein, thus curbing nuclear translocation and activity of the transcriptional co-regulator YAP in epithelial cells [193]. YAP, along with its akin homolog TAZ, targets numerous regulators of ferroptosis, encompassing ACSL4 and transferrin receptor TfR1, postulating that the dynamism of the Hippo pathway may modulate cellular responsiveness to ferroptosis, thereby escalating susceptibility upon Hippo suppression and YAP activation [156, 193].

Energy and metabolic stress under normal physiological conditions are crucial for maintaining homeostasis [197]. Disturbances in energy production can result in excessive ROS and cell death [198, 199]. However, interventions mimicking energy stress have been shown to prevent ferroptosis and lipid peroxidation, an effect credited to AMPK, an energy-sensing kinase [112, 200]. The activation of AMPK during glucose deprivation initiates a protective mechanism against ferroptosis, mainly inhibiting PUFA biosynthesis [44, 45]. These findings suggest that such an energy stress program can protect against renal ischemia–reperfusion damage and potentially guard against organ damage related to energy failure.

Initial investigations, suggesting minimal alterations to erastin-induced ferroptosis sensitivity in a 1% oxygen environment, challenged the presumption that hypoxia induces ferroptosis [201]. Hypoxia escalates ROS production via mitochondrial complex III and augments cellular H_2_O_2_ levels, enabling the Fenton reaction [202]. Concurrently, in renal clear cell carcinoma, activation of hypoxia-inducible factors (HIFs) amplifies ferroptosis sensitivity due to GPX4 inhibition, particularly via the HIF2α isoform. Hypoxia initiates HIF2-mediated expression of the hypoxia-inducible lipid droplet-associated protein (HILPDA), resulting in polyunsaturated lipid enrichment [179]. This HIF2-HILPDA-driven heightened sensitivity to ferroptosis suggests an evolutionary mechanism to eradicate hypoxic tumors in the early stages.

Along with the progress, the role of ferroptosis in a proliferating array of disease processes becomes increasingly evident, thereby illuminating novel therapeutic approaches. Operating in concert with other strategies, ferroptosis enriches current treatment paradigms, providing potential solutions to drug resistance challenges. Notwithstanding, our understanding of ferroptosis remains embryonic, with numerous unresolved enigmas left. While it is acknowledged that ferroptosis is initiated by the peroxidation of PUFAs in the cellular membrane and organellar membranes such as the endoplasmic reticulum, the precise mechanisms through which these processes lead to cell death remain uncertain. Furthermore, a thorough investigation into the underlying initiatory and regulatory mechanisms of ferroptosis, the participants involved, and most critically, the complicated interplay between various cell types, persists as an active research domain. Complicating the traditional understanding of ferroptosis, the potential regulation of this process by other metallic ions, such as copper, challenges the dominant position of iron [203]. Thus, deciphering the exact molecular mechanisms and elucidating the role of upstream iron metabolism genes in ferroptosis becomes essential. Furthermore, the identification of distinctive ferroptosis markers is of profound significance to future investigations. In conclusion, the advent of ferroptosis research has inaugurated a promising landscape in disease research, offering considerable potential in devising highly targeted therapies. Nonetheless, much remains to be discovered about the mechanisms of ferroptosis and its role in various diseases, which are important future research directions.

## Physiological functions of ferroptosis

To investigate the biological processes in which ferroptosis is involved, several markers have been developed, including those that detect lipid peroxidation, mitochondrial morphologies, specific gene expression, and TfR1 expression and location [204, 205]. Through the combination of these approaches, ferroptosis has been shown to be critical in tumor suppression, immune surveillance, development, and aging.

### Ferroptosis in tumor suppression and immune functions

The first evidence linking ferroptosis and tumors was the discovery that p53, a well-known tumor suppressor, sensitizes tumor cells to ferroptosis by inhibiting the expression of SLC7A11, a key component of the cystine/glutamate antiporter that mediates cystine transport and represses ROS-induced ferroptosis [8, 206–208]. In human tumors, high expression of SLC7A11 can dampen ferroptosis and diminish the inhibition of tumor growth in xenograft models by acetylation-defective mutant p53 (K117R; K161R; K162R encoding the so-called p53 3KR) [8]. Further investigations revealed that mammalian lipoxygenase family member arachidonate 12-Lipoxygenase (ALOX12) is crucial for p53-dependent ferroptosis. Inactivation or missense mutations of ALOX12, even haploinsufficiency, can ablate p53-mediated tumor growth suppression [149, 209, 210]. Mechanistically, ALOX12 has been identified as a bona fide binding partner of SLC7A11, and its lipoxygenase activity is inhibited in a dosage-dependent manner by SLC7A11 level, which is downregulated by p53 [211]. A nonsynonymous single-nucleotide polymorphism at codon 47 (S47) in tumor protein p53 (TP53 or p53), which is restricted to individuals of African descent, has been found to impair ferroptosis and, therefore, p53-dependent tumor suppression [171]. In cells with S47 mutation, the level of glutamine synthase 2 (GLS2), a glutaminase that converts glutamine into glutamate to induce ferroptosis, is markedly decreased, and the negative regulation of p53 to SLC7A11 is compromised compared to wild-type cells [146, 171]. Moreover, in cells and mice with S47 mutation, the cellular abundance of antioxidants GSH and CoA is elevated, leading to decreased ferroptosis sensitivity [212]. Additionally, the S47 variant of TP53, which has been shown to ablate ferroptosis in cells and mice, also results in iron accumulation in macrophages, altering macrophage cytokine profiles and causing increased susceptibility to bacterial infection and limitation of malarial infection. A recent study found that ALOX12 activation induced by a photosensitizer in cancer cells significantly increases lipid reactive oxygen species and promotes ferroptosis, independent of ACSL4 [213].

MLL4 is an epigenetic regulator and one of the most frequently mutated genes in cancer biology. Depletion of MLL4 in mice promotes features of human precancerous neoplasms. On one hand, MLL4 deficiency suppresses the expression of key lipoxygenases, such as ALOX12, ALOX12B, and ALOXE3, which are involved in driving ferroptosis. On the other hand, lower expression of MLL4 is significantly associated with decreased expression levels of anti-ferroptosis regulators, such as GPX4, SCD1, and GCH1 [214].

The tumor suppressor BRCA1-associated protein 1 (BAP1) is a nuclear de-ubiquitinating enzyme that is responsible for histone 2A modification and gene transcription regulation. BAP1 can regulate ferroptosis primarily through SLC7A11 [215, 216]. Specifically, BAP1 reduces ubiquitinated H2A occupancy on the promoter of SLC7A11, resulting in the repression of SLC7A11 expression. This abrogates cystine uptake and induces ferroptosis [215, 217].

Cysteine desulfurase (NFS1) is an iron-sulfur cluster biosynthetic enzyme that is essential for cancer cell survival when exposed to oxygen [218]. Suppression of NFS1 limits iron-sulfur cluster availability, promoting the iron-starvation response [219] increasing ferroptosis susceptibility [184, 218, 219].

Similar to previous studies that have found excessive accumulation of oxidized PUFA-containing lipids can induce ferroptosis, acidic cancer cells exposed to PUFAs also undergo ferroptosis [220]. PUFAs elevate susceptibility to ferroptosis in the presence of ferroptosis inducers erastin and RSL3, which may be due to diminished upregulation of GPX4 and SLC7A11, as well as apparent downregulation of dihydrofolate reductase (DHFR) and FSP1 [221]. However, unlike acidic cancer cells, uptake of PUFAs from the tumor microenvironment impairs the antitumor ability of CD8^+^ T cells in a mouse melanoma model B16 [222]. PUFAs promote the expression of CD36 on CD8^+^ T cells from human and murine cells, which then activates lipid peroxidation and ferroptosis, reducing cytotoxic cytokine production and antitumor function of CD8^+^ T cells.

Of note, in melanoma and ovarian mouse models, CD8^+^ T cells, when activated by anti-PD-L1 antibody, have been found to drive tumor cell lipid peroxidation and ferroptosis, and this enhanced ferroptosis can promote the anti-tumor function of immunotherapy in turn [223]. In this process, interferon-γ (IFNγ) derived from activated CD8^+^ T cells has been shown to defer the expression of SLC3A2 and SLC7A11, inhibiting tumor cell cystine import and sensitizing tumor cells to ferroptosis. Furthermore, in a melanoma mouse model, IFNγ and AA, one of the PUFAs, have been identified as an anti-tumor combination [15]. IFNγ released from T cells is an activator of the ferroptosis regulator ACSL4 and can accelerate the incorporation of AA into phospholipids, subsequently inducing immunogenic tumor ferroptosis. This suggests that AA found in the tumor microenvironment could potentially be used together with IFNγ as a physiological inducer of ferroptosis.

While ferroptosis is known to serve as a guard in tumor suppression in most research, it appears to play an opposite role in immune functions. Apart from its impact on cytokine production in immune cells such as macrophages and CD8^+^ T cells, ferroptosis also regulates the homeostasis of follicular helper T (TFH) cells [224]. Upregulation of GPX4 by selenium addition has been shown to result in a higher number of TFH cells and elevate humoral immune response in immunized mice and young adults following influenza vaccination. Although evidence suggests that ferroptosis is involved in immunity, further investigation is needed to uncover more links between ferroptosis and immune functions.

### Ferroptosis in development and aging

Due to the delayed development of ferroptosis detection methodologies, the physiological function of ferroptosis remains to be fully understood. Recently, a mouse monoclonal antibody called HNEJ-1 has been designed to specifically identify the most sensitive lipid peroxidation marker, 4-hydroxy-2-nonenal (HNE). This antibody has been used to monitor ferroptosis in different developmental stages of animal models [225]. In Fisher-344 rats, ranging from E9.5 to 2.5 years of age, a significant age-dependent increase in ferroptosis and iron accumulation has been observed in various organs [225]. This increase is also enhanced in a naturally accelerated aging animal model, the Senescence Accelerated Mouse-Prone 8 (SAMP8) mice [225]. Ferroptosis has also been found to occur during rat embryonic erythropoiesis, with its level decreasing as erythrocytes enucleate during the process of maturation. This maturation process is reduced in the presence of ferroptosis inhibitors, Lipro-1 and Fer-1. Inhibition of ferroptosis by melatonin, through neutralizing lipid peroxidation toxicity, has been shown to delay age-related cataract formation [226].

In addition to rats, ferroptosis also affects aging and development in other organisms such as C. elegans and Magnaporthe oryzae. In C. elegans, a reduction in GSH and an increase in ferrous iron typically occur in late life, and suppression of ferroptosis using lipid peroxidation inhibitor liproxstatin or iron chelator salicylaldehyde isonicotinoyl hydrazone has been shown to protect against GSH depletion toxicity, dramatically restrain age-related cell death, and improve the lifespan and healthspan of C. elegans [227]. Regarding to M. oryzae, ferroptosis is crucial for the developmental cell death of conidia during appressorium maturation in rice blast [228]. Inhibition of ferroptosis has been found to dampen the ability of M. oryzae to invade the host.

## Ferroptosis in pathologies

Since the discovery of ferroptosis, evidence has implicated it in a broad array of pathological states including various types of cancer, ischemia–reperfusion (I/R) injury, neurodegenerative disorders, etc. As such, the elucidation of ferroptosis regulatory mechanisms and their relation to human disease has drawn substantial scientific attention. Consequently, therapeutic strategies to modulate ferroptosis, either as inducers to eradicate cancer cells or as inhibitors to protect neurons or ischemic tissues, have unfolded as a promising avenue of translational research.

### Ferroptosis and tumor

Neoplasms encompass an array of genetically divergent subclones. In recent years, burgeoning evidence has underscored the cardinal role of ferroptosis in curbing neoplastic proliferation. A plethora of tumor-suppressive and oncogenic signaling pathways have been identified, which respectively promote or inhibit ferroptosis, offering potential perspectives in cancer therapeutics (Tables 1 & 2).

**Table 1:** Updated therapeutic targets of ferroptosis in tumors

Diseases	Therapeutic targets	Models	Potential mechanisms	References
HCC	HBXIP	In vivo/In vitro	Transcriptionally induced the expression of SCD via coactivating the transcriptional factor ZNF263, resulting in the accumulation of free fatty acids	[229]
	cGAS	In vivo/In vitro	Associate with DRP1 to facilitate its oligomerization	[230]
	Creatine kinase B	In vivo/In vitro	Phosphorylates GPX4 S104	[231]
	HMGCL	In vivo/In vitro	Promote the transcription of DPP4	[232]
	5-HT/3-HA	In vivo/In vitro	Potent radical trapping antioxidants	[233]
	SLC27A5/FATP5	In vivo/In vitro	Enhances the GSR expression in a NRF2-dependent manner	[234]
	ENO1	In vivo/In vitro	Suppresse IRP1 expression	[235]
	PSTK	In vivo/In vitro	Maintain GPX4 activity/promote GSH metabolism/folate biosynthesis	[236]
	ZNF498	In vivo/In vitro	Suppressed p53 transcriptional activation by inhibiting p53 Ser46 phosphorylation	[237]
Liver cancer	HSPA8	In vivo/In vitro	Upregulate the expression of SLC7A11/GPX4	[238]
Pancreatic cancer	TMEM164	In vivo/In vitro	Selectively mediate ATG5-dependent autophagosome formation	[239]
Gastric cancer	CST1	In vivo/In vitro	Interact with GPX4	[240]
	BCL6	In vivo/In vitro	Regulate FZD7/β-catenin/TP63/GPX4 pathway	[241]
	DACT3-AS1	In vivo/In vitro	Mediate SIRT1	[242]
CRC	CYP1B1	In vivo/In vitro	Derive 20-HETE activated the protein kinase C pathway to increase FBXO10 expression	[243]
	TIGAR	In vitro	Mediate ROS/AMPK/SCD1 signaling pathway	[244]
Lung adenocarcinoma	GINS4	In vivo/In vitro	Suppressed p53 stability through activating Snail	[245]
	IGF2BP3	In vivo/In vitro	Dependent on its m^6^A reading domain and binding capacity to m^6^A-methylated mRNAs encoding anti-ferroptotic factors	[246]
Renal cell carcinoma	AIM2	In vivo/In vitro	Promote FOXO3a phosphorylation and proteasome degradation, reduce its transcriptional effect on ACSL4	[247]
Glioma	SNAI3-AS1	In vivo/In vitro	Competitively binds to SND1 and perturbs the m6A-dependent recognition of Nrf2 mRNA 3'UTR by SND1, thereby reducing the mRNA stability of Nrf2	[248]
	PI3K/protein kinase B	In vivo/In vitro	Suppresses the activity of GSK3β and stabilizes Nrf2	[249]
Osteosarcomas	Nrf2	In vivo/In vitro	Interacted with Nrf2, Inhibit GPX4 and xCT expression	[250]
Bone cancer pain	Ferrostatin-1	In vivo/In vitro	Inhibit ERK1/2 and COX-2 expression and prevented the loss of GABAergic interneurons	[251]
Sarcoma	p53^R175H^	In vivo/In vitro	Abrogate BACH1-mediated downregulation of SLC7A11	[252]
Ovarian cancer	FeNP	In vivo/In vitro	Inhibite GPX4	[253]
	CEBPG	In vivo/In vitro	Upregulate SLC7A11	[254]
	NRF2	In vivo/In vitro	Control HERC2 and VAMP8	[255]
	MEX3A	In vivo/In vitro	Mediate p53 protein degradation	[256]
Prostate cancer	RB1	In vivo/In vitro	Upregulate ACSL4/enrich ACSL4-dependent arachidonic acid-containing phospholipids	[257]
	SGK2	In vivo/In vitro	Relieving the inhibitory effect of FOXO1 on GPX4	[258]
ESCC	STC2	In vivo/In vitro	Participate in SLC7A11-mediated ferroptosis in a PRMT5-dependent manner	[259]
Melanoma	CAMKK2	In vivo/In vitro	Activate the AMPK NRF2 pathway	[260]
	PKCβII	In vivo/In vitro	Phosphorylation and activation of ACSL4	[261]
Breast cancer	RUNX1-IT1	In vivo/In vitro	Increase GPX4 expression	[262]
TNBC	HLF	In vivo/In vitro	Activate GGT1 to promote the ferroptosis resistance	[263]

*Abbreviations*: *HCC* Hepatocellular carcinoma, *BCL6* B-cell lymphoma 6, *CRC* Colorectal cancer, *CYP1B1* Cytochrome P4501B1, *AIM2* Melanoma 2, *SGK2* Serum/glucocorticoid regulated kinase 2, *HBXIP* Hepatitis B X-interacting protein, *SCD* Stearoyl-CoA desaturase, *cGAS* Cyclic GMP-AMP synthase, *DRP1* Dynamin-related protein 1, *HMGCL* Hydroxy-methyl-glutaryl-CoA lyase, *DPP4* Dipeptidyl peptidase 4, *5-HT* Tryptophan metabolites serotonin, *3-HT* 3-hydroxyanthranilic acid, *SLC27A5/FATP5* Solute carrier family 27 member 5, *ENO1* Enolase 1, *IRP1* Iron regulatory protein 1, *PSTK* Phosphoseryl-tRNA kinase, *HSPA8* Heat shock protein family A member 8, *TMEM164* Transmembrane protein 164, *ATG5* Autophagy related 5, *CST1* Cysteine protease inhibitor SN, *BCL6* B-cell lymphoma 6, *FZD7* Frizzled 7, *DACT3-AS1* Disheveled binding antagonist of beta catenin3 antisense1, *SIRT1* Sirtuin 1, *CYP1B1* Cytochrome P450 1B1, *TIGAR* TP53-induced glycolysis and apoptosis regulator, *AMPK* AMP-activated protein kinase, *SCD1* Stearoyl-CoA desaturase-1, *IGF2BP3* insulin-like growth factor 2 mRNA binding protein 3, *AIM2* Melanoma 2, *SND1* Staphylococcal Nuclease And Tudor Domain Containing 1, *PI3K* Phosphatidylinositol 3-kinase, *FeNP* Iron nitroprusside, *HERC2* HECT and RLD domain containing E3 ubiquitin protein ligase 2, *VAMP8* Vesicle-associated membrane protein 8, *RB1* Retinoblastoma tumor suppressor protein 1, *SGK2* Serum/glucocorticoid regulated kinase 2, *FOXO1* Forkhead box O1, *RUNX1-IT1* RUNX1 intronic transcript 1, *HLF* Hepatic leukemia factor, *GGT1* Gamma-glutamyltransferase 1, *Nrf2* Nuclear factor erythroid 2–related factor 2, *ESCC* Esophageal squamous cell carcinoma, *STC2* Stanniocalcin 2, *GPX4* Glutathione peroxidase 4, *TNBC* Triple-negative breast cancer, *ACSL4* Acyl-CoA Synthetase Long Chain Family Member 4, *SLC7A11* Recombinant Solute Carrier Family 7, Member 11

**Table 2:** Updated compounds targeting ferroptosis in tumors

Diseases	Compounds	Models	Function	References
HCC	Aspirin	In vivo/In vitro	Restricting NF-κB-activated SLC7A11 transcription	[264]
	EChLESs	In vivo/In vitro	Disrupt mitochondrial membrane potential depolarization and mitochondrial reactive oxygen species	[265]
	AP	In vivo/In vitro	TrxR	[266]
Pancreatic cancer	Wogonin	In vivo/In vitro	Regulate Nrf2/GPX4 axis	[267]
	Copper	In vivo/In vitro	Increase GPX4 ubiquitination and the formation of GPX4 aggregates by directly binding to GPX4 protein cysteines C107 and C148	[268]
	Ponicidin	In vitro	Inhibit the gamma-glutamyl cycle and regulating the polyunsaturated fatty acid metabolism	[269]
Gastric cancer	Polyphyllin I	In vivo/In vitro	Regulate NRF2/FTH1 pathway	[270]
	Sorafenib	In vivo/In vitro	Activate ATF2/ATF2 inhibite SLC7A11 degradation through Upregulate HSPH1	[271]
CRC	NaB	In vivo/In vitro	Mediate CD44/SLC7A11 signaling pathway	[272]
	Erianin	In vivo/In vitro	Induced autophagy-dependent ferroptosis in KRAS^G13D^ CRC cells, while attenuating cell proliferative and metastatic phenotypes	[273]
	Ibrutinib	In vivo/In vitro	Inhibite Nrf2	[274]
	Vitamin D	In vivo/In vitro	Downregulate SLC7A11	[275]
Renal cell carcinoma	URB597	In vivo/In vitro	Inhibite FAAH	[276]
	Salinomycin	In vivo/In vitro	Downregulation of PDIA4	[277]
NSCLC	β-elemene	In vivo/In vitro	Increase the expression of lncRNA H19	[278]
	Timosaponin AIII	In vivo/In vitro	Binding and forming a complex with HSP90, further targeted and degraded GPX4	[279]
	BT	In vitro	Degradation of GPX4 and raising the intracellular Fe^2+^	[280]
	Dihydroartemisinin	In vivo/In vitro	Caused LPO accumulation	[281]
Bladder cancer	EVO	In vivo/In vitro	Decreases GPX4 expression	[282]
Glioblastoma multiforme	Fatostatin	In vivo/In vitro	Inhibit the AKT/mTORC1/GPX4 signaling pathway	[283]
Osteosarcomas	Baicalin	In vivo/In vitro	Interacted with Nrf2, Inhibit GPX4 and xCT expression	[250]
Ovarian cancer	Shikonin	In vivo/In vitro	Upregulate HMOX1	[284]
	Sodium molybdate	In vivo/In vitro	Induce the elevation of the LIP/induces depletion of GSH through mediating the production of NO	[285]
Castration-resistant prostate cancer	BT-Br	In vivo/In vitro	NADPH-binding site inhibitor of Catalase	[286]
TNBC	HCL-23	In vivo/In vitro	Upregulated the expression of HO-1	[287]
Melanoma	Lorlatinib	In vivo/In vitro	Target IGF1R-mediated PI3K/AKT/mTOR signaling axis	[34]
CTCs	Propofol	In vivo/In vitro	Upregulate Nrf2	[288]
FTC	Curcumin	In vitro	Inhibit the growth of FTC by increasing the HO-1 expression	[289]

*Abbreviations*: *NaB* Sodium butyrate, *NSCLC* Non-small NSCLC cell lung cancer, *FAAH* Fatty acid amide hydrolase, *lncRNA* Long noncoding RNA, *BT* Bufotalin, *LPO* Lipid peroxide, *EVO* Evoldiamine, *NO* Nitric oxide, *EChLESs* Eupatorium chinense L, *AP* Alterperylenol, *TrxR* Target the selenoprotein thioredoxin reductase, *HO-1* Heme oxygenase 1, *CTCs* Circulating tumor cells, *FTC* Follicular thyroid cancer, *FTH1* Ferritin heavy chain 1, *ATF2* Activation transcription factor 2, *HSPH1* Heat shock protein-110, *CRC* Colorectal cancer, *FAAH* Fatty acid amide hydrolase, *PDIA4* Protein Disulfide Isomerase Family A Member 4, *HSP90* Heat shock protein 90, *HMOX1* Heme oxygenase 1, *TNBC* Triple-negative breast cancer, *NRF2* NF-E2-related factor 2, *GPX4* Glutathione peroxidase 4, *AKT* Serine/threonine kinase, *mTORC1* mechanistic target of rapamycin complex 1, *GSH* Glutathione, *PI3K* Phosphoinositide 3-kinase, *SLC7A11* Recombinant Solute Carrier Family 7, Member 11

#### Tumor progression

Cancer is a disease characterized by the uncontrolled proliferation of abnormal cells, exhibiting features of unregulated cell growth, invasive expansion, and metastatic potential [290]. Recent years have witnessed remarkable strides in cancer diagnosis and holistic therapeutic approaches such as surgery, chemotherapy, radiation therapy, targeted therapy, and immunotherapy, consequently mitigating cancer mortality rates [291]. Nevertheless, these therapeutic modalities continue to grapple with impediments such as drug resistance, adverse side-effects, and inability to conclusively extirpate metastatic lesions, and the recurrence and metastasis rates of certain tumors persist at elevated levels [10]. For example, the yearly recurrence rate of hepatocellular carcinoma (HCC) post-surgical resection equals or exceeds 10% and escalates to between 70 and 80% after five years [292]. The five-year survival rate for pancreatic ductal adenocarcinoma (PDAC) stands at 10% [293]. Therefore, the exploration of novel therapeutic strategies remains a pressing necessity.

In recent years, emerging research has highlighted the connection between tumor development and ferroptosis [294]. Various oncogenic signaling cascades have been found to conduct the symphony of ferroptosis in malignant cells, and ferroptosis intersects with the functionalities of numerous tumor suppressors, such as the retinoblastoma protein (RB1) and the breast cancer 1 (BRCA1)-associated protein 1 (BAP1) [215, 257]. Compared to their non-malignant counterparts, the proliferation of cancer cells (particularly cancer stem cells) demonstrates a heightened dependency on iron due to its indispensable role in rapid cell multiplication and metabolic activity [295]. By destabilizing iron metabolism within tumorous cells and regulating iron-dependent signaling pathways, it is plausible to provoke ferroptosis in these cells, thereby suppressing tumor expansion and metastasis, and augmenting the efficacy of traditional oncologic treatments [296].

In a recent study, Wang et al. and other researchers discovered that castration-resistant prostate cancer cells are particularly sensitive to ferroptosis, highlighted that the RB/E2F/ACSL4 molecular pathway is a critical regulator of this process [257, 297–299]. Inactivation of the RB1 tumor suppressor gene is common in metastatic castration-resistant prostate cancer, RB1 loss/E2F activation upregulated expression of ACSL4 and enriched ACSL4-dependent AA-containing phospholipids [257].

Numerous other key regulators in neoplastic development have been linked to ferroptosis. The role of Serum/glucocorticoid regulated kinase 2 (SGK2) in promoting prostate cancer metastasis via ferroptosis inhibition was identified by Cheng et al. in 2023 [258, 300]. SGK2 overexpression phosphorylates the Thr-24 and Ser-319 sites of forkhead box O1 (FOXO1) and relieves the inhibitory effect of FOXO1 on GPX4. Moreover, CCAAT/enhancer-binding protein gamma (CEBPG) was established as a novel transcriptional modulator of ferroptosis in ovarian cancer, regulating ferroptosis via transcriptional control of SLC7A11 [254].

Certain neoplasms appear highly reliant on ferroptosis defensive mechanisms for survival under metabolic and oxidative stress. Therefore, disruption of those defenses would be deadly to such cancer cells while sparing normal cells. In 2023, Wang et al. identified heat shock protein family A member 8 (HSPA8) as a crucial host factor that modulates hepatitis B virus (HBV) replication and ferroptosis in liver cancer [238]. HSPA8 suppressed ferroptosis in liver cancer cells by upregulating the expression of SLC7A11/GPX4, decreasing erastin-mediated reactive oxygen species, and accumulating Fe^2+^ in cells in vitro and in vivo [238]. Su et al. identified BTB domain and CNC homology 1 (BACH1) as a cellular factor that strongly interacts with P53^R175H^ [252], and p53^R175H^ acts as a repressor for ferroptosis by abrogating BACH1-mediated downregulation of SLC7A11 to enhance tumor growth [252]. In addition, Chang et al. revealed that STC2 could interact with protein methyltransferase 5 (PRMT5) and activate PRMT5 to participate in SLC7A11 mediated ferroptosis [259]. Ovarian cancer (OC) is the seventh most common malignant tumor and ranks eighth among the causes of cancer death in females [301]. Anandhan et al. also showed that nuclear factor erythroid 2–related factor 2 (NRF2) maintains iron homeostasis by controlling HERC2 (E3 ubiquitin ligase for NCOA4 and F-Box and Leucine-Rich Repeat Protein 5 FBXL5) and vesicle associated membrane protein 8 (VAMP8) (mediates autophagosome-lysosome fusion) [255]. Taken together, the modulation of the iron metabolism pathway serves as a therapeutic means to trigger cancer cell ferroptosis.

#### Therapeutic potential of targeting ferroptosis in cancer

Despite remarkable strides in oncological therapeutics, resistance remains a formidable challenge [302]. A multitude of preclinical and clinical studies are centered on circumventing drug resistance [303]. Intriguingly, ferroptosis has been linked to cancer therapy resistance, and induction of ferroptosis can potentially reverse this resistance. In recent years, certain drugs and compounds have been found to have the ability to induce ferroptosis and demonstrate anti-tumor activity [294].

Wen et al. discovered in 2023 that baicalin affects NRF2 stability through ubiquitin degradation, thereby suppressing NRF2 downstream targets GPX4 and xCT, thereby eliciting ferroptosis [250]. Wogonin is a flavonoid with anticancer activity against various cancers, including pancreatic cancer [304]. In 2023, Liu et al. showed that wogonin upregulates the levels of Fe, lipid peroxidation, and superoxide, and decreases the protein expression levels of ferroptosis suppressor genes, and downregulates level of glutathione in pancreatic cancer cells [267]. Ponicidin could suppress pancreatic cancer cell proliferation via inducing ferroptosis by inhibiting the gamma-glutamyl cycle and regulating the polyunsaturated fatty acid metabolism in SW1990 cells [269]. For several decades, lung cancer has been one of the most common cancers. Many studies have found some antitumor reagents can play an important role in the treatment of lung cancer through ferroptosis [305]. For example, GPX4 inhibitor-Bufotalin (BT), through facilitating the ubiquitination and degradation of GPX4, induces ferroptosis of non-small cell lung cancer (NSCLC) cells [280]. Timosaponin AIII (Tim-AIII), A steroid saponin, can bind to the heat shock protein 90 (HSP90), which further promotes the ubiquitination of GPX4 and thereby degrades GPX4 [279].

Sorafenib, a tyrosine kinase inhibitor, shows an obvious antitumor effect as a ferroptosis inducer in multiple cancers [306]. In 2023, Xu et al. found that activating transcription factor 2 (ATF2) was significantly upregulated by Sorafenib [271]. In this study, heat shock protein family H (Hsp110) member 1 (HSPH1) was identified as a target of ATF2, which can interact with SLC7A11 (cystine/glutamate transporter) and increase its protein stability [271]. In addition, Kang et al. also found salinomycin-induced ferroptosis in renal cell carcinomas (RCCs) [277]. The Disulfide Isomerase Family A Member 4 (PDIA4), as a mediator of salinomycin, suppressed PDIA4 by increasing its autophagic degradation, increasing the sensitivity of RCCs to ferroptosis [277].

As discussed, several drugs (including wogonin, ponicidin, sorafenib and salinomycin) have proferroptotic activity in preclinical models [229, 267, 269, 277]. In the future, targeting ferroptosis with specific drugs is anticipated to play a crucial role in cancer treatment [307]. With advancing understanding of the molecular mechanisms underlying ferroptosis and ongoing research efforts, the potential impact of targeting ferroptosis in cancer therapy can be envisaged in the following aspects: Firstly, targeting ferroptosis holds promise as a strategy to overcome drug resistance, a major obstacle in cancer treatment. By modulating iron metabolism and the signaling pathways related to iron dependency, drugs designed to induce ferroptosis may bypass the resistance mechanisms associated with conventional therapies, exerting pronounced cytotoxic effects on resistant tumor cells [308–310]. Secondly, targeting ferroptosis may enhance treatment efficacy and improve patient outcomes [278, 311]. Given the significant role of ferroptosis in tumor growth, invasion, and metastasis, interventions that interfere with tumor cell iron metabolism and induce ferroptosis have the potential to effectively suppress tumor progression and dissemination, thereby improving treatment responses and prognoses, ultimately leading to better survival rates and quality of life for patients [312, 313]. Furthermore, targeting ferroptosis could offer new avenues for personalized cancer therapy [314]. The heterogeneity of tumors and individual variability often render conventional treatment modalities suboptimal for all patients. By targeting iron metabolism and signaling pathways, drugs designed to induce ferroptosis can enable tailored treatment approaches based on individual patient characteristics, providing more precise and effective therapeutic strategies [315–317]. Lastly, targeting ferroptosis may emerge as a critical component of combination therapies. Combinatorial approaches have become a major trend in cancer treatment, as they can enhance therapeutic efficacy while reducing side effects. By integrating drugs targeting ferroptosis with other treatment modalities such as chemotherapy, immunotherapy, or targeted therapies, synergistic effects can be achieved, further augmenting treatment responses [318–320]. In summary, targeting ferroptosis with specific drugs holds tremendous potential in future cancer treatment. This approach offers the prospects of overcoming drug resistance, improving treatment efficacy, enabling personalized therapy, and integrating with other treatment modalities, thereby paving the way for enhanced outcomes and advancements in cancer care.

### Ferroptosis and ischemic/reperfusion related diseases

I/R injury is a complex physiological event that occurs when blood supply to a tissue or organ is disrupted and then subsequently restored [321, 322]. This process, while seemingly paradoxical, can lead to significant tissue damage and cell death, often exceeding the initial injury caused by ischemia alone[323, 324]. The initial ischemic phase can be induced by a variety of causes, such as a blockage in the blood vessels due to a clot or plaque, or a systemic reduction in blood flow due to shock, cardiac arrest or organ surgeries [321]. The lack of blood flow deprives the tissue of oxygen and nutrients, leading to a state of hypoxia and nutrient deprivation. This can result in cellular dysfunction and, if prolonged, irreversible cell damage and death [325]. The subsequent reperfusion stage is necessary to deliver oxygen and nutrients to the ischemic tissue, however, it paradoxically leads to further tissue damage. This process is due to the sudden influx of oxygen and nutrients, which can result in the overproduction of ROS and the initiation of inflammatory responses [326, 327]. The ROS can cause oxidative damage to cellular components, while the inflammatory responses can lead to further cell death and tissue damage [328, 329].

The type of cells and tissues affected by I/R injury can vary widely, and include the heart (as in myocardial infarction), brain (as in stroke), kidneys (as in acute kidney injury), liver (as in hepatic I/R injury), and intestines (as in mesenteric ischemia) [322, 330–334]. At the cellular level, I/R injury can lead to various forms of cell death, including necrosis, apoptosis, and autophagy [335, 336]. Recently, ferroptosis has been implicated in I/R injury [337–339]. It has been proposed that the oxidative stress and inflammation caused by I/R injury may trigger ferroptosis, thereby exacerbating tissue damage [48]. This has led to the hypothesis that targeting ferroptosis could be a novel therapeutic strategy for mitigating I/R injury. We have summarized the potential therapeutic targets on I/R injury in Table 3.

**Table 3:** Updated therapeutic targets of ferroptosis in ischemia–reperfusion injury

Diseases	Therapeutic targets	Models	Potential mechanisms	References
Myocardial I/R injury	Alox15/15-HpETE	In vivo/In vitro	Promote the binding of Pgc1α to the ubiquitin ligase ring finger protein 34	[339]
	MALT1	In vivo/In vitro	Enhance the Nrf2/SLC7A11 pathway	[340]
	Mir-196c-3p	In vivo/In vitro	Inhibit NOX4, P53, and LOX expression	[341]
	lncRNA Mir9-3hg	In vivo/In vitro	Via the Pum2/PRDX6 axis	[342]
	Ubiquitin-specific protease 7	In vivo/In vitro	Activate the p53/TfR1 pathway	[343]
	ATF3	In vivo/In vitro/ serum samples(patients)	Regulate FANCD2	[344]
	MiR-375-3p	In vivo/In vitro/ serum samples(patients)	Regulate GPX4	[345]
	SEMA5A-IT1	In vivo/In vitro/ serum samples(patients)	Regulate BCL2 and SLC7A11 through sponging miR-143-3p	[346]
	A(1) and A(2b) adenosine receptors	In vivo/In vitro/ serum samples(patients)	Regulate GPX4	[347]
	MiR-199a-5p	In vivo/In vitro/ serum samples(patients)	Inhibite Akt/eNOS signaling pathway	[348]
	Transferrin and glutamine	In vivo/In vitro	Ferroptosis inducer	[146]
	USP22	In vivo/In vitro	Regulate SIRT1/p53/SLC7A11 axis	[349]
	USP7	In vivo/In vitro	Upregulate p53/TfR1 pathway	[343]
	FPN	In vivo/In vitro	Regulate iron homeostasis	[350]
	DNMT-1	In vivo/In vitro	Promote NCOA4-mediated ferritinophagy	[351]
	OxPCs	In vivo/In vitro	Suppress GPX4 activity	[352]
	ELAVL1	In vivo/In vitro	Promote autophagic ferroptosis	[353]
	MiR-135b-3p	In vivo/In vitro	Downregulate GPX4 expression	[354]
	LncAABR07025387.1	In vivo/In vitro	Sponge miR-205 to enhance ACSL4 expression	[355]
	LncRNA Mir9-3hg	In vivo/In vitro	Regulate Pum2/PRDX6 axis	[342]
Cerebral I/R injury	CDGSH iron sulfur domain 2	In vivo/In vitro	Upregulate the expression of GPX4, cystine-glutamate antiporter and glutathione	[356]
	PUM2	In vivo/In vitro	Suppress SLC7A11 via inhibiting expression of SIRT1	[357]
	TNFAIP1	In vivo/In vitro	Nrf2/GPX4‑mediated ferroptosis	[358]
	POU2F2	In vivo/In vitro	Activate Sestrin2	[359]
	BACH1	In vivo/In vitro	Activate KDM4C-mediated COX2 demethylation	[360]
	Tau	In vivo	Tau-iron interaction, inhibit iron overload	[361]
	Ferritin	In vivo/In vitro	Regulate p53 and SLC7A11	[362, 363]
	Mitochondrial ferritin	In vivo	Inhibit iron overload, inhibit lipid peroxidation	[364]
	NCOA4 and USP14	In vivo/In vitro	Promote ferritinophagy	[362]
	UBIAD1	In vivo/In vitro	Inhibit lipid peroxidation	[365]
	PGE2	In vivo	Inhibit iron accumulation and lipid peroxidation	[366]
	SAT1	In vivo/In vitro	Transcriptional target of p53, induce lipid peroxidation	[367]
	Thrombin	In vivo/In vitro	Initiate esterifification of ACSL4	[368]
	LncRNA PVT1/miR-214	In vivo/In vitro	Inhibit TfR1 and p53	[369]
Hepatic I/R injury	Mu-opioid receptor	In vivo/In vitro	Regulate the HIF-1α/KCNQ1OT1 axis	[370]
	MiR-29a-3p	In vivo/In vitro	Via Iron Responsive Element Binding Protein 2, Downregulate IREB2 expression	[371]
	MET	In vivo/In vitro/Human samples	Disrupt iron metabolism	[372]
	HUWE1	In vivo/In vitro/Human samples	Target TfR1 for proteasomal degradation	[373]
Renal I/R injury	MiR-20a-5p	In vivo/In vitro	Inhibit of ACSL4-dependent ferroptosis	[374]
	Trim21	In vivo/In vitro	Ubiquitylate GPX4	[375]
	lncRNA TUG1	In vivo/In vitro	Interact with SRSF1 to regulate ASCL4	[376]
	ALR	In vitro	Anti-oxidant, upregulate GPX4 expression	[377]
	Panx1	In vivo/In vitro	Regulate HO-1, NCOA4 and FTH1	[378]
	CIRBP	In vivo/In vitro	Regulate ELAVL1 to promote ferritinophagy	[379]
	Legumain	In vivo/In vitro	Promote degradation of GPX4	[380]
	IDO	In vitro	Induce AhR-mediated ferroptosis	[381]
	LSD1	In vivo/In vitro	Upregulate TLR4/NOX4 pathway	[382]
	MiR-182-5p and miR-378-3p	In vivo/In vitro	Downregulate GPX4 and SLC7A11 expression	[383]
	MiR-3587	In vitro	Downregulate HO-1 expression	[384]
Lung I/R injury	Nrf2	In vivo/In vitro	Upregulate SLC7A11-related axis	[385–387]
	p53	In vivo/In vitro	Regulate Nrf2 signaling pathway	[388]
Spinal cord I/R injury	USP11	In vivo/In vitro	Deubiquitinate Beclin 1	[389]

*Abbreviations*: *Alox15* 15-lipoxygenase-1, *15-HpETE* 15-Hydroxyicosa-5,8,11,13-tetraenoic acid, *MALT1* Mucosa-associated lymphoid tissue lymphoma translocation protein 1, *MiR-196c-3p* MicroRNA-196c-3p, *lncRNA Mir9-3hg* lncRNA MiR9-3 host gene, *USP7* Ubiquitin-specific protease 7, *ATF3* Activating transcription factor 3, *MiR-375-3p* MicroRNA-375-3p, *SEMA5A-IT1* Semaphorin 5A, *MiR-199a-5p* MicroRNA-199a-5p, *USP22* Ubiquitin-specific protease 22, *FPN* Ferroportin, *DNMT-1* DNA methyltransferase 1, *OxPCs* Oxidized phosphatidylcholines, *ELAVL1* ELAV Like RNA Binding Protein 1, *MiR-135b-3p* MicroRNA-135b-3p, *PUM2* Pumilio RNA Binding Family Member 2, *TNFAIP1* TNF Alpha Induced Protein 1, *POU2F2* POU Class 2 Homeobox 2, *BACH1* The transcription factor BTB and CNC homology 1, *NCOA4* Nuclear receptor coactivator-4, *USP14* Ubiquitin-specific protease 14, *UBIAD1* UbiA Prenyltransferase Domain Containing 1, *PGE2* Prostaglandin E2, *SAT1* Spermidine/Spermine N1-Acetyltransferase 1, *PVT1* Plasmacytoma variant translocation 1, *MiR-29a-3p* MicroRNA-29a-3p, *MET* MET Proto-Oncogene, Receptor Tyrosine Kinase, *HUWE1* UBA and WWE Domain Containing E3 Ubiquitin Protein Ligase 1, *MiR-20a-5p* MicroRNA-20a-5p, *Trim21* Tripartite motif containing-21, *lncRNA TUG1* Long non-coding RNA taurine-upregulated gene 1, *ALR* Augmenter of liver regeneration, *Panx1* Pannexin 1, *CIRBP* Cold Inducible RNA Binding Protein, *IDO* Indoleamine, *LSD1* Lysine-specific histone demethylase 1A, *MiR-182-5p* MicroRNA182-5p, *MiR-378-3p* MicroRNA-378-3p, *MiR-3587* MicroRNA-3587, *Nrf2* Nuclear factor erythroid 2-related factor 2, *p53* Cellular tumor antigen p53, *USP11* Ubiquitin-specific protease 11

#### Myocardial I/R injury

Acute myocardial infarction (MI), a paramount life-threatening coronary event, afflicts in millions of individuals annually, and these numbers continue to rise worldwide [390–392]. Despite the mitigating mortality and morbidity rates concomitant with the rapid evolution of medical technologies, the heart failure precipitated by MI continues to remain alarmingly high, imposing a substantial financial and societal burden on individuals and communities [393, 394]. I/R injury is an important pathological process during MI treatment [395]. MI-induced myocardial ischemia results in inadequate oxygen supply to the myocardial cells, while oxidative stress during reperfusion exacerbates cellular damage [396]. Studies have found that insufficient oxygen supply and oxidative stress caused by ischemia lead to the excessive accumulation of intracellular iron ions, increasing the likelihood of ferroptotic cell death [396]. Iron contribute to myocardial cell injury through oxidative stress reactions and lipid peroxidation mechanisms [397]. Subsequently, MI is commonly remedied with prompt and efficacious myocardial reperfusion, typically through thrombolytic therapy or primary percutaneous coronary intervention (PPCI) [398]. Reperfusion therapy exacerbate damage to the myocardial tissue, through oxidative stress, inflammatory reaction, disorder of energy metabolism, causing cell death, myocardial stunning, arrhythmia, myocardial vertigo [399, 400]. Xiao-Hui Ma and colleagues have elucidated the role of ischemia in inducing a specific oxidative-reductive reaction involving PUFAs-containing phospholipids within myocardial cells [401]. This reaction serves as a pivotal initiating signal for the robust initiation of oxidative damage during reperfusion [401]. They have proposed ALOX15 as the primary mediator responsible for the ischemia-induced peroxidation of phospholipids [401]. Additionally, another study has provided evidence demonstrating that 15-hydroperoxyeicosatetraenoic acid (15-HpETE), an intermediate metabolite derived from AA through the action of ALOX15, acts as a critical trigger for ferroptosis in cardiac myocytes [339]. Other targeted therapeutic strategies associated with various genes associated with ferroptosis have also been studied in myocardial I/R injury models. Research has revealed that inhibition of MALT1 can reduce I/R-induced myocardial iron efflux by enhancing the NRF2/SLC7A11 pathway [340]. Inhibiting the expression of key ferroptotic genes NOX4, P53, and LOX can reduce ferroptosis in myocardial cells and improve cardiac function [341]. By modulating the PUM2/PRDX6 axis, it is possible to suppress myocardial iron deposition, thereby alleviating I/R-induced cardiac injury and improving cardiac function [342]. Tang et al. identified a novel pathway involving USP7/P53/TfR1 in the hearts of rats subjected to I/R treatment, where upregulation of USP7 promoted iron deposition through activation of the P53/TfR1 pathway [343]. Small molecule drugs targeting ferroptosis have shown promising potential in myocardial I/R injury. The ALOX15-specific inhibitor ML351 has been shown to elevate the protein level of Pgc1α, suppress cardiomyocyte death, protect damaged myocardium, and promote cardiac function recovery [339]. Xanthohumol (XN), an isoenoic flavonoid derived from hops, exhibits cardioprotective effects by mitigating ferroptosis through lipid peroxidation and ROS generation, chelating iron ions, modulating NRF2 protein levels, and regulating GPX4 protein expression [402]. Another study demonstrated that dapagliflozin, the sodium glucose co-transporter 2 (SGLT2) inhibitor, exerts inhibitory effects on ACSL4, which suppresses ferrosome formation, by upregulating the SLC7A11/GPX4 axis and ferritin heavy chain (FTH) expression [403]. Further research and understanding of the mechanism of ferroptosis, especially identifying effective compounds targeting ferroptosis, in myocardial I/R injury will help reveal the pathogenesis of myocardial I/R injury and provide for the development of more effective treatment strategies.

#### Cerebral I/R injury

Ischemic stroke, also known as cerebral infarction, is a neurological disorder caused by localized cerebral, spinal cord, or retinal infarction [404]. It is a major public health issue with a high incidence, resulting in disability and death, with millions of new cases reported annually [405]. Survivors often experience long-term physical, cognitive, and emotional impairments [405]. Additionally, it also imposes a significant economic burden on healthcare systems and societies. Ischemic stroke and subsequent reperfusion injury elicit oxidative stress, which results in aberrant intracellular iron ion accumulation, consequently triggering ferroptosis [406]. Guo et al. also found that rats with cerebral I/R injury had severe brain damage and neurological deficits, accompanied by typical molecular features of ferroptosis, including GSH disturbances, abnormal accumulation of iron, and increased lipid peroxides. These observations underscore the significance of comprehending and intervening in the mechanisms underlying ferroptosis, offering potential avenues to enhance therapeutic efficacy in the context of stroke management. Hu et al. showed that upregulation of CDGSH iron-sulfur domain 2 alleviates cerebral I/R injury through activation of the NRF2/HO-1 pathway, which is a key factor in maintaining cellular redox homeostasis and lipid and iron metabolism [356]. Another study found that in cells exposed to I/R injury, the knockdown of Retinoid X receptor γ (RXRγ) resulted in the downregulation of GPX4 expression and the upregulation of COX-2 and ROS levels [407]. Researchers therefore suggest that the transcriptional activation of GPX4, mediated by RXRγ, may contribute to the inhibition of ferroptosis in the context of cerebral I/R injury [407]. Furthermore, the absence of NCOA4 significantly abrogated ferritinophagy induced by I/R injury, thereby suppressing ferroptosis [362]. Numerous inhibitors of ferroptosis have shown promising effects in ameliorating stroke. Srs11-92 (AA9), a Fer-1 analog, reduced oxidative stress and neuroinflammation in neurons subjected to OGD/R by activating the NRF2 pathway [408]. Researchers believe that AA9 has potential as a therapeutic candidate for protecting against neuronal damage in stroke and other neurological diseases, by targeting NRF2-mediated oxidative stress and neuroinflammation [408]. Dl-3-n-butylphthalide, a compound derived from celery seed, regulates ferroptosis through SLC7A11/GSH/GPX4 pathway to achieve neuroprotective effect on I/R injury [409]. The administration of proanthocyanidins (PC), a class of organic antioxidants, upregulates the expression of GPX4 and SLC7A11 while downregulating the expression of TFR1, thereby exerting an inhibitory effect on ferroptosis. Proanthocyanidins (PC), as organic antioxidants, upregulate the expression of GPX4 and SLC7A11 while downregulating the expression of TFR1, thereby inhibiting ferroptosis and ameliorating cerebral I/R injury [410]. The continued investigation and development of ferroptosis inhibitors hold great promise for improving the treatment and management of stroke, providing new avenues for reducing the devastating consequences of this cerebrovascular disorder. In summary, ferroptosis plays a crucial role in cerebral I/R injury, and understanding its mechanisms can aid in the development of new therapeutic strategies to protect brain cells from oxidative stress and cell death. However, further research is still needed to explore the specific mechanisms and potential therapeutic targets of ferroptosis in cerebral I/R injury.

#### Hepatic I/R injury

Hepatic I/R injury is mainly caused by liver surgery, such as partial hepatic resection and liver transplantation, where severe hepatic I/R injury after liver transplantation leads to acute or chronic rejection and even transplant failure by inducing inflammation and oxidative stress [411, 412]. Hepatic I/R injury is frequently associated with inflammation and oxidative stress, which can precipitate systemic inflammatory response syndrome (SIRS) or multiple organ dysfunction syndrome (MODS), exacerbating the patient's condition and leading to organ damage and functional impairment) [413]. Several studies have explored the involvement of ferroptosis in hepatic I/R injury and its potential as a therapeutic target [414]. The study by Ye et al. provides confirmation that MCTR1 attenuates hepatic ischemia–reperfusion injury caused by ferroptosis through the promotion of NRF2 expression [415]. Guo et. Declared that transmembrane member 16A (TMEM16A), a component of hepatocyte Ca^2+^-activated chloride channel, exacerbates hepatic I/R injury through the promotion of GPX4-dependent ferroptosis, and interrupting the TMEM16A-GPX4 interaction or inhibiting TMEM16A in liver cells may represent promising therapeutic strategies for the treatment of hepatic I/R injury [416]. The HECT domain-containing ubiquitin E3 ligase HUWE1 (also known as MULE) has emerged as a promising protective factor in mitigating acute liver injury by counteracting abnormal iron accumulation and inhibiting ferroptosis [373]. There is currently limited development and application of small molecule drugs targeting ferroptosis in the treatment of hepatic ischemia–reperfusion injury. Neutrophil membrane-coated taurine nanoparticles increased the expression of SLC7A11 and GPX4, and decreased the expression of Ptgs2, suggesting that nano-taurine has a targeted therapeutic effect on hepatic I/R injury by inhibiting inflammation, oxidative stress and ferroptosis [417]. Dimethyl fumarate (DMF), a therapeutic agent utilized in the treatment of relapsing–remitting multiple sclerosis, demonstrates inhibitory effects on ferroptosis through activation of the NRF2/SLC7A11/HO-1 axis, thereby conferring protection against hepatic I/R injury [418]. Although the relationship between hepatic I/R injury and ferroptosis has been confirmed, the development and application of therapies and drugs targeting ferroptosis are still limited. Further research is needed to explore the regulation of iron metabolism, oxidative stress, and other molecular targets associated with ferroptosis, aiming to discover more effective treatment strategies and opportunities for the management of hepatic I/R injury.

#### Renal I/R injury

Renal I/R injury can be triggered by multiple factors including renal artery obstruction, hypotension, shock, and surgical interventions [419, 420]. This injury culminates in renal tissue ischemia, hypoxia, disruption of tubular and vascular architecture, provoking inflammatory response and cellular death, ultimately culminating in renal dysfunction [420]. Renal I/R injury can cause electrolyte imbalances and discomfort, while requiring patients to undergo multiple treatments like hemodialysis or kidney transplantation. This significantly affects their quality of life and imposes a financial burden [421]. The molecular mechanisms regulating iron metabolism and ferroptosis have been found to play a crucial role in the development and treatment of renal I/R injury [422]. A recent study suggests that miR-20a-5p has potential therapeutic applications in kidney transplantation by inhibiting ACSL4-dependent ferroptosis [374]. TRIM21 exhibits elevated expression in kidney tissues undergoing renal I/R injury. Downregulation of TRIM21 mitigated renal I/R injury and protected renal function [375]. The involvement of cold-inducible RNA-binding protein (CIRBP) in acute kidney injury has been suggested in another research, which proposed that ferritinophagy-mediated ferroptosis may be responsible for the enhanced ischemic kidney injury observed in the presence of CIRBP [379]. In ischemic kidney injury, iron chelators such as deferoxamine, deferiprone, and lipophilic antioxidants have been shown to inhibit lipid peroxidation and protect against cell damage [423]. These agents can target lipoprotein-1 (Lip-1), ferristatin-1, as well as vitamins and flavonoids, which are involved in antioxidant defense [423, 424]. These studies also suggest that molecular mechanisms regulating iron metabolism and ferroptosis may play an important role in the treatment of acute ischemic kidney injury. Some pharmacological agents targeting ferroptosis have also been investigated. Cyanidin-3-glucoside (C3G), a typical flavonoid, can activate AMPK pathway to inhibit ferroptosis in renal tubular cells after I/R injury [425]. Qi et al. found that the regulatory effect of MGZ on the Mitoneet-mediated iron apoptosis pathway, highlighting its potential role in renal protection [426]. Methods such as interfering in iron metabolism, modulating antioxidant defenses, and inhibiting iron-related pathways have shown promising results in preclinical studies aimed at alleviating renal I/R injury and preserving renal function (Table 4). However, further research is needed to fully understand the underlying mechanisms driving renal I/R-induced ferroptosis and to develop effective treatment strategies.

**Table 4:** Updated Compounds targeting ferroptosis in ischemia reperfusion injury

Diseases	Compounds	Models	Function	References
Myocardial I/R injury	Polydopamine Nanoparticles	In vivo/In vitro	Inhibit Fe accumulation and restore mitochondrial functions	[427]
	CVP	In vivo/In vitro	Decrease intracellular Fe2 + level, enhance GPX4 expression	[428]
	Atorvastatin	In vivo/In vitro	Regulate SMAD7/Hepcidin expression	[429]
	Dexmedetomidine	In vivo/In vitro	Via AMPK/GSK-3β/Nrf2 axis	[430, 431]
	Resveratrol	In vivo/In vitro	Decrease TfR1 expression, and increase the expressions of FTH1 and GPX4	[432]
	Shenmai	In vivo/In vitro	Targete Nrf2/GPX4 Signalling	[433]
	Xanthohumol	In vivo/In vitro	Decrease the production of lipid peroxidation and ROS	[402]
	HJ11	In vivo/In vitro	Suppress ACSL4	[434]
	Puerarin	In vivo/In vitro	Reduce the expression of Ptgs2 mRNA, and increase GPX4	[435]
	Dapagliflozin	In vivo/In vitro	Upregulate the SLC7A11/GPX4 axis and FTH and inhibite ACSL4	[403]
Cerebral I/R injury	Vitexin	In vivo/In vitro	Regulate Keap1/Nrf2/HO-1 signaling pathway	[436]
	Ferrostatin-1	In vivo/In vitro	Upregulate GPX4 expression and inhibit COX-2 expression	[437]
	Oxysophoridine	In vivo/In vitro	Decrease ACSL4 / transferrin 1 protein and increase ferritin 1 / GPX4	[438]
	Srs11-92	In vivo/In vitro	Regulate Nrf2 signal pathway	[408]
	Dl-3-n-butylphthalide	In vivo/In vitro	Regulate SLC7A11/GSH/GPX4 signal pathway and PDGFRβ/PI3/Akt signal pathway	[409]
	Procyanidins	In vivo/In vitro	Activate the Nrf2/HO-1 pathway	[410]
	Selenium compounds	In vivo/In vitro	Drive GPX4 expression	[24, 439, 440]
	Carvacrol	In vitro	Upregulate GPX4 expression	[441]
	Rehmannioside A	In vivo/In vitro/Human samples	Activate SLC7A11/GPX4 axis	[442]
	Galangin	In vivo/In vitro	Activate SLC7A11/GPX4 axis	[443]
	Carthamin yellow	In vivo	Inhibit ACSL4 expression	[444]
	Kaempferol	In vitro	Activate Nrf2/SLC7A11/GPX4 axis	[445]
	Liproxstatin-1	In vivo/In vitro/Human samples	Inhibit lipid peroxidation	[165, 338, 361, 446, 447]
Liver I/R injury	Nano-taurine	In vivo/In vitro	Upregulate SLC7A11 and GPX4	[417]
	Dimethyl fumarate	In vivo/In vitro	Activate the NRF2/SLC7A11/HO-1 axis	[418]
	α-tocopherol	In vivo	Inhibit lipid peroxidation	[448]
Renal I/R injury	Paeoniflorin	In vivo/In vitro	Upregulate Slc7a11 in the glutathione pathway	[449]
	LoxBlock-1 or Curcumin	In vivo	Facilitate ACSL/GPx4 signaling	[450]
	Cyanidin-3-glucoside	In vivo/In vitro	Regulate AMPK pathway	[425]
	Legumain	In vivo	Facilitate chaperone-mediated autophagy	[380]
	Vitamin K1	In vivo	Inhibitor of ferroptosis	[451]
	Mitoglitazone	In vivo	Upregulate the expression of GPX4	[426]
	Pachymic acid	In vivo	Upregulate Nrf2 signaling pathway	[452]
	16–86	In vivo/In vitro	Inhibit lipid peroxidation	[453]
	XJB-5–131	In vivo	Inhibit lipid peroxidation/anti-oxidant	[454]
	Quercetin	In vivo/In vitro	Inhibit ATF3/SLC7A11/GPX4 axis	[424]
	Nec-1f	In vivo/In vitro	Inhibit RIPK1 kinase activity and ferroptosis	[455]
	Entacapone	In vivo/In vitro	Upregulate SLC7A11 repression	[456]
Lung I/R injury	Salidroside	In vivo/In vitro	Activate the Nrf2/SLC7A11 signaling axis	[457]
	Isoliquiritin apioside	In vivo/In vitro	Via a Hif-1α-dependent manner	[458]
	Lidocaine	In vivo/In vitro	Regulate the p38 MAPK pathway	[459]
	Irisin	In vivo/In vitro	Upregulate Nrf2/HO-1 axis/upregulate GPX4	[460, 461]
	Rosiglitazone	In vivo/In vitro	Inhibit ACSL4 expression	[338, 447]

*Abbreviations*: *CVP* Chuanminshen violaceumis polysaccharide, *SMI* Shenmai injection, *HJ11* a novel traditional Chinese medicine developed from the appropriate addition and reduction of Si-Miao-Yong-An decoction, *SRS16-86* third-generation ferrostatin, *XJB5-131* a mitochondria-targeted ROS and electron scavenger, *Nec-1f* a highly selective inhibitor of RIPK1 (receptor interacting protein kinase 1)

In conclusion, emerging evidence strongly supports the pivotal role of ferroptosis in the pathogenesis of I/R injury, highlighting its potential as a promising therapeutic target. However, the regulatory mechanisms underlying ferroptosis in the context of I/R injury remain incompletely elucidated. Further research is warranted to unravel the new pharmacological mechanisms, toxicity profiles, side effects, and optimal dosages of ferroptosis inhibitors through rigorous preclinical and clinical investigations. Therefore, it is imperative to comprehensively understand the regulatory mechanisms governing ferroptosis in I/R injury and identify safe and effective targeting strategies for modulating ferroptosis regulators to mitigate I/R injury.

### Ferroptosis and neurodegenerative diseases

Neurodegenerative afflictions, encompassing Alzheimer's disease (AD), Parkinson's disease (PD), Huntington's disease (HD), and Amyotrophic Lateral Sclerosis (ALS), constitute a cohort of incapacitating disorders marked by the progressive neuronal attrition and the attendant regression in cognitive and motor functionalities. Despite prodigious research, the integral mechanisms instigating and fostering these diseases remain elusive. Recently, ferroptosis has been unveiled as a plausible mechanism bearing implications for the pathogenesis of neurodegenerative diseases [7, 462]. The incorporation of ferroptosis in the context of neurodegenerative diseases has garnered increasing recognition due to the burgeoning evidence associating dysregulated iron metabolism, compromised antioxidant defenses [463], and amplified lipid peroxidation [464] with the pathogenesis of these disorders. Prior studies have manifested alterations in iron distribution and accrual in specific cerebral regions affected by neurodegeneration [465–468]. Furthermore, heightened levels of lipid peroxidation markers and diminished antioxidant capacity have been discerned in the brains of individuals afflicted with neurodegenerative diseases [469, 470], suggesting a potential role of ferroptosis in the selective neuronal loss (Tables 5 & 6).

**Table 5:** Updated therapeutic targets of ferroptosis in neurodegenerative disease

Diseases	Therapeutic targets	Models	Potential mechanisms	References
AD	Fe^2+^/Fe^3+^	In vivo	Fe^3+^/Fe^2+^ ratio was mainly observed in amyloid plaque regions	[471]
	PS1	In vitro	Promote the expression of GPX4	[472]
	ALDH2	In vivo/In vitro	Inhibition of ACSL4-dependent ferroptosis	[473]
	Apolipoprotein E	In vivo/In vitro	ApoE signals to activate the PI3K/AKT pathway that then inhibits the autophagic degradation of ferritin, thus averting iron-dependent lipid peroxidation	[469]
	GPX4	In vivo	Gpx4 overexpression was effective in improving behavior function and reducing neurodegeneration	[474]
	NOX4	In vivo/In vitro	NOX4 promotes ferroptosis of astrocytes by oxidative stress-induced lipid peroxidation via the impairment of mitochondrial metabolism in Alzheimer's diseases	[475]
	GSK-3β	In vivo	Ferroptosis can lead to abnormal aggregation of tau protein and might be a promising therapeutic target of tauopathies	[476]
PD	GPX4	In vivo	Midbrain dopamine oxidation links ubiquitination of glutathione peroxidase 4 to ferroptosis of dopaminergic neurons	[477]
	ACSL4	In vitro	ACSL4 is pivotal for ferroptosis induced by iron and PUFA dyshomeostasis in dopaminergic neurons	[478]
	SEC24B	In vitro	Microglia ferroptosis is regulated by SEC24B and contributes to neurodegeneration	[479]
	LRRK2	In vitro	LRRK2 protects immune cells against erastin-induced ferroptosis	[480]
	Alpha synuclein	In vitro	Endogenous levels of α-synuclein can determine the sensitivity of dopaminergic neurons to ferroptosis	[481]
	SNX5	In vivo/In vitro	Decrease of GPX4	[482]
	PPARδ	In vitro	PPARδ attenuates 6-OHDA-induced neurotoxicity by preventing intracellular iron accumulation	[483]
	Nrf2	In vivo	Mediated by decreasing the FPN1 level on brain microvascular endothelial cells, thus hindering the process of iron entry into the brain	[484]
	Nurr1	In vivo	Nurr1 deficiency results in an increase in CD74 expression, thereby leading to the destruction of dopaminergic neurons	[485]
	Ferritin	In vitro	Astrocytes increased ferritin release to respond to iron overload, which might inhibit iron-mediated oxidative damage and ferroptosis of dopamine neurons in PD	[486]
	Trx-1	In vivo/In vitro	Trx-1 inhibits ferroptosis in PD through regulating GPX4 and GSH	[487]
ALS	SPY1	In vitro	Regulation of GCH1 and TFR1	[488]
	GPX4	In vivo	Human GPX4 overexpression in SOD1G93A mice significantly delayed disease onset	[489]
	MPO/HOCl	In vivo/In vitro	Increasing the Bax/Bcl-2 ratio and expression of caspase-3 or inhibiting the expressions of GPX4 and NQO1 and thus leading to irreversible lipid peroxidation	[490]
HD	ALOX5	In vivo	ALOX5 as a major factor required for the ACSL4-independent ferroptosis	[491]
General neurodegenerative disease	VAMP7 and syntax in 4	In vitro	Peroxidated lipids and iron are released from neurons requires the exocytic machinery VAMP7 and syntaxin 4	[492]
	PKAN	In vitro	PKAN astrocytes showed lower GPX4 level and were prone to developing a stellate phenotype, thus gaining neurotoxic features	[493]
	Epac1	In vivo/In vitro	cAMP-Epac1 as a plausible therapeutic target to prevent ferroptosis	[494]
	Cofilin1	In vitro	Cofilin1 acts as a redox sensor in oxidative cell death pathways of ferroptosis, and promotes glutamate excitotoxicity	[495]
	HO-1	In vivo	Reduction of iron deposits in the brain	[496]
	WDR45	In vivo	Mutant WDR45 Leads to Altered Ferritinophagy and Ferroptosis in β-Propeller Protein-Associated Neurodegeneration	[497]

*Abbreviations*: *AD* Alzheimer's disease, *PD* Parkinson's disease, *HD* Huntington's disease, *ALS* Amyotrophic lateral sclerosis, *VAMP7* Vesicle Associated Membrane Protein 7, *PKAN* Neurodegeneration associated with defective pantothenate kinase-2, *GPX4* Glutathione Peroxidase 4, *cAMP* Cyclic adenosine monophosphate, *Epac1* Exchange Protein Directly Activated by cAMP 1, *HO-1* Heme oxygenase-1, *WDR45* WD Repeat Domain 45, *PS1* Presenilin-1, *ALDH2* Aldehyde dehydrogenase 2, *ACSL4* Acyl-CoA Synthetase Long Chain Family Member 4, *PI3K* Phosphoinositide 3-kinases, *AKT* Protein kinase B, *NOX4* NADPH Oxidase 4, *GSK-3β* Alpha synuclein, *PUFA* Polyunsaturated fatty acids, *SEC24B* SEC24 Homolog B, *LRRK2* Leucine-rich repeat kinase 2, *SNX5* Ferroportin 1, *PPARδ* Peroxisome proliferator–activated receptor δ, *Nurr1* The nuclear receptor 4A2, *FPN1* Ferroportin-1, *6-OHDA* 6-hydroxydopamine, *CD74* Cluster of Differentiation 74, *Trx-1* Thioredoxin-1, *GSH* Glutathione, *TFR1* Transferrin receptor 1, *SPY1* Sprouty RTK Signaling Antagonist 1, *SOD1* Superoxide dismutase 1, *Bax* Apoptosis regulator BAX, *Bcl-2* B-cell leukemia-2, *NQO1* NAD(P)H dehydrogenase, *ALOX5* Arachidonate 5-lipoxygenase

**Table 6:** Updated compounds targeting ferroptosis in neurodegenerative disease

Diseases	Compounds	Models	Function	References
AD	Senegenin	In vitro	Increased ACSL4 and PEBP1 proteins, and decreased GPX4	[498]
	Eriodictyol	In vivo/In vitro	Eriodictyol inhibits ferroptosis via vitamin D receptor mediated Nrf2 activation	[499]
	Forsythoside A	In vivo	Anti-ferroptosis and anti-neuroinflammatory effects in erastin-stimulated HT22 cells, and the Nrf2/GPX4 axis played a key role in these effects	[500]
	Hydroxylated chalcones	In vitro	Inhibit ferroptosis induced by RSL or erastin and reduce the lipid peroxidation levels induced by Aβ1-42 protein aggregation	[501]
	Salidroside	In vivo	Inhibiting ferroptosis via activation of the Nrf2/GPX4 axis	[502]
	Tetrahydroxy stilbene glycoside	In vivo	Enhanced NLRP3, and also the expression of DMT1, ACSL4 and NCOA4, were reduced by TSG administration	[503]
PD	Quercetin	In vivo/In vitro	Activating the Nrf2 protein	[504]
	Clausenamide	In vivo	Clau directly interacted with the Ser663 of ALOX5, the PKCα-phosphorylation site, and thus prevented the nuclear translocation of ALOX5, which was essential for catalyzing the production of toxic lipids 5-HETE	[505]
	Hinokitiol	In vivo/In vitro	Activating cytoprotective transcription factor Nrf2 to upregulate the antioxidant genes	[506]
	iPLA2β	In vivo/In vitro	Phospholipase iPLA2β averts ferroptosis by eliminating a redox lipid death signal	[507]
	Novel 2-(4-(benzyloxy)-5-(hydroxyl) phenyl) benzothiazole	In vitro	Derivatives as multifunctional MAO-B inhibitors for the treatment of Parkinson's disease	[508]
	Novel flavone 1,2,4-oxadiazole	In vivo/In vitro	Conducted by their inhibitory activities against ROS	[509]
	Thonningianin A	In vivo	Activating the Keap1-Nrf2	[510]
General Neurodegenerative disease	Cannabinol	In vitro	Activate the antioxidant defense system via the upregulation of Nrf2, HO-1, SOD2 and GPX4	[511]
	Caveolin-1	In vivo/In vitro	The overexpression of cav-1 may attenuate DACD by modulating neuronal ferroptosis-mediated mitochondrial homeostasis	[512]
	Selenium Compounds	In vitro	Selenium plays an essential role in reducing lipid peroxidation generated during ferroptosis through its incorporation into the catalytic site of GPX4	[439]
	Liproxstatin-1	In vivo	Liproxstatin-1 decreased the activation of microglia and the release of IL6 and tumor TNFα, attenuated oxidative stress and lipid peroxidation	[513]

*Abbreviations*: *cav-1* Caveolin 1, *DACD* Low-Molecular-Mass Penicillin Binding Protein 6b, *IL6* Interleukin 6, *TNFα* Tumor necrosis factor, *PEBP1* Phosphatidylethanolamine binding protein 1, *Nrf2* Nuclear factor erythroid 2–related factor 2, *HO-1* Heme oxygenase-1, *SOD2* Superoxide dismutase 2, *ACSL4* Acyl-CoA Synthetase Long Chain Family Member 4, *PEBP1* Phosphatidylethanolamine binding protein 1, *GPX4* Glutathione Peroxidase 4, *Aβ* Amyloid beta, *NLRP3* NLR Family Pyrin Domain Containing 3, *DMT1* Natural resistance-associated macrophage protein 1, *NCOA4* Nuclear receptor coactivator 4, *TSG* Tumor necrosis factor-α-stimulated gene/protein, *ALXO5* Arachidonate 5-lipoxygenase, *5-HETE* 5-Hydroxyeicosatetraenoic acid, *MAO-B* Monoamine oxidase B, *KEAP1* Kelch-like ECH-associated protein 1

#### Alzheimer’s disease

AD manifests as a catastrophic neurodegenerative disorder typified by the incremental loss of cognitive faculties, memory deterioration, and behavioral metamorphoses. It represents the predominant form of dementia, impacting millions globally [514]. Despite exhaustive research efforts, the precise mechanisms piloting AD pathogenesis remain enigmatic. Recently, ferroptosis has surfaced as a prospective mechanism with implications for the genesis and advancement of AD. Recent evidence posits that cerebral iron correlates with hastened cognitive decline in individuals exhibiting Alzheimer's pathology [515]. Scott and colleagues have delineated variations in cerebrospinal fluid ferritin levels [470]. Furthermore, the aggregation of amyloid-beta (Aβ) plaques, which constitute the signature pathologies of AD, have been associated with ferroptosis-linked mechanisms [516]. Aβ accumulation may incite oxidative stress and lipid peroxidation, heightening neuronal susceptibility to ferroptosis. Deteriorated antioxidant defenses and diminished activity of crucial enzymes engaged in lipid peroxide detoxification have been witnessed in AD, further corroborating the participation of ferroptosis in neuronal expiration [502, 517]. Deciphering the role of ferroptosis in AD might yield novel insights into the disease trajectory and prospective therapeutic interventions. Interfering with ferroptosis pathways might represent a propitious strategy for attenuating neurodegeneration and cognitive degradation in AD. Diverse pharmacological methodologies, including iron chelators, antioxidants, and ferroptosis inhibitors, have demonstrated promise in preclinical explorations by diminishing neurotoxicity and enhancing cognitive function in AD animal prototypes [476, 499, 500, 503, 518]. However, numerous challenges and unresolved queries persist. Augmented research is imperative to illuminate the precise molecular mechanisms underpinning ferroptosis in AD and its contribution to the progressive neurodegeneration witnessed in afflicted individuals. Additionally, fine-tuning therapeutic interventions targeting ferroptosis, including the development of selective and efficacious drugs, determination of an appropriate treatment window, and managing potential off-target ramifications, is crucial for successful clinical translation.

#### Parkinson’s disease

PD represents a chronic, relentlessly progressive neurodegenerative disorder distinguished by the degradation of dopaminergic neurons within the substantia nigra territory of the brain. This neuronal death culminates in the characteristic motor symptoms of PD, encompassing tremors, rigidity, and bradykinesia. Evolving evidence proposes that ferroptosis may constitute a critical determinant in the pathogenesis of Parkinson's disease. In postmortem cerebral evaluations from individuals afflicted with PD, an elevation of iron regulatory protein 1 (IRP1) activity was discerned within the substantia nigra (SN). This amplified activity could provoke a diminution in ferritin concentrations and an intensification in neuronal iron assimilation, culminating in escalated TfR1 expression. Consequently, the melanized neurons within the SN become increasingly vulnerable to oxidative damage affiliated with iron [519]. Augmented DMT1 concentrations, in conjunction with diminished Cp ferroxidase activity, have been documented in both PD patients and animal representations of PD. These manifestations are posited to contribute to the noticeable amplification in iron levels [520]. Alpha-synuclein (α-Syn), abundantly expressed within the brain and implicated in numerous pivotal synaptic processes of neurons, can bind to Fe^2+^ or Fe^3+^ to fabricate the α-Syn-iron complex. The up-regulation of DMT1 ensuing from α-Syn overexpression also exerts a profound influence on the enhancement of iron uptake and the dysfunction of iron metabolism evidenced in PD [521]. Furthermore, the distorted expression and functionality of proteins involved in iron homeostasis have been detected in PD, further substantiating the association between iron dysregulation and the disease [504, 509, 510, 522, 523].

#### Amyotrophic lateral sclerosis

ALS epitomizes a relentlessly progressive neurodegenerative disorder typified by the selective compromise of cortical and spinal motor neurons, instigating paralysis and ultimately, mortality [524]. Although comprehension of the underlying pathophysiological mechanisms of ALS remains incomplete, the accretion and amassment of ubiquitinated proteinaceous inclusions within motor neurons are broadly recognized as the quintessential neuropathological characteristic of this disease [525]. The majority of ALS instances, roughly 90%, materialize sporadically and fail to exhibit a clear correlation with familial lineage. The residual 10% of cases are tethered to familial inheritance patterns and are typically associated with autosomal dominant mutations. The most prevalently observed mutations transpire within genes such as superoxide dismutase 1 gen (SOD1), TAR DNA-binding protein 43 (TDP-43), FUS, and C9orf72. In a murine model of GPX4 neuronal inducible knockout, the specific depletion of GPX4 within neurons precipitated rapid paralysis, severe muscular atrophy, and ultimately, mortality, thereby evincing symptoms evocative of ALS [526]. A recent study unveiled the depletion of GPX4 in postmortem spinal cord samples from both sporadic and familial ALS patients, revealing a potential involvement of GPX4 in the pathogenesis of ALS [489]. Moreau et al. demonstrated that the administration of deferiprone to ALS patients engendered a significant reduction in iron concentration within the cervical spinal cord [527]. However, the potential influence of ferroptosis inhibition on enhancing the quality of life and survival rate among ALS patients remains undetermined and demands further inquiry.

#### Huntington’s disease

HD manifests as an inheritable neurodegenerative disorder typified by the gradual degeneration of specific neuronal populations within the brain. It is initiated by a mutation in the huntingtin gene (HTT), culminating in the synthesis of an aberrant form of the huntingtin protein [528]. HD is characterized by an extensive array of motor, cognitive, and psychiatric symptoms that progressively intensify over time. Song et al. unveiled that ALOX5-mediated ferroptosis serves as a distinct cell death trajectory in response to oxidative stress in Huntington's disease [491]. Klepac et al. identified a significant diminution (28%) in plasma GSH concentrations among individuals afflicted with HD compared to age and sex-congruent controls [529]. Magnetic resonance imaging revealed an accumulation of iron within the cerebral regions of HD patients [530]. Nevertheless, the pathway inciting ferroptosis within the brain remains largely ambiguous. The potential to procure similar outcomes through alternative strategies for ferroptosis inhibition, such as the modulation of GPX4, lipid peroxidation, and iron-storage proteins, has yet to be explored. Moreover, the question of whether ferroptosis inhibition can effectively decelerate the progression of HD remains unaddressed and necessitates further exploration in prospective investigations.

### Ferroptosis and cardiovascular diseases

Cardiovascular diseases (CVDs) encompass a wide range of conditions affecting the heart and blood vessels, contributing to acute illnesses that result in numerous fatalities worldwide [531]. The death of fully differentiated cardiomyocytes plays a crucial role in the development of various cardiovascular conditions. In this study, we provide a comprehensive perspective on the molecular mechanisms underlying ferroptosis in the pathogenesis of several cardiovascular diseases, including hypertension, atherosclerosis, myocardial infarction (MI), pulmonary hypertension (PH), cardiomyopathy, and heart failure (HF) [39, 532].

When examining cardiomyopathy, our investigation focused on several subtypes, namely Diabetic cardiomyopathy (DCM), Hypertrophic cardiomyopathy, post-transplant cardiomyopathy, Septic cardiomyopathy, Doxorubicin-induced cardiomyopathy (DIC), and radioactive cardiomyopathy [533–537]. The primary objective was to explore the association between the diverse spectrum of CVDs and ferroptosis, while also identifying potential novel compounds that target iron metabolism and ferroptosis within the context of CVDs (Tables 7 & 8).

**Table 7:** Updated therapeutic targets of ferroptosis in cardiovascular diseases

Diseases	Therapeutic targets	Models	Potential mechanisms	References
MI	ME2	In vivo/ In vitro	Inhibit miR-214-3p	[538]
	Adaptor protein HIP-55	In vivo/ In vitro	Regulate AKT/MAPK pathways	[539]
	FNDC5/irisin	In vitro	Regulate Nrf2/HO-1 axis	[540]
	LncRNA Gm47283	In vitro	Target miR-706 / Ptgs2	[541]
DCM	RDH10	In vivo	Mediated disorder of cardiac retinol metabolism	[542]
	TRIM46	In vitro	Regulate GPX4	[543]
	PA	In vivo/In vitro	Regulate HSF1 and GPX4	[544]
	NRF2	In vivo	Regulate AMPK/NRF2 pathways	[545]
	CD74	In vivo/ In vitro	Regulate NLRP3/pyroptosis-mediated regulation of ferroptosis	[546]
DIC	DR-Ab	In vivo/In vitro	Maintain the stability of SLC7A11 on the cell surface	[547]
	PRMT4	In vivo/In vitro	Inhibit Nrf2/GPX4 pathway	[548]
	FUNDC2	In vivo/ In vitro	Regulate GSH and stability of GPX4	[549]
	SIRT1	In vivo/ In vitro	Attenuate oxidative damage	[550]
	p53/Park7	In vivo/In vitro	Regulate p53, restore Fe-S clusters and maintain iron homeostasis	[551, 552]
	Exosomal thioredoxin-1	In vivo	decrease MDA, iron content and increase GSH level	[553]
	METRNL	In vivo/In vitro	activate SIRT1 via cAMP/PKA signaling axis improve DOX-elicited oxidative stress, apoptosis and cardiac dysfunction	[554]
	ADAR2	In vivo/In vitro	regulate miR-34a in CMs, affect pro-proliferation and anti-apoptosis effects	[555]
	p62-NRF2/HO-1	In vivo/In vitro	Reduce iron levels and lipid peroxidation	[556]
Heart failure	FUNDC1	In vivo/ In vitro	Inhibit lipid peroxidation	[557]
	MiR-375-3p	In vivo/ In vitro	Target GPX4-an inhibitor of the ferroptosis pathway	[345]
Sepsis-induced cardiomyopathy	ICA69	In vivo/ In vitro	Induce STING Induce intracellular lipid peroxidation	[558]
	TMEM43	In vivo/ In vitro	Regulate P53-SLCA11 pathway	[559]
Spsis-induced myocardial injury	N6-methyladenosine writer METTL3	In vitro	Regulate SLC7A1 mRNA with high methylation level	[560]
DEHP-induced myocardial injury	Heme-oxygenase-1	In vivo	Activate Nrf2/HO-1 pathway	[561]
Myocardial cell injury induced by heat stroke	TLR4	In vitro	Inhibition of TLR4 alleviates Inflammation and Ferroptosis	[562]
Hypertrophic cardiomyopathy	SLC7A11	In vivo	Overexpression of Slc7a11 increases cellular glutathione levels	[563]
Mitochondrial cardiomyopathy	Oma1	In vivo/In vitro	Regulate GPX4	[564]
Monocrotaline-induced pulmonary hypertension	PRDX6	In vivo/In vitro	Regulate HMGB1/TLR4/NLRP3 signalling	[565]

*Abbreviations*: *MI* Myocardial infarction, *DCM* Diabetic cardiomyopathy, *RDH10* Retinol dehydrogenase 10, *DIC* Doxorubicin-induced cardiomyopathy, *ME2* Malic enzyme; 2, *FNDC5* Fibronectin type III domain-containing protein 5, *RDH10* Retinol dehydrogenase 10, *TRIM46* Tripartite Motif Containing 46, *HSF1* Heat shock factor 1, *GPX4* Glutathione peroxidase 4, *NRF2* Nuclear factor erythroid2-related factor 2, *CD74* MIF membrane receptor cluster of differentiation 74, *NKA* Na^+^/K^+^ ATPase, *PRMT4* Protein arginine methyltransferase 4, *GSH* Glutathione, *FUNDC2* FUN14 domain–containing 2, *SIRT1* Sirtuin 1, *METRNL* Meteorin-like protein, *FUNDC1* FUN14 domain containing 1, *ICA69* Islet cell autoantigen 69, *TMEM43* Transmembrane protein 43, *TLR4* Toll-like receptor 4, *SLC7A11* Solute Carrier Family 7 Member 11, *Oma1* Overlapping with the m-AAA protease 1 homolog, *PRDX6* Recombinant Peroxiredoxin 6, *ICA69* Islet cell autoantigen 69

**Table 8:** Updated compounds targeting ferroptosis in cardiovascular diseases

Diseases	Compounds	Models	Function	References
MI	miR-26b-5p	In vivo	Induce SLC7A11 expression	[566]
	Idebenone	In vivo/In vitro	Regulate ROS-AMPK-mTOR pathway	[567]
DCM	Curcumin	In vivo/In vitro	Regulate Nrf2, increase the expression of oxidative scavenging factors	[568]
	Isorhapontigenin	In vivo	Regulate PRDX2-MFN2-ACSL4 pathway	[569]
	Sulforaphane	In vivo	Regulate AMPK/NRF2 pathways	[545]
DIC	Steviol	In vivo/In vitro	Unknown	[570]
	Ethoxyquin	In vivo/In vitro	Antioxidant	[571]
	Histamine/H1R axis	In vivo/In vitro	Regulate STAT3-SLC7A11 pathway	[572]
	AsIV	In vivo	Activate Nrf2 signaling pathway and promote GPX4 expression	[573]
	5-ALA	In vivo/In vitro	Inhibits iron overload	[574]
	Biomimetic Nanozymes	In vivo/In vitro	Induce GPX4	[575]
	LCZ696	In vivo/In vitro	Regulate AKT,SIRT2/SOD696 pathway	[576]
	Liquiritin	In vivo/In vitro	Regulate SLC7A11, GPX4	[577]
	LAP	In vitro	Regulate PI3K/AKT pathway	[578]
	Ergothioneine	In vivo	Clear ROS, reduce pro-inflammatory mediators, chelate Fe, and maintain mitochondrial function	[579]
	Salidroside	In vivo	Activate AMPK-dependent signaling pathways, regulate fatty acid metabolism and maintain mitochondrial function	[580]
	Epigallocatechin-3-gallate	In vivo	Reduce iron accumulation, inhibit oxidative stress and abnormal lipid metabolism	[581, 582]
	5-ALA	In vivo/In vitro	Maintain heme synthesis, inhibit iron overload and lipid peroxidation	[574]
	Salidroside	In vivo/In vitro	Regulate fatty acid metabolism, maintain mitochondrial function, and downregulate ferrocyte death	[580]
	PAESe	In vivo/In vitro	Prevent a decrease in FXN levels, resist oxidation, increase glutathione levels, and inhibit respiratory decay	[583]
	Fisetin	In vivo/In vitro	Regulate the SIRT1/Nrf2 signaling pathway, increasing GPX4 levels, Reducing MDA and lipid ROS levels, increasing glutathione (GSH), and antioxidant activity	[533]
Heart failure	Berberine hydrochloride	In vivo/In vitro	Inhibit Nrf2-dependent ferroptosis	[584]
Heart failure, DCM	Canagliflozin	In vivo	Activate AMPK/SIRT1/PGC-1a pathway	[585, 586]
Adriamycin cardiomyopathy, Post-transplant, cardiomyopathy, Atherosclerosis, Septic cardiomyopathy, DCM, Palmitic acid cardiac injury	Ferrostatin-1	In vivo	Inhibit lipid peroxidation	[587]
5-FU-induced cardiotoxicity, DIC	Resveratrol	In vivo/In vitro	Inhibit GPX5 Upregulated the p62-NRF2/HO-1 pathway Mediates the miR-149/HMGB1 axis	[556, 588, 589]
TZM-induced cardiotoxicity	Empagliflozin	In vitro	Unknown	[590]
high-fat diet-induced cardiac injury	Celastrol (Cel)	In vitro	Regulate AKT/GSK3β signaling pathway	[591]
Coronary microembolization	Atorvastatin	In vivo/In vitro	Regulate Hif1a/Ptgs2 pathway	[592]
Sepsis-induced myocardial damage	Puerarin	In vivo	Induce AMPK pathway	[593]
Atherosclerosis	QXJYG	In vivo/In vitro	Regulate GPX4/xCT signaling pathway	[594]
Atrial fibrillation	Icariin	In vivo/In vitro	Regulate SIRT pathway	[595]
High-power microwave-induced cardiomyopathy	Tanshinone IIA	In vivo/In vitro	Promote GPX4, SLC7A11 expression	[537]

*Abbreviations*: *PH* Pulmonary hypertension, *MI* Myocardial Infarction, *GPX4* Glutathione Peroxidase 4, *SLC7A11* Solute Carrier Family 7 Member 11, *NRF2* Nuclear factor erythroid2-related factor 2, *AMPK* AMP-activated protein kinase, *SIRT1* Sirtuin 1, *GSH* Glutathione, *FXN* Iron-sulfur cluster biogenesis protein Frataxin, *ROS* Reactive oxygen species, *HO-1* Heme oxygenase-1, *GSK3β* Glycogen synthase kinase-3, *AKT* Protein kinase

#### Myocardial infarction

MI culminates in cardiac detriment precipitated by cellular death and inadequate self-regeneration of cardiomyocytes [596]. Previous investigation has elucidated that ferroptosis participates in MI, which involves lipoprotein receptor-related protein 6 (LRP6) and circRNA1615 [597]. LPR6 and circRNA1615 function as a modulator of ferroptosis via autophagy regulation [597]. Ferroptosis, in concert with hypoxia, assumes a pivotal role in acute myocardial infarction (AMI), prompting Liu et al. to delineate key genes associated with AMI, ferroptosis, and hypoxia that might serve as novel biomarkers or prospective therapeutic targets for AMI [598]. Gao et al. unveiled that lncRNA Gm47283 orchestrates its effect by targeting miR-706 and Ptgs2, thus modulating Ptgs2 expression and downstream ferroptosis, thereby establishing itself as a primary risk factor for MI [541].

Understanding the intricate and complex interplay between MI and ferroptosis is crucial in identifying potential therapeutic strategies. Targeting the molecular mechanisms involved in ferroptosis, such as iron metabolism, peroxidation, and antioxidant systems, may offer new approaches to mitigate the damage caused by myocardial infarction and improve patient outcomes. As shown in Table 7, Malic enzyme 2 (ME2), Adaptor protein HIP-55, and fibronectin type III domain containing 5 (FNDC5)/irisin have also been suggested as potential targets mediating ferroptosis in MI.

#### Atherosclerosis

Atherosclerosis typifies a chronic inflammatory disease hallmarked by dysregulated lipid metabolism and endothelial malfunction [599, 600]. Vinchi et al. have explicated the interplay between ferroptosis and the pathogenesis of Atherosclerosis [601]. They found that GPX4 mitigates the evolution of atherosclerosis via curtailing lipid peroxidation and diminishing the sensitivity of vascular cells to oxidized lipids [602]. Qing-Xin-Jie-Yu Granule (QXJYG), a traditional Chinese medicinal compound constituted of quintuple Chinese medicinal constituents, could inhibit ferroptosis through the regulation of the GPX4/xCT pathway for atherosclerosis [594]. Currently, investigations into the therapeutic efficacies of Chinese medicine on cardiovascular diseases mediated by ferroptosis are sparse [568, 593]. Thus, elucidating the role and mechanism of Chinese medicine in impeding ferroptosis might shed light on the treatment of cardiovascular diseases.

#### Pulmonary hypertension

PH is a condition characterized by elevated arterial blood pressure in the pulmonary circulation, placing increased strain on the heart and ultimately leading to heart failure [603]. Patients with PH commonly experience progressive shortness of breath, which is the predominant symptom observed. Unfortunately, the prognosis for individuals with pulmonary hypertension is generally poor, as treatment options are limited and the disease significantly impacts their quality of life [604]. The pathogenesis of PH involves multiple complex cellular processes and pathological changes. The pathogenesis of PH involves complex cellular processes and pathological changes. Notably, various types of PH are associated with diverse inflammatory responses. In animal models, several immunomodulatory interventions have demonstrated the ability to modulate the progression and advancement of the disease [605]. These findings highlight the importance of understanding the intricate cellular mechanisms involved in the development of PH and suggest potential avenues for therapeutic interventions.

Disruption of signaling pathways involving ROS and nitric oxide (NO) can contribute to the proliferation of pulmonary arterial endothelial cells (PAECs) and pulmonary artery smooth muscle cells (PASMCs), leading to DNA damage, metabolic dysregulation, and vascular remodeling [606]. Growing evidence supports the role of ferroptosis in the development and progression of PH, highlighting the potential of antioxidant therapy as a significant area of investigation for PH treatment. miRNAs have been found to modulate the process of ferroptosis and regulate the expression of target genes involved in iron metabolism in PH patients. Specifically, six differentially regulated miRNAs (miR-483-5p, miR-27a-3p, miR-27b-3p, miR-26b-5p, miR-199a-5p, and miR-23b-3p) have been implicated in PH, indicating their role in the regulation of iron-related pathways [607]. In a study by Xie et al., it was observed that ferroptosis is upregulated in PAECs from rats with monocrotaline (MCT)-induced PH. The authors proposed that pulmonary endothelial ferroptosis triggers an inflammatory response through the HMGB1/TLR4/NLRP3 inflammasome signaling pathway in rats. Pharmacological inhibition of ferroptosis using Ferrostatin 1 (Fer-1) was found to mitigate the progression of MCT-induced pulmonary vascular remodeling and protect the right ventricle from the effects of PH [608].

Considering these findings, the utilization of ferroptosis inhibitors in PH treatment and the exploration of innovative therapies based on the regulation of iron-dependent cell death hold promise for the management of PH.

#### Cardiomyopathy

##### Diabetic cardiomyopathy

DCM is a common complication of diabetes mellitus (DM) and is associated with an increased risk of heart failure and mortality among diabetic individuals [609]. The disease is characterized by left ventricular hypertrophy and diastolic dysfunction in the early stages, progressing to dominant heart failure with reduced systolic function in advanced stages. The pathogenesis of DCM is multifactorial, primarily involving insulin resistance and hyperglycemia [610].

Insulin resistance, an emblematic feature of type 2 diabetes, instigates compromised glucose uptake and utilization by cardiomyocytes, culminating in energy depletion and perturbed cardiac metabolism [611]. Conversely, hyperglycemia contributes to the genesis of advanced glycation end products (AGEs), oxidative stress, and inflammation, thereby exacerbating cardiac dysfunction and provoking structural remodeling [612]. Despite hyperglycemia governing numerous pathways within DCM, the amplification of ROS is perceived as the central mechanism underlying adverse remodeling [613]. The induction of ferroptosis precipitates an elevation in intracellular levels of lipid ROS, consequently inciting cellular death [614]. Recent evidence increasingly implicates ferroptosis as a significant player in the progression of DCM [545]. Intriguingly, sulforaphane-activated NRF2 can repress ferroptosis in cardiomyocytes via the modulation of SLC7A11 levels, indicating a novel therapeutic strategy for DCM [545].

As our comprehension of the pathophysiological mechanisms underlying DCM and ferroptosis continues to advance, innovative therapeutic approaches targeting ferroptosis pathways may emerge. By directing interventions toward pivotal regulators of ferroptosis, such as iron metabolism and lipid peroxidation, it may be plausible to ameliorate the deleterious effects of ferroptosis and augment cardiac function in individuals afflicted with DCM.

##### Hypertrophic cardiomyopathy

Hypertrophic cardiomyopathy delineates a gradually evolving compensatory mechanism for cardiac functionality, predominantly arising in the context of chronic stress overload [536]. It is denoted by a surge in total myocardium and heightened contractility, thereby maintaining regular blood circulation [615]. Evidence points to ferritin's pivotal role in guarding against cardiac ferroptosis, mediated by SLC7A11. Under a high-iron diet, ferritin-deficient mice demonstrated severe heart damage and hypertrophic cardiomyopathy with a distinctive iron death molecular signature, while SLC7A11 overexpression in these mice forestalled cardiac iron death and remodeling [563]. Wang et al. conducted bioinformatics analysis into the pathogenesis of hypertrophic cardiomyopathy (HCM) and dilated cardiomyopathy (DCM) by focusing on the mechanisms of ferroptosis [616]. Their findings revealed that three hub genes, namely POSTN, IGFBP5, and FMOD, have the potential to serve as valuable biomarkers or therapeutic targets in the field of cardiomyopathies. Nevertheless, the exact characteristics of these gene mechanisms associated with ferroptosis remain largely uncertain, especially when considering their implications in myocardial diseases. There might still be underlying mechanisms awaiting clarification to provide an explanation for this phenomenon. In contrast to DCM, the role of ferroptosis in HCM seems to be more intricate, and the precise impact of ferroptosis on HCM remains undisclosed.

##### Doxorubicin-induced cardiomyopathy

Doxorubicin (DOX), a widely used chemotherapeutic agent for various malignancies, possesses significant cardiac toxicity as its most notable side effect, often leading to cardiomyopathy [617–619]. Consequently, there is considerable potential for the development of therapeutic approaches aimed at addressing or mitigating the cardiac damage caused by this drug. Doxorubicin-induced cardiomyopathy (DIC) arises from a complex interplay of various mechanisms, including DNA damage, oxidative stress, intracellular signaling, transcription factors, epigenetic regulatory factors, autophagy, and metabolic inflammation [620, 621].

Emerging evidence increasingly implicates ferroptosis as a pivotal process in the progression of DIC. Wang et al. demonstrated that miR-21-5p effectively inhibits apoptosis and oxidative stress in primary cardiomyocytes and mouse heart tissue exposed to DOX, offering potential leads for novel treatments in cardiovascular diseases [622]. Notably, Na Ta et al. discovered that the mitochondrial outer membrane protein FUNDC2 governs the occurrence of iron-mediated cell death, shielding cells from this fate in Fundc2-knockout mice and MEF cells. Further investigations revealed that FUNDC2 modulates the stability of mito-GSH, GPX4, and SLC25A11, all of which are crucial in DOX-induced ferroptosis and subsequent cardiomyopathy [549]. Wang et al. unveiled that PRMT4 exerts inhibitory effects on the NRF2/GPX4 signaling pathway, accelerating ferroptosis in DIC. This compelling evidence suggests that targeting PRMT4 could potentially serve as a preventive strategy to DIC [548].

Additionally, various models of cardiomyopathy induced under different conditions were examined, alongside an exploration of the potential influence of pharmacological interventions on ferroptosis in these disease models, as outlined in Table 7. The development of ferroptosis inhibitors, coupled with a deeper understanding of the iron-dependent cell death process, holds the promise of breakthroughs in the treatment strategies for cardiomyopathy. Therefore, researchers can delve into the regulatory mechanisms and signaling pathways associated with ferroptosis to better understand its role and impact in cardiovascular diseases. This line of inquiry will shed light on the relationship between ferroptosis and the development of cardiovascular conditions, offering fresh insights and strategies for early disease diagnosis, prevention, and treatment.

### Ferroptosis and autoimmune diseases

Autoimmune diseases impact approximately 8–9% of the global population, yet the underlying mechanisms remain inadequately explored [623]. However, the study of ferroptosis offers a fresh vantage point for investigating these conditions, introducing a novel perspective into the realm of autoimmune disease research. One of the contributing factors to the development of autoimmunity is the aberrant initiation of cell death and inadequate clearance of deceased cells, leading to the exposure or release of intracellular contents that activate the immune system [624]. Ferroptosis plays a substantial role in influencing both the quantity and functionality of immune cells625. Numerous autoimmune diseases, such as Systemic Lupus Erythematosus (SLE), Rheumatoid Arthritis (RA), Inflammatory Bowel Disease (IBD), and Multiple Sclerosis (MS), are intricately associated with ferroptosis [625]. Although different autoimmune diseases may exhibit shared clinical manifestations, each possesses distinct characteristics. For example, RA patients primarily experience polyarthritis affecting the joints of the hands, while major extra-articular organs, such as the kidneys, are rarely involved. Conversely, individuals with SLE may suffer from organ damage caused by excessive production of multiple autoantibodies and subsequent deposition of immune complexes composed of antibodies and antigens in various organs, including the kidneys [625]. Therefore, investigating the potential mechanisms could offer valuable insights for therapeutic approaches targeting autoimmune diseases[626, 627] (Tables 9 & 10).

**Table 9:** Updated therapeutic targets of ferroptosis in autoimmune diseases

Diseases	Therapeutic targets	Models	Potential mechanisms	References
SLE	CREMα	In vivo	IFN-α or SLE serum suppresses GPX4 expression by enhancing CREMα binding to the Gpx4 promoter	[628]
	CoQ10	In vivo	Suppress lipid peroxidation and ferroptosis	[629]
	HMGB1	In vivo/In vitro	Promote ultraviolet B (UVB)-induced tissue damage; Activate mDCs by up-regulating the mTOR pathway	[630, 631]
RA	TNF-α	In vivo	Macrophages release TNF-α to increase GSH biosynthesis and protect FAPα-positive synovial fibroblasts from ferroptosis	[632–634]
	MMPs	In vivo	ROS activates matrix metalloproteinase (MMPs), inhibit cartilage proteoglycan synthesis, promote FLSs proliferation and chondrocyte apoptosis	[635, 636]
	SAM	In vivo	Glycine enhance ferroptosis via SAM-mediated GPX4 promoter methylation and ferritin decrease	[632, 637]
	G1dP3	In vitro	Promote RASFs ferroptosis cell death via a p53/SLC7A11 axis-dependent mechanism	[638]
	SIRT1	In vivo/In vitro	Transcriptionally repressed by YY1 and inhibits the ferroptosis	[639–642]
IBD	IL-6 and CXCL1	In vivo	Dietary AA induces production of IL-6 and CXCL1, reduces expression and enzymatic activity of GPX4, and caused lipid peroxidation and ferroptosis	[625]
	Nrf2	In vivo	APS prevents ferroptosis by inhibiting the NRF2/HO-1 pathway	[643]
MS	HMOX1, LPCAT3, RPL8	In vitro	Potential ferroptosis targets for interventional strategies in MS	[644]
	Nrf2	In vivo	Inhibitor of LPS-induced inflammation	[645]
IgAN	CD71	In vivo	Receptor for binding to IgA1	[646]
	Hepcidin	In vivo	Defends against iron-mediated renal injury	[647]
EAE	EZH2/SLC7A11	In vivo	BMSC-Exos carrying miR-367–3p inhibit microglial ferroptosis via EZH2/SLC7A11 axis	[648, 649]
	GPX4	In vitro	Inhibit the functions of CD4 T cells^+^	[650]
	ACSL4	In vivo	PUFAs are esterified by ACSL4 and oxidized by iron to generate toxic PE-AA-OOH that destroy cellular membranes during ferroptosis	[15, 261, 651]
AS	DDIT3	In vivo	Change inflammatory response in the immune microenvironment	[652]
	HSPB1	In vivo	Change inflammatory response in the immune microenvironment	[652]
UC	Furin	In vivo	Inhibit epithelial cell injury and alleviates experimental colitis by activating the Nrf2-Gpx4 signaling pathway	[653]
AIH	FGF4	In vivo	Inhibite ferroptosis of hepatocytes by increasing CISD3 levels and activating Nrf2/HO-1 signaling	[654]

*Abbreviations*: *MS* Multiple sclerosis, *EAE* Experimental autoimmune encephalomyelitis, *UC* Ulcerative colitis, *AIH* Autoimmune hepatitis, *SLE* Systemic lupus erythematosus, *CREMα* cAMP-responsive element modulator α, *CoQ10* Coenzyme Q10, *HMGB1* High mobility group box-1 protein, *IFN-α* Human interferon-α, *UVB* Ultraviolet B, *RA* Rheumatoid arthritis, *TNF-α* Tumor necrosis factor-α, *GSH* Glutathione, *FAPα* Fibroblast activation protein α, *MMPS* Matrix metalloproteinase, *ROS* Reactive oxygen species, *SAM* S-adenosylmethionine, *FLSs* Fibroblast-like synoviocytes, *RASFs* RA synovial fibroblasts, *SIRT1* Silent information regulator sirtuin 1, *CXCL1* C-X-C motif chemokine ligand 1, *IECs* Intestinal epithelial cells, *IBD* Inflammatory bowel disease, *IL-6* Interleukin- 6, *Nrf2* Nuclear factor erythroid2-related factor 2, *LPS* Lipopolysaccharides, *HMOX1* Heme oxygenase 1, *RPL8* Ribosomal Protein L8, *TfR1* Transferrin Receptor 1, *IgAN* IgA Nephropathy, *AKI* Acute kidney injury, *EZH2* Enhancer of zeste homolog, *SLC7A11* Solute Carrier Family 7 Member 11, *BMSC* Bone mesenchymal stem cell, *ACSL4* Acyl-CoA Synthetase Long Chain Family Member 4, *PUFAs* Polyunsaturated fatty acids, *AS* Ankylosing spondylitis, *DDIT3* DNA damage-inducible transcript 3, *HSPB1* Heat shock protein family B (small) member 1, *FGF4* Fibroblast growth factor 4

**Table 10:** Updated Compounds targeting ferroptosis in autoimmune diseases

Diseases	Compounds	Models	Function	References
SLE	Liproxstatin-1	In vivo	Suppress lipid ROS levels in neutrophils and significantly attenuate lupus in mice models	[628]
	Idebenone	In vivo	Downregulate NETs formation in neutrophils; Improve mitochondrial metabolism and ATP production; Ameliorate endothelium-dependent vasorelaxation and reduce lipid peroxidation	[629]
	MitoQ	In vivo	Reduce NETs and ROS, downregulate serum levels of IFN and reduce immune complex formation in kidneys	[629]
OA	Ferrostatin-1	In vivo	Rescue the collagen II expression and attenuated the cartilage degradation and OA progression	[655, 656]
IBD	OTSSP167	In vivo	MELK-selective inhibitor, inhibit ferroptosis and reduce DSS-induced colitis in mice by suppressing the protein kinase B (AKT)/IKK/p65 and extracellular signal-regulated kinase (ERK)/IKK/p65 signaling cascades	[657]
	Ti_3_C_2_NSs	In vivo/In vitro	Eliminate excess ROS against oxidative stress-induced cell damage	[658]
EAE	Ferrostatin-1	In vivo	Suppress the expression of MDA and 4-HNE in oligodendrocyte; enhance GPX4, xCT expression	[659]
AIH	Ferrostatin-1	In vivo	Ameliorate the influence of AIH on the Nuclear factor E2-related factor 2 (Nrf2)/Heme oxygenase-1 (HO-1) signaling pathway	[660]
Synovitis	ICA	In vitro	Activate the Xc-/GPX4 axis	[661]
	IKE and etanercept	In vivo	Induce ferroptosis in synovial fibroblasts and attenuate arthritis progression	[633]
Osteoarthritis	pPADN	In vitro	Scavenge ROS	[662]
EAP	DFO/EDA	In vivo	Chelate iron ions and scavenge free radicals	[663]
Lupus nephritis	Liproxstatin-2	In vivo	Inhibit the ferroptosis of human proximal tubular cells	[664]

*Abbreviations*: *Lip-1* Liproxstatin-1, *ROS* Reactive oxygen species, *IDE* Idebenone, *SLE* Systemic lupus erythematosus, *NETs* Neutrophil extracellular traps, *ATP* Adenosine 5'-triphosphate, *MitoQ* Mitochondrial-targeted coenzyme Q10, *IFN* Interferon, *Fer-1* Ferrostatin-1, *RA* Rheumatoid arthritis, *Nrf2* Nuclear factor erythroid2-related factor 2, *IBD* Inflammatory bowel disease, *MELK* Maternal embryonic leucine zipper kinase, *DSS* Dextran sulfate sodium salt, *ERK* Extracellular signal-regulated kinase, *EAE* Experimental autoimmune encephalomyelitis, *AIH* Autoimmune hepatitis, *HO-1* Heme oxygenase-1, *ICA* Icariin, *TfR1* Transferrin receptor 1, *GPX4* Glutathione peroxidase 4, *NCOA4* Nuclear receptor coactivator 4, *IKE* Imidazole ketone erastin, *GCs* Glucocorticoids, *DPEP1* Dipeptidase 1, *SLC7A11* Solute carrier family 7 member 11, Dipeptidase 1, *pPADN* phenylboronic acid modified L-DOPA-derived nanoparticles, *OA* Osteoarthritis, *DFO* Deferoxamine, *EDA* Ethylenediamine, *EAP* Experimental autoimmune prostatitis, *LN* Lupus nephritis, *ASH* Alcohol-associated steatohepatitis, MDMX Murine double minute X, *PPARα* Peroxisome proliferator-activated receptor

#### Systemic lupus erythematosus

SLE is a severe, debilitating autoimmune disease that affects multiple organs and body systems. The prevalence of SLE worldwide is estimated to be as high as 150 per 100,000 individuals [665]. The disease is characterized by autoantibodies against nuclear antigens (ANA), which are caused by a dysregulation of the immune system [666]. Recent investigations have revealed that neutrophils derived from lupus-prone mice or individuals with SLE undergo cell death through the process of ferroptosis [667]. Notably, the presence of autoantibodies and interferon α in the serum acts as a stimulant for neutrophil ferroptosis. This stimulation leads to an increased binding of the transcriptional suppressor CREMα to the GPX4 promoter, resulting in the suppression of GPX4 expression. Consequently, this cascade of events promotes the accumulation of lipid-ROS [165, 166]. In mice, the presence of neutrophil-specific GPX4 haploinsufficiency leads to the development of a phenotype resembling SLE. Additionally, inhibiting ferroptosis in vivo slows down the progression of the disease in lupus-susceptible MRL/lpr mice. These findings shed light on the involvement of neutrophil ferroptosis in the underlying causes of SLE [667]. Additionally, the effective suppression of lipid ROS levels in neutrophils and the significant inhibition of lupus development in a murine model have been observed through the use of the ferroptosis inhibitor liproxstatin-1 [667]. Furthermore, the proliferation of pathogenic T cells, specifically T follicular helper (Tfh) cells, plays a crucial role in the pathogenesis of SLE [668]. Iron overload promotes the expansion of Tfh cells, secretion of pro-inflammatory cytokines, and antibody production in mice prone to lupus. Mice subjected to a high-iron diet exhibited an increased proportion of Tfh cells and antigen-specific germinal center responses [669]. At the molecular level, overexpression of miR-21 inhibits 3-hydroxybutyrate dehydrogenase-2 (BDH2), leading to iron accumulation and enhanced activity of Fe^2+^-dependent TET enzymes. This, in turn, results in hydroxymethylation of the BCL6 gene and differentiation of Tfh cells. In summary, maintaining iron homeostasis is crucial for controlling the proliferation of pathogenic T cells, might provide novel therapeutic potential in treating SLE [669].

#### Rheumatoid arthritis

The primary pathogenesis of RA involves immune dysfunction and inflammation, leading to notable pathological changes such as synovitis, progressive cartilage degradation, and subchondral bone destruction [670]. While the exact mechanism of RA remains unknown, immune cells and fibroblast-like synoviocytes (FLS) are believed to play significant roles in disease progression [671, 672]. For instance, in FLS associated with RA, glycine has been shown to enhance s-adenosylmethionine (SAM) levels, leading to SAM-mediated GPX4 promoter methylation and decreased FTH1 expression. These actions regulate the ferroptosis process [632]. Furthermore, the inhibition of system xc- by Erastin has been demonstrated to induce damage to cartilage tissue by upregulating the expression of matrix metalloproteinase 13 (MMP-13) in chondrocytes and suppressing type II collagen expression, thereby exacerbating RA [655]. umor Necrosis Factor (TNF), a pivotal pro-inflammatory cytokine in the pathogenesis of RA, has been found to inhibit ferroptosis by upregulating SLC7A11, glutamate-cysteine ligase catalytic subunit (GCLM), and glutamate-cysteine ligase regulatory subunit (GCLC). This, in turn, promotes cystine uptake and cellular GSH biosynthesis [633]. In a Collagen-Induced Arthritis (CIA) mouse model, low doses of an undisclosed compound (IKE) along with the TNF antagonist etanercept induced ferroptosis in fibroblasts and attenuated the progression of arthritis [633]. These findings elucidate the mechanisms by which TNF modulates resistance to ferroptosis and suggest the therapeutic potential of ferroptosis-focused therapies targeting dysregulated fibroblasts across a broader range of diseases [633].

#### Inflammatory bowel disease

IBD is a progressive and recurrent condition with a rising global incidence, encompassing both Crohn's Disease (CD) and Ulcerative Colitis (UC) [673]. These diseases are characterized by extensive cell death in the gut and colon due to chronic inflammation [673, 674]. In an experimental colitis model induced by Dextran Sodium Sulfate (DSS), upregulation of HO-1 within the inflamed colon has been observed, leading to anti-inflammatory and antioxidative effects[675]. NF-κB is involved in the production of cytokines and chemokines in inflammatory cells, as well as the regulation of Endoplasmic Reticulum (ER) stress signaling and ferroptosis processes [676–678]. One study suggests that phosphorylated NF-κB-p65 protects intestinal epithelial cells from ferroptosis by alleviating endoplasmic reticulum stress, potentially indicating therapeutic targets for UC treatment involving ferroptosis and NF-κB-p65 phosphorylation [679]. Curculigoside (CUR), the main active constituent of Rhizoma Curculiginis, exhibits diverse biological activities and has shown protective effects on intestinal epithelial cell death, GSH levels, Malondialdehyde (MDA) content, and Lactate Dehydrogenase (LDH) activity. These effects are significantly diminished upon knockdown of GPX4 [680]. CUR prevents ferroptosis in UC by inducing GPX4, highlighting the potential of GPX4 as a therapeutic target for UC [680, 681]. Studies indicate that ferroptosis inhibitors such as Liproxstatin-1 (Lip1), Fer-1, and Deferoxamine (DFO) alleviate disease symptoms and prevent colon length reduction in DSS-induced colitis in mice, emphasizing the beneficial impact of ferroptosis inhibition on IBD [679, 681, 682]. Overall, targeting ferroptosis inhibition may offer a new avenue for the treatment of IBD.

#### Multiple sclerosis

MS is characterized by chronic inflammation in the central nervous system, marked by neuroinflammation, demyelination, oligodendrocyte depletion, and neurodegeneration [683]. Microglia, known for their ability to alter transcriptional profiles and exhibit diverse inflammatory phenotypes, play a crucial role in the development of MS [684]. The ferroptosis inducer RSL3 has been found to reduce inflammation in microglia and peritoneal macrophages (PM) in response to lipopolysaccharide (LPS) stimulation, while conditioned medium from cells undergoing ferroptosis significantly amplifies inflammation in these cells [645]. Interestingly, despite their resistance to ferroptosis, BV2 cells and PMs exhibit reduced inflammation by increasing the abundance of NRF2 protein. Treatment with RSL3 and Fer-1 concurrently decreases systemic inflammation in vivo [645]. However, the precise mechanism of ferroptosis in MS remains to be fully elucidated.

Overall, cytokines, such as Tumor Necrosis Factor-alpha (TNF-α) and Interferon-alpha (IFN-α), modulate ferroptosis in different ways, contributing uniquely to the pathogenesis of autoimmune diseases [628, 633]. Therefore, it is crucial to understand the intricate interactions between different cell death pathways and the significance of these interactions in the context of autoimmune diseases.

### Ferroptosis and infection

Infection embodies a dynamic interaction entailing the complicatedly interplay and conflict between invading pathogens and the host organism [685]. The infection process commences once these pathogens breach the host's defenses via diverse avenues, often culminating in substantial detriment to host cells [686]. Recent research accentuates the cardinal role of ferroptosis in the context of pathogenic infections, as expounded extensively in several recent studies [685, 687, 688]. Consequently, this discourse aims to encapsulate our current understanding of the nexus between ferroptosis and pathogenic infections, emphasizing the underpinning molecular mechanisms, principal regulators, and prospective therapeutic approaches.

A range of pathogens—encompassing bacteria, viruses, fungi, and parasites—typically cause diseases via three mechanisms: direct cellular damage, toxin activity, and immune response [689]. Emerging evidence underscores a robust association between pathogenic infections and ferroptosis [690] (Table 11). On one side of this balance, the host organism can curtail infection by inciting ferroptosis; for instance, oxidative degradation of cellular lipids can suppress hepatitis C virus (HCV) replication [691]. Conversely, certain pathogens may bolster their proliferation and survival by inducing ferroptosis; mycobacterium tuberculosis (Mtb), for example, initiates ferroptosis to augment its pathogenicity and dissemination [692]. These pathogens orchestrate ferroptosis by impeding lipid peroxidation [693]. Comprehending the potential signaling mechanisms of ferroptosis in the context of pathogenic infections will undoubtedly pave the way for the development of novel therapeutic agents (Table 12).

**Table 11:** Updated therapeutic targets of ferroptosis in infections

Diseases	Therapeutic Targets	Models	Potential Mechanisms	References
Sepsis	Sestrin2	In vivo/In vitro	Downregulate the ATF4-CHOP-CHAC1 signaling pathway	[694]
	AUF1	In vivo/In vitro	Upregulate NRF2 expression; downregulate ATF3 expression	[695]
	ADSCs exosomes	In vivo/In vitro	Upregulate GPX4 in PMVECs	[696]
	YAP1	In vivo/In vitro	Disrupted the interaction between NCOA4 and FTH1; prevent the degradation of ferritin to Fe2 + ; inhibit lipid peroxidation	[21]
	MUC1	In vivo/In vitro	Downregulate Keap1 expression; upregulate GPX4 expression	[697]
	eCIRP	In vivo/In vitro	Downregulate GPX4 expression; increase lipid peroxidation	[698]
	NETs	In vivo/In vitro	Downregulate GPX4 expression	[699]
	METTL3	In vivo/In vitro	Downregulate GPX4 expression	[699]
Pulmonary tuberculosis	GPX4	In vivo/In vitro	Inhibit lipid peroxidation	[700]
	Heme oxygenase-1	In vivo/In vitro	Modulate intracellular ROS production	[701]
	Mycobacterium tuberculosis Rv1324 Protein	In vivo/In vitro	Increase lipid peroxidation	[702]
	HIF-1α/SLC7A11/GPx4	In vivo/In vitro	Increase lipid peroxidation; reduce antioxidant levels	[703]
P. aeruginosa infection	15-lipoxygenase	In vivo/In vitro	Upregulate 15-HpETE-PE signaling pathway	[704]
	iNOS/NO•	In vitro	Inhibit lipid peroxidation	[705]
	RNase E	In vitro	Increase pyoverdine and pyochelin siderophore gene expression	[706]
Clostridium difficile infection	aryl-hydrocarbon receptor	In vivo/In vitro	Downregulate CYP1A1 expression; increase lipid peroxidation	[31]
F. nucleatum infection	PEBP1	In vivo/In vitro	Upregulate Raf1-MAPK signaling pathways	[707]
Hepatitis A virus	3Cpro	In vivo	Increase lipid peroxidation	[708]
Hepatitis B virus	miR-222	In vivo/In vitro	Downregulate TFRC expression	[709]
	Hepatitis B virus protein X	In vivo/In vitro	Downregulate SLC7A11 and GPX4 expression	[649]
	SRSF2/PCLAF tv1 axis	In vivo	Upregulate GPX4 expression	[710]
Hepatitis C virus	FADS2	In vivo/In vitro	Increase lipid peroxidation	[691]
Zika virus	HMOX1	In vivo	Upregulate the Nrf2‐SLC7A11‐HO‐1 pathway	[32]
	SAT1	In vivo	Increase lipid peroxidation	[32]
	SLC40A1	In vivo	Export iron	[32]
	CybB	In vivo	Increase lipid peroxidation	[32]
Influenza	mFeS	In vivo/In vitro	Inactivate the extracellular influenza virus by inducing viral ferroptosis depending on Fe^2+^	[711]
	NRF2-KEAP1-GCLC signal pathway	In vivo/In vitro	Induce l-glutamine metabolic reprogramming	[712]
Enterovirus infection	ACSL4	In vitro	Upregulate ACSL4 expression	[713]
E. piscicida infection	c-di-GMP	In vivo/In vitro	Promotes iron accumulation, mitochondrial dysfunction, and production of reactive oxygen species	[714]
Viral encephalitis- HSV-1	Nrf2-Keap1	In vivo/In vitro	Inhibit lipid peroxidation	[715]
Endometritis	Farnesoid X receptor	In vivo/In vitro	Downregulate GXP4 and SLC7A11 expression	[716]
Epstein–Barr virus	PSTK	In vivo/In vitro	Downregulate GXP4 expression	[717]
CVB3 virus	Sp1	In vivo/In vitro	Upregulate TFRC expression	[718]
LCMV	mTORC2	In vivo/In vitro	Upregulate GPX4 expression	[719]
AIDS	CD36	In vivo/In vitro	Increase lipid peroxidation	[720]
	HIV-1 Tat	In vivo/In vitro	Downregulate the expression of miR-204, upregulate the expression of its target- ACSL4	[721]

*Abbreviations*: *15-HpETE-PE* 15-hydroperoxy-arachidonoyl-PE, *iNOS* Inducible nitric oxide synthase, *Keap1* Kelch-like ECH-associated protein 1, *GCLC* Glutamate-cysteine ligase catalytic subunit, *ATF4* Activating transcription factor 4, *CHOP* C/EBP homologous protein, *CHAC1* Glutathione specific gamma- glutamylcyclotransferase 1, *AUF1* AU-rich RNA-binding factor 1, *Atf3* Activating transcription factor 3, *ADSCs* Adipose mesenchymal stem cell, *PMVECs* Pulmonary microvascular endothelial cell, *YAP1* Yes-associated protein, *NCOA4* Nuclear receptor coactivator 4, *FTH1* Ferritin Heavy Chain 1, *MUC1* Mucin 1, *eCIRP* Extracellular cold-inducible RNA-binding protein, *NETs* Neutrophil extracellular traps, *METTL3* m6A enzyme methyltransferase-like 3, *CYP1A1* Cytochrome P450 1A1, *c-di-GMP* cyclic dimeric GMP, *ROS* Reactive oxygen species, *HIF-1α* Hypoxia-inducible factor-1alpha, *SLC7A11* Solute carrier family 7 member 11, *HMOX1* Heme oxygenase 1, *SAT1* Spermidine/spermine N (1)-acetyltransferase-1, *SLC40A1* Solute carrier family 40 member 1, *CybB* Cytochrome b, *3Cpro* 3C protease, *miR-222* microRNA-222, *TFRC* Transferrin receptor, *SRSF2* Serine/arginine-rich splicing factor 2, *PCLAF* Proliferating cell nuclear antigen clamp-associated factor, *tv1* variant 1, *FADS2* Fatty acid binding protein 2, *AIDS* Acquired immunodeficiency syndrome, *CD36* Cluster of differentiation 36, *HSV-1* Herpes simplex virus 1, *PSTK* O-phosphoseryl-tRNA (Sec) kinase, *Sp1* Specificity protein 1, *TFRC* Transferrin receptor 1, *LCMV* Lymphocytic choriomeningitis virus, *mTORC2* mechanistic target of rapamycin complex 2, *PEBP1* Phosphatidylethanolamine binding protein 1, *MAPK* Mitogen-activated protein kinase

**Table 12:** Updated compounds targeting ferroptosis in infections

Diseases	Compounds	Models	Function	References
Sepsis	HET0016	In vivo/In vitro	Inhibit STING pathways; upregulate GPX4 and FTH expression	[722]
	Uridine	In vitro	Upregulate Nrf2/HO-1 axis	[723]
	Proanthocyanidins	In vivo/In vitro	Inhibit lipid peroxidation	[724]
	Itaconate	In vivo/In vitro	Upregulate Nrf2/HO-1 axis; upregulate GSH and GPX expression	[725]
	Vitamin E	In vivo/In vitro	Inhibit lipid peroxidation	[726]
Pulmonary tuberculosis	Zinc oxide nanoparticles	In vivo/In vitro	Inhibit lipid peroxidation	[727]
P. aeruginosa infection: skin infection	FeCl_3_	In vivo/In vitro	Increase intracellular labile Fe^2+^; inhibit lipid peroxidation	[728]
P. aeruginosa infection: Intestinal infection	Baicalein	In vivo/In vitro	Upregulate GPX4 expression	[704]
P. aeruginosa infection: pulmonary infections	Idebenone	In vivo/In vitro	Inhibit lipid peroxidation	[729]
P. aeruginosa infection: pulmonary infections	Gallium nitrate	In vitro	Target and inhibit siderophores	[706]
F. nucleatum: periodontitis	Piperlongumine	In vivo/In vitro	Reduce the level of intracellular Fe^2+^, ameliorate the impairment in mitochondrial function	[707]
	Fisetin	In vivo/In vitro	Reduce the level of intracellular Fe^2+^, ameliorate the impairment in mitochondrial function	[707]
COVID-19	Deferoxamine	In vivo/In vitro	Chelate iron; downregulate hepcidin I expression	[730]
	Lactoferrin	In vivo/In vitro	Bind iron and inhibit viral replication	[731]
	Vitamin C	In vitro	Inhibit lipid peroxidation	[732]
	Melatonin	In vitro	Chelate iron and block iron-dependent lipid peroxidation	[732]
	Vitamin E	In vitro	Inhibit lipid peroxidation	[732]
Hepatitis C virus	IKE	In vivo/In vitro	Inhibit system xc^−^, increase lipid peroxidation	[691]
	BWA4C	In vivo/In vitro	Increase lipid peroxidation; inhibit viral replication	[691]
Enterovirus infection	Rosiglitazone	In vitro	Downregulate ACSL4; inhibit viral replication	[713]
	Pioglitazone	In vitro	Downregulate ACSL4; inhibit viral replication	[713]
Endometritis	Obeticholic acid	In vivo/In vitro	Upregulate FXR expression	[716]
Malaria	Deferoxamine	In vivo/In vitro	Chelate iron; Inhibit lipid peroxidation	[733]
	Liproxstatin-1	In vivo/In vitro	Inhibit lipid peroxidation	[733]
	Artemisinin	In vivo/In vitro	Increase lipid peroxidation	[733]

*Abbreviations*: *GPX4* Glutathione Peroxidase 4, *STING* Stimulator of interferon response cGAMP interactor 1, *Nrf2* Nuclear factor erythroid 2–related factor 2, *HO-1* Heme oxygenase-1, *GSH* Glutathione, *GPX* Glutathione peroxidase, *ACSL4* Acyl-CoA Synthetase Long Chain Family Member 4, *IKE* Imidazole ketone erastin, *FXR* Farnesoid X Receptor, *Lip-1* Liproxstatin-1

#### Bacterial infection

Bacteria can provoke host tissue deterioration and organ impairment through the activation of ferroptosis [42]. For instance, Pseudomonas aeruginosa (P. aeruginosa), an important opportunistic pathogen, is the main cause of ventilator-associated pneumonia, urinary tract, blood flow and chronic infection [734–736]. However, P. aeruginosa show natural resistance to many classes of antibiotics [737]. Not only that, the effectiveness of antimicrobials in treating P. aeruginosa infection has gradually declined in recent years [738, 739]. Hopefully, regulate ferroptosis to intervene in the development of various resistance mechanisms in P. aeruginosa has emerged as a promising treatment option [740]. P. aeruginosa possesses the capability to express lipoxygenase (pLoxA), which catalyzes the oxidation of host AA-phosphatidylethanolamine (AA-PE) to 15-hydroperoxy-AA-PE (15-Ho-AA-PE), thereby instigating ferroptosis in human bronchial epithelial cells [741]. Concurrently, it undermines the host’s GPX4 defenses by mobilizing lysosomal chaperon-mediated autophagy (CMA)^14^. In response, the host organism activates the inducible nitric oxide synthase/nitric oxide (iNOS/NO•) driven anti-ferroptosis mechanism to halt lipid peroxidation [705]. Consequently, pLoxA and iNOS/NO• may serve as potential therapeutic targets for P. aeruginosa-associated afflictions, such as cystic fibrosis. Another investigation demonstrated that ferric chloride could relieve P. aeruginosa-mediated cell death [728]. Baicalein, a mammalian lipoxygenases inhibitor, markedly relieves animal mortality, PAO1 colonization, intestinal epithelial cell death, and generation of ferroptotic oxidized phosphatidylethanolamine (PEox) signals [704]. These studies underscore that P. aeruginosa propagation occurs through ferroptosis, thereby motivating us to explore strategies to curb P. aeruginosa infection by focusing on ferroptosis-induced necrosis.

Ferroptosis is also critical to the pathogenic mechanism of Mtb, which is the main pathogenic factor of tuberculosis [693]. Tuberculosis is one of the world's deadliest infections and, along with malaria and HIV/AIDS, has the most significant socio-economic impact on humanity [742]. It is reported that ferroptosis plays a major role in cell death and tissue necrosis induced by Mtb [55]. Protein tyrosine phosphatase A (PtpA), an effector secreted by Mtb, inhibits GPX4 expression by targeting protein arginine methyltransferase 6 (PRMT6), ultimately precipitating ferroptosis and bolstering the pathogenicity and dispersion of Mtb [692]. In an in vivo context, GPX4-deficient mice infected with Mtb displayed a significant upsurge in lung necrosis and bacterial load, meanwhile, an outcome relieved by the lipid peroxidation inhibitor, ferrostatin-1 [700]. These findings support the role of iron-induced death in Mtb-triggered necrosis. Further research revealed that the knockdown of heme oxygenase 1 (Hmox1) by siRNA resulted in a diminution of antioxidant factors GPX4 and FSP1 [32], along with an increased release of intracellular bacteria in Bacillus Calmette-Guérin (BCG)-infected macrophages [743]. These observations suggest that Mtb propagation occurs through ferroptosis, thereby inspiring us to search for promising strategies to manage pulmonary tuberculosis by focusing on ferroptosis-induced necrosis.

Ferroptosis also has a bearing on the evolution and progression of sepsis [744]. Sepsis is a severe medical condition characterized by dysfunctional organ function resulting from the host's inadequate response to infection [745]. The immune response, initiated by the invading pathogen, fails to restore normal balance, leading to a pathological syndrome characterized by sustained inflammation and immunosuppression [746]. Intense stress during sepsis can disrupt the metabolic processes of ions, lipids, and energy in organisms [747]. Numerous studies have increasingly demonstrated the significant role of ferroptosis in modulating inflammation and sepsis [748, 749]. The interplay between Stimulator of Interferon Response cGAMP Interactor 1 (STING) and Nuclear Receptor Coactivator 4 (NCOA4) triggers ferritin-phagocytosis mediated ferroptosis, culminating in an amplified inflammatory response and impacting the transcription factor [722]. Subsequent studies revealed that HET0016, a selective 20-HETE synthase inhibitor, could reverse this mechanism [722]. Moreover, our body can also alleviate sepsis by modulating ferroptosis. Sestrin 2 (Sesn2), a stress-responsive protein, inhibits ferroptosis in septic Dendritic Cells (DC) by downregulating the ATF4-CHOP-CHAC1 signaling pathway [694]. Yes-associated protein 1 (YAP1), a crucial regulator of the Hippo signaling pathway, can disrupt the interaction between NCOA4 and ferritin heavy chain 1 (FTH1) to inhibit lipid peroxidation and ferroptosis [21]. Another investigation found that adipose-derived stem cells (ADSCs) exosomes augment the expression of NRF2 and GPX4, resulting in a relief of oxidative stress injury and ferroptosis in lung tissue [696]. These studies collectively suggest that interference with ferroptosis can to some extent govern the progression of sepsis. However, further relevant research in this domain is still required to provide promising target insights and effective therapeutic agents for sepsis.

#### Viral infection

Viral hepatitis, a collection of infectious diseases primarily evidenced by liver inflammation and necrotic lesions, results from the influence of an array of hepatitis viruses [750]. Several different viruses cause hepatitis, including hepatitis A, B, C, D, and E. The hepatitis A and E viruses typically cause acute infections. The hepatitis B, C, and D viruses can cause acute and chronic infections [751]. The transmission of viral hepatitis poses a public health concern, and chronic infection can negatively impact a person's quality of life, leading to symptoms and long-term complications [752]. Studies have shown that ferroptosis may be involved in the development of inflammatory responses, hepatocyte damage, and liver fibrosis in the liver tissues of patients with viral hepatitis [753]. The human hepatitis A virus 3C protease (3Cpro) has been recently identified as the instigator of caspase-independent cell death, with affected cells demonstrating plasma membrane rupture, depletion of mitochondrial potential, and mitochondrial and nuclear engorgement [708]. Subsequent investigations unveiled that cell death orchestrated by 3Cpro was proficiently obviated by ferroptosis inhibitors [708]. These findings infer that 3Cpro expression provokes ferroptosis in human cells. HBV X protein (HBx), a crucial HBV regulatory protein, bears associations with oxidative stress and lipid peroxidation [754]. In vitro and in vivo examinations revealed that HBx curbed the expression of solute carrier family 3 member 2 (SLC3A2), amplifying liver toxicity and ferroptosis induced by d-galactosamine/lipopolysaccharide (D-GalN) [649]. Nevertheless, the host also possesses the capacity to curtail infection via ferroptosis induction. Oxidative degradation of cellular lipids drastically impedes hepatitis C virus (HCV) replication [691]. Yamane and colleagues posited that fatty acid desaturase 2 (FADS2) operates as a rate-limiting factor for ferroptosis, with the escalated expression of FADS2 inhibiting HCV replication^9^. Moreover, BWA4C, a 5-lipoxygenase inhibitor, can endorse LPO restriction to limit HCV replication [691]. Despite the multitude of studies forging connections between viral hepatitis and ferroptosis, additional research is mandated to cultivate pharmaceuticals for these associated targets.

The COVID-19 pandemic in 2019 jolted the global populace. The observation that augmented ferroptosis transpires in various tissues and cells impacted by COVID-19 warrants significant attention. Han and his team discovered that SARS-CoV-2 infection initiates dysfunction in the human sinoatrial node (SAN)-like pacemaker cells and induces ferroptosis [755]. Vitamin K diminishes the levels of ROS by managing the expression of antioxidant enzymes, proven to curtail lipid peroxidation and inhibit ferroptosis, contributing to its therapeutic efficacy in COVID-19 patients [756]. Han and his group, conducting drug screening utilizing Hesc-SAN-like pacemaker cells, recognized imatinib and deferoxamine as potential candidates for safeguarding pacemaker cells against SARS-CoV-2 infection and ferroptosis [756].

One more instance pertains to AIDS. AIDS, caused by the human immunodeficiency virus (HIV), is a chronic infectious disease that primarily attacks the immune system, specifically CD4^+^ T cells [757]. As the virus replicates and weakens the immune system, it significantly compromises the body's ability to defend against infections and diseases [758]. In the context of HIV, it has been observed that key markers of ferroptosis, such as iron accumulation and lipid peroxidation, play a significant role [759, 760]. In further research, Xiao and colleagues documented that classical indicators of ferroptosis were discernible in CD4^+^ T cells of HIV immune non-responders, inclusive of increased lipid peroxidation in mitochondria and destruction of mitochondrial structure [720]. Furthermore, Kannan and colleagues declared that the HIV-1 Tat protein can upregulate ACSL4 expression, escalating lipid peroxidation, which results in the discharge of proinflammatory cytokines and the activation of microglia730. Further studies disclosed that miR-204 functions as an upstream regulator of ACSL4 and inhibits both HIV-1 TAT-mediated ferroptosis and pro-inflammatory cytokine release [721]. These results suggest that the HIV-1 Tat protein and miR-204 might represent potential therapeutic targets against HIV infection. Consequently, the regulation of ferroptosis emerges as a promising therapeutic target and strategy for combating HIV. Nevertheless, further research is necessary to elucidate this and provide treatment strategies targeting ferroptosis for AIDS patients.

#### Other infection

In recent years, there has been growing evidence implicating the involvement of ferroptosis in the pathogenesis of malaria [761], a persistent public health challenge in economically disadvantaged regions, posing a grave threat to the well-being and lives of local populations [762]. Heather S Kain discerned that impeding GPX4 or SLC7A11 precipitates a substantial reduction in malaria liver-stage parasite infection [733]. Further, Erastin and Sorafenib, inhibitors of SLC7A11, exhibit inhibitory actions on malaria [733]. Another study has shown that desferriamine and lipstatin-1 can stimulate cellular lipid peroxidation and the accumulation of unstable iron associated with dead iron [763]. However, the role of ferroptosis in malaria remains murky. Further research is required to elucidate the intricate interactions between factors related to iron toxicity, malarial parasites, and host immune response in order to combat this devastating infectious disease.

Regardless, extant studies of ferroptosis-pathogen interactions remain relatively rudimentary. Moreover, the understanding of the mechanism underlying the pathogenic regulation of ferroptosis is still deficient in certain areas. Particular pathogen infections necessitate more rigorous investigation to provide novel therapeutic strategies for the evolution of antiviral pharmaceuticals or vaccines. A question that arises is how current medications ameliorate disease symptoms via iron-induced death. For instance, the ferroptosis inhibitor, ferrostatin-1, demonstrates a more pronounced inhibitory effect on Mtb-induced ferroptosis than the reactivation of GPX4 [700], intimating those other mechanisms may participate in Mtb-induced ferroptosis. Recognizing which cellular constituents are involved in the regulation of pathogen-associated ferroptosis may likewise lay the groundwork for drug screening initiatives designed to treat infectious diseases.

### Ferroptosis in iron-overload diseases

Iron-overload diseases represent a cluster of disorders distinguished by the excessive accumulation of iron within the body. Several circumstances such as genetic aberrations, blood transfusions, or extended intake of iron supplements can initiate an iron load that outstrips the capacity of iron-binding proteins, precipitating tissue damage [764, 765]. Common iron-overload maladies encompass hereditary hemochromatosis, alcoholic liver disease (ALD), chronic liver disease, and aplastic anemia [766–768]. Left untreated, these diseases can cause serious health problems, including liver disease, heart disease, diabetes, and arthritis [767–769]

Iron-overload diseases can be divided into two categories: primary and secondary overload [770]. The primary iteration, exemplified by hereditary hemochromatosis and juvenile haemochromatosis, predominantly arises from genetic perturbations, which attenuate hepcidin levels and enhance bodily iron absorption, instigating excessive iron deposition within internal organs [767, 770–773]. Conversely, the pathogenesis of secondary iron overload predominantly originates from ineffective hematopoiesis, induced by auto-anemic disorders, leading to diminished secretion levels of hepcidin and an upsurge in intestinal iron absorption, thereby engendering iron overload [774, 775]. Concurrently, human blood transfusions liberate substantial quantities of iron, culminating in an iron overburden [774].

#### Chronic liver disease

The liver, due to its inherent predisposition to oxidative detriment, frequently presents with excessive iron accretion, a quintessential characteristic pervading a plethora of severe hepatic afflictions [776]. Studies have delineated iron overload as a seminal feature of ALD [777, 778], postulating that ethanol may engender liver iron overburden through assorted mechanisms. These encompass the activation of iron-regulatory proteins, thereby elevating transferrin receptor expression, and the repression of the transcription factor CCAAT/enhancer-binding protein α(C/EBPα) or bone morphogenetic protein (BMP)-mediated Smad signaling pathways, resulting in attenuated hepcidin expression [779–781].

This hepcidin downregulation fosters increased expression of the divalent metal transporter 1 and ferroportin within the duodenum, culminating in enhanced intestinal iron absorption [782, 783]. Hence, strategies aiming to augment hepcidin or activate the transferrin receptor may exhibit therapeutic potential [20, 778, 784]. Utilization of specific antioxidants, such as vitamin E and N-acetylcysteine (NAC), may relieve alcohol-mediated C/EBPα inhibition in the liver, reduce hepcidin expression, and enhance DMT1. Simultaneously, targeting hepatic sirtuin 1 and cytochrome P450 2E1(CYP2E1) may also provide therapeutic benefits in ALD, principally through lipid peroxidation reduction [20, 785, 786].

Non-alcoholic fatty liver disease and steatohepatitis, conversely, are characterized by iron-deficient hepatocytes and iron overload in hepatic stellate cells [787]. This phenomenon occurs due to iron deficiency intensifying hepatocyte lipogenesis and insulin resistance through HIF2α-ATF4 signaling, while the accumulation of iron engenders excess reactive oxygen species production, thereby exacerbating liver fibrosis [787–789]. Iron chelators such as DFO can efficaciously reverse abnormal lipid metabolism and hepatic damage induced by high-fat, high-iron diets [790–792]. Therapeutic approaches that aim to enhance NRF2 activity have been shown to facilitate ubiquitination and proteasomal degradation of target proteins, which are mediated by Kelch-like ECH-associated protein 1, β-transducin repeat-containing protein, and/or HMG-CoA reductase degradation protein 1 [793]. Preventative measures such as Vitamin C, quercetin, mitochondrial ROS scavenger Mito-TEMPO, and curcumol to thwart steatosis, and the utilization of iron chelators or ferroptosis inhibitor liproxstatin-1 to maintain iron homeostasis, are favorable approaches to relieve nonalcoholic fatty liver disease (NAFLD) [794–797].

In conclusion, iron overload-induced chronic liver disease caused by iron overload is a complex condition that requires multidisciplinary approaches for effective management. While current therapies aim to reduce iron burden and relieve the harmful effects of oxidative stress and inflammation, emerging therapies targeting specific pathways involved in disease pathogenesis offer hope for improved treatment outcomes in the future.

#### Brain iron accumulation

Cerebral iron accumulation, a prevalent comorbidity in a multitude of cognitive and motor function disorders such as AD, PD, multiple system atrophy, and multiple sclerosis, is often evidenced by heightened iron deposition in the brain [469, 503, 798–803]. Yet, the mechanistic comprehension of the correlation between this accumulation and neurodegenerative disorders remains insufficient [802].

Friedrich's ataxia (FRDA), a monogenic recessive neurodegenerative condition, is characterized by progressive cerebellar and sensory ataxia, precipitated by the amplification of GAA repeats within the frataxin (FXN) gene, which encodes for the mitochondrial protein frataxin involved in iron-sulfur cluster biogenesis [804–806]. Frataxin deficiency can influence iron-sulfur cluster-containing proteins, culminating in iron accumulation within the brains and hearts of afflicted individuals [807]. The primary drivers of FRDA encompass aberrant iron metabolism, mitochondrial dysfunction, and subsequent oxidative damage [808].

Frataxin deficiency will curtail the availability of coenzyme A for TfR1 palmitoylation, while compounds such as artesunate, coenzyme A, and dichloroacetate may ameliorate iron overload through the enhancement of TfR1 palmitoylation [804]. In FRDA, NRF2 is typically downregulated, however, treating afflicted fibroblasts with NRF2 inducers like EPI-743 and sulforaphane could rectify iron deficiency and redox imbalance by targeting NRF2-mediated iron homeostasis [809, 810]. Concurrently, the utilization of leriglitazone and targeting of peroxisome-proliferator-activated receptor gamma may serve as efficacious strategies to improve mitochondrial function, thus offering a therapeutic avenue for FRDA [808, 811].

At present, an absolute remedy for FRDA remains elusive, with extant treatment modalities merely offering symptomatic relief. However, recent years have seen the advent of innovative therapeutic stratagems encompassing gene therapy, small molecule pharmaceuticals, and cell-based interpositions, all of which imbue optimism for the development of more efficacious treatment alternatives for afflicted patients.

#### IO-associated endocrine diseases

Accumulating evidence implicates iron dysregulation as a pivotal factor in the progression of an array of endocrine disorders, including those of the pancreas and kidneys [812, 813]. Within the pancreas, iron deficiency in β cells can result in diminished insulin secretion [813, 814]. Similarly, iron deficiency in the liver, adipose tissue, and muscles can induce insulin resistance, thereby mediating the onset and advancement of type 2 diabetes mellitus (T2DM) [769, 813].

Research has revealed that iron overload inhibits insulin secretion and compromises islet β cell function through the downregulation of synaptotagmin 7 (SYT7), both in vivo and in vitro models [815]. This suggests SYT7might present a potential therapeutic target for T2DM. Furthermore, free fatty acids, hyperglycemia, and inflammatory cytokines are principal mediators of β-cell toxicity inT2DM, impairing mitochondrial metabolism [816]. Preservation of mitochondrial homeostasis through glutaredoxin 5, caveolin-1, and mitochondrial electron transport can relieve the impacts of T2DM [512, 816, 817].

In summation, iron overload-induced T2DM embodies a multifaceted condition encompassing an array of mechanisms, inclusive of oxidative stress, inflammation, and mitochondrial dysfunction. Existing therapeutic options remain limited, necessitating innovative therapeutic strategies such as antioxidant therapy and targeted interventions. To develop more efficacious treatments, an intricate understanding of the underlying pathophysiological mechanisms remains paramount. At present, phlebotomy, iron modulator supplementation, and iron chelation therapy stand as the primary modalities to mitigate excessive iron accumulation. Clarifying the root cause of iron overload could indeed enhance clinical therapeutics. Notably, iron overload within the body can precipitate a wide array of organ complications, with numerous specific mechanisms still awaiting elucidation. The future trajectory of iron overload disease management may well lie in employing targeted pharmaceuticals and synthesizing drug complexes from these specific materials. Potential therapeutic targets and compounds are duly summarized in Tables 13 & 14.

**Table 13:** Updated therapeutic targets of ferroptosis in iron-overload/ other diseases

Diseases	Therapeutic targets	Models	Potential mechanisms	References
ALD	FNDC3B	In vivo/In vitro	Induce AMPK;inhibit lipid peroxidation	[818]
	RAGE	In vivo/In vitro	Reduce steatosis, inflammation and oxidative stress of the liver, increase iron uptake (Tf/TfR) and storage (Ferritin), and reduce iron output (FPN1/Hepcidin), affecting the iron metabolism mechanism of hepatocytes and macrophages	[778]
	Hepcidin	In vivo	Downregulation of DMT1 and FPN expression, reducing iron absorption	[782, 790]
	SIRT1	In vivo	NAD( +)-dependent protein deacetylase, Improved iron metabolism, increased liver glutathione content, and reduced lipid peroxidation	[20]
	Vitamin E and N-acetylcysteine	In vivo/In vitro	Antioxidants, abolish the alcohol-mediated down-regulation of C/EBPα binding activity and hepcidin expression and up-regulate DMT1	[779]
	CYP2E1	In vivo/In vitro	Reduce oxidative stress and acute liver injury	[785]
	FXR	In vivo/In vitro	Regulate iron homeostasis, inhibit hepatotoxicity	[819]
	Epo	In vitro	Generate red blood cells, maintain iron homeostasis and metabolism;	[820]
	*Rnf217*	In vivo/In vitro	The target of Tet1, mediates the ubiquitination and subsequent degradation of FPN	[783]
NAFLD/NASH	HIF2α-ATF4	In vitro	Block hepatocyte EV secretion or deplete EV iron cargo, restore liver iron homeostasis	[788]
	PCBP1	In vivo/In vitro	Bind cytosolic iron and deliver it to iron enzymes for activation and to ferritin for storage	[821]
	iGPX4	In vivo/In vitro	Interact with cGPX4 to facilitate the transformation of cGPX4, thus promotes ferroptosis	[512]
	Nrf2	In vivo/In vitro	Regulate glutathione homeostasis, mitochondrial function, lipid metabolism	[793, 809, 822]
FRDA	TfR1	In vivo/In vitro	Affects ISC-related proteins, delays Tf cycle, and affects iron homeostasis	[804]
	FXN	In vivo	Reduce oxidative stress, increase ISC activity, maintain calcium homeostasis, and mitochondrial biological function	[807, 810, 823–826]
	PPARγ	In vivo	Maintain mitochondrial function, inhibit lipid peroxidation	[808]
	Nrf2	In vivo/In vitro	Regulate glutathione homeostasis, mitochondrial function, lipid metabolism	[793, 809, 822]
T2DM	Epo	In vitro	Generate red blood cells, maintain iron homeostasis and metabolism;	[820, 827]
	SYT7	In vivo/In vitro	Increase insulin, enhance β Cell function and glucose tolerance	[815]
	MitoNEET	In vitro	Maintain energy metabolism, iron homeostasis, and free radical production	[817]
	NAF-1	In vitro	Maintain insulin secretion, mitochondrial and ER structure and function	[814]
	Caveolin-1	In vivo/In vitro	Modulate Neuronal Ferroptosis-Mediated Mitochondrial Homeostasis	[512]
	Glrx5	In vivo/In vitro	Involved in the assembly of iron-sulfur clusters required for complexes of the respiratory chain	[816]
OA	GPX4	In vivo/In vitro	Regulate ferroptosis and ECM degradation	[828, 829]
	TRPV1	In vivo/In vitro	Promote the expression of GPX4	[828]
	FGF23	In vivo/In vitro	A critical phosphate-regulating hormone, response to low oxygen/iron	[830]
	NCOA4	In vivo/In vitro	Interact with ferritin, increase autophagic degradation of ferritin and iron levels via JNK-JUN-NCOA4 axis	[831]
β-thalassemia	Tfr2	In vivo/In vitro	Balance red blood cell production, activate ferritin, and regulate erythropoietin signaling	[832, 833]
	TfR1	In vivo/In vitro	Affects ISC-related proteins, delays Tf cycle, and affects iron homeostasis	[834]
DN	ACSL4	In vivo	Reduce lipid peroxidation product MDA and iron content	[835]
CKD	FGF23	In vivo/In vitro	A critical phosphate-regulating hormone, response to low oxygen/iron	[830]
Inflammatory arthritis	Tfr2	In vivo/In vitro	Balance red blood cell production, activate ferritin, and regulate erythropoietin signaling	[832, 833]

*Abbreviations*: *ALD* Alcoholic liver disease, *FNDC3B* Fibronectin type III domain-containing protein 3B, *AMPK* Adenosine 5'-monophosphate-activated protein kinase, *RAGE* Receptor for advanced glycation end products, *Tf* Transferrin, *Tf* Transferrin, *TfR* Transferrin receptor, *FPN1* Ferroportin 1, *DMT1* Divalent metal transporter 1, *SIRT1* Aberrant liver sirtuin 1, *NAD* Nicotinamide Adenine Dinucleotide, *C/EBPα* CCAAT-enhancer-binding protein α, *CYP2E1* Cytochrome P450 2E1, *FXR* Farnesoid X receptor, *Epo* Erythropoietin, *TET1* Tet-methylcytosine dioxygenase 1, *NAFLD* Nonalcoholic fatty liver disease, *NASH* Non-alcoholic steatohepatitis, *HIF-2α* Hypoxia-inducible factor-2alpha, *ATF4* Activating transcription factor 4, *PCBP1* Poly rC binding protein 1, *GPX4* Glutathione peroxidase 4, *cGPX4* Canonical-GPX4, *iGPX4* Inducible-GPX4, *Nrf2* Nuclear factor erythroid 2–related factor 2, *FRDA* Friedreich ataxia, *TfR1* Membrane transferrin receptor 1, *ISC* Iron sulfur cluster, *FXN* Frataxin, *PPARγ* Peroxisome proliferator-activated receptor gamma, *T2DM* Type 2 diabetes mellitus, *SYT7* Synaptotagmin 7, *MitoNEET/NAF-1* Human NEET proteins, *Glrx5* Glutaredoxin 5, *OA* Osteoarthritis, *ECM* Extensive extracellular matrix, *TRPV1* Transient receptor potential vanilloid 1, *FGF23* Fibroblast growth factor 23, NCOA4 Nuclear receptor coactivator 4, *JNK* Jun N-terminal kinase, *Tfr2* Transferrin receptor 2, *DN* Diabetic nephropathy, *ACSL4* Acyl-CoA synthetase long-chain family member 4, *MDA* Malondialdehyde, *CKD* Chronic kidney disease

**Table 14:** Updated compounds targeting ferroptosis in iron-overload/ other diseases

Compounds	Diseases	Models	Function	References
Hepcidin peptide	ALD	In vivo	Increase expression of ferritin	[782]
4-Methylpyrazole		In vivo	Alcohol metabolism enzyme inhibitors, inhibit C/EBPα Reduce ferritin transcription	[779]
Vitamin E and N-acetylcysteine		In vivo	Antioxidants, reduce oxidative stress, increase ferritin expression	[779, 821]
GW4064		In vivo	FXR agonist, Regulate iron homeostasis, inhibit hepatotoxicity	[819]
EWCDs	NAFLD	In vivo/In vitro	Iron chelator, inhibit the production of induced oxygen, reduce endoplasmic reticulum stress, and regulate NF-κB	[791]
Curcumol		In vivo/In vitro	Inhibit hepatocyte senescence through YAP/NCOA4, regulate ferritinophagy	[794]
Vitamin C		In vivo/In vitro	Inhibit PA/OA, induce steatosis and maintains iron homeostasis	[795]
Mito-TEMPO		In vivo/In vitro	Reduce MtROS-mediated ferroptosis, alleviate lipid droplet accumulation and lipid peroxidation	[796]
LPT1		In vivo	Regulate PANoptosis, prevent steatosis	[797]
DFP		In vivo	Iron chelator	[797]
Artesunate, CoA, and dichloroacetate	FRDA	In vivo/In vitro	Improve TfR1 palmitoylation, decrease iron overload	[804]
EPI-743 and SFN		In vivo/In vitro	Induce Nrf2	[809]
Au(8) -pXs		In vivo	Improve mitochondrial reactive oxygen species response	[811]
MIN-102 (INN: leriglitazone)		In vivo	Increase frataxin, improve mitochondrial function and calcium homeostasis, inhibit lipid peroxidation	[808]
SNH6		In vivo	Supplement NAD ( +) and chelate iron	[836]
DFX	T2DM	In vivo/In vitro	Iron chelator, inhibit iron-induced ferroptosis, driving lipid peroxidation	[837]
Naringenin	OA	In vivo/In vitro	Reduce oxidative stress through the NRF2-HO-1 pathway, alleviate cartilage damage under iron overload	[838]
Mitapivat	β-thalassemic	In vivo	A pyruvate kinase activator, Improve the burden of blood transfusion and reduce iron overload	[839]
DFO, efonidipine, DFX FCT		In vivo/In vitro	Iron chelator, inhibit iron-induced ferroptosis, driving lipid peroxidation	[840–842]
DFO	HD, PVL, OA, NAFLD, DoIC	In vivo/In vitro	Iron chelator	[798, 819, 829, 843–845]
Fer-1, deferiprone		In vivo/In vitro	Inhibit cell death; Inhibiti lipid peroxidation, Increase GPX4/GSH levels	[556, 796, 812, 814, 829, 846–848]
Rosi	DN	In vivo	Reduce lipid peroxidation product MDA and iron content	[835]
Esomeprazole	Hereditary anemias, liver iron-overload	In vivo	Proton pump inhibitor, reduce liver iron content	[849]
DFAs	Hemochromatosis, high iron diet-induced/dextran-stimulated iron accumulation	In vivo	Iron chelator, inhibit iron-induced ferroptosis, driving lipid peroxidation	[845]
Metal-curcumin complexes	FRDA, cancer, arthritis, osteoporosis, and neurological disorders such as AD	In vivo/In vitro	Remove iron, reduce oxidative stress, enhance Fe-S clusters, compensate for FXN deficiency, improve the morphology and function of mitochondria	[810, 850]
Empagliflozin	T2DM, anaemia, chronic kidney disease	In vivo	Empagliflozin; Increase red blood cell production and increase early iron utilization	[851, 852]
Momelotinib	MF	In vivo	Regulate BMP6/ACVR1/SMAD and IL-6/JAK/STAT3 pathways, decrease hepcidin (master iron regulator) expression, higher serum iron and hemoglobin levels, and restore erythropoiesis	[853]
HUCMSCs	DMED	In vivo	Upregulate SLC7A11/GPX4, reduce oxidative stress levels, and reduce iron content	[789]
Pkd1	ADPKD	In vivo/In vitro	Increase 4HNE, promote the proliferation of survived Pkd1 mutant cells via activation of Akt, S6, Stat3	[812]
CPX, CPX-O	PKD	In vivo/In vitro	Chelat iron, inhibit iron-dependent enzymes, induce ferritin degradation via ferritinophagy	[854]
Rosi	DN	In vivo	Reduce lipid peroxidation product MDA and iron content	[835]
BCA	IOKOA	In vivo/In vitro	RegulatE iron levels and NRF2/System xc^−^/GPX4 axis, scavenge free radicals and prevent lipid peroxidation, regulate iron homeostasis	[855]
Polydatin	Gouty arthritis	In vivo/In vitro	Regulate PPAR-γ and ferritin activation	[856]

*Abbreviations*: *GW4064* the FXR agonist, *ALD* Alcoholic liver disease, *C/EBPα* CCAAT-enhancer-binding protein α, *FXR* Farnesoid X receptor, *EWCDs* Fluorescent egg white-based carbon dots, *NAFLD* Nonalcoholic fatty liver disease, *NF-Κb* Nuclear factor kappaB, *YAP* Yes-associated protein, *NCOA4* Nuclear receptor coactivator 4, *PA/OA* Palmitic acid (PA)/oleic acid (OA), *Mito-TEMPO* Mitochondrial ROS scavenger, *LPT1* Ferroptosis inhibitor liproxstatin-1, *DFP* Iron chelator deferiprone, *SFN* Sulforaphane, *CoA* Coenzyme A (CoA), *Nrf2* Nuclear factor erythroid 2–related factor 2, *TfR1* Membrane transferrin receptor 1, *Au8-pXs* ROS detoxifying gold quantum clusters, *FRDA* Friedreich ataxia, *SNH6* 6-methoxy-2-salicylaldehyde nicotinoyl hydrazone, *NAD* Nicotinamide Adenine Dinucleotide, *DFX* Deferoxamine, *T2DM* Type 2 diabetes mellitus, *OA* Osteoarthritis, *HO-1* Heme oxygenase-1, *DFO* Deferiprone, *Fer-1* Ferrostatin-1, *HD* Huntington's disease, *PVL* Periventricular leukomalacia, *DoIC* Doxorubicin DOX-induced cardiotoxicity, *GSH* Glutathione, *DN* Diabetic nephropathy, *MDA* Malondialdehyde, *DFAs* new deferric amine compounds, *FXN* Frataxin, *BMP6* Bone morphogenetic protein 6, *ACVR1* Activin A receptor type I, *SMAD* Suppressor of Mother against Decapentaplegic, *IL-6* Interleukin-6, *STAT3* Signal Transducer And Activator Of Transcription 3, *MF* Myelofibrosis, *HUCMSCs* Human umbilical cord mesenchymal stem cells, *SLC7A11* Glutamate-cystine-exchanger Xct, *DMED* Diabetic mellitus erectile dysfunction, *Pkd1* Gene encoding polycystin-1, *ADPKD* Autosomal dominant polycystic kidney disease, *4HNE* 4-hydroxynonenal, *AKT* Akermanite, *S6* Phosphorylated ribosomal S6 protein, *CPX* Ciclopirox, *CPX-O* Ciclopirox’s olamine salt, *PKD* Polycystic kidney disease, *Rosi* ACSL4 inhibitor rosiglitazone, *BCA* Biochanin, *IOKOA* Iron overload-induced KOA, *PAESe* Phenylaminoethyl selenides, *PPAR-γ* Peroxisome proliferator activated receptor gamma

## Conclusion and perspective

Ferroptosis, a form of regulated cell death characterized by iron-dependent accumulation of lipid hydroperoxides, has emerged as a significant area of study in cell biology and disease research. It is distinct from other forms of cell death such as apoptosis, necrosis, and autophagy, and is tightly linked to numerous biological processes, including amino acid, iron, and polyunsaturated fatty acid metabolism, and the biosynthesis of glutathione, phospholipids, NADPH, and CoQ10 [9, 857].

The role of ferroptosis in pathological cell death associated with degenerative diseases, carcinogenesis, stroke, intracerebral hemorrhage, traumatic brain injury, ischemia–reperfusion injury, and kidney degeneration is increasingly being recognized [10, 858–861]. Moreover, the potential of ferroptosis as a tumor-suppressor function that could be harnessed for cancer therapy is an exciting development.

However, the strategies for tumor suppression and organ injury are fundamentally incongruous. Additional elucidation of the mechanisms of iron-dependent cellular death at every disease stage can equip us with more precise preventative and therapeutic strategies. In addition, other forms of cellular death, such as cuproptosis, have been discovered. The roles that these various forms of cell death play in disease processes warrant further exploration.

Therefore, Future investigations in the field of ferroptosis should focus on further elucidating the molecular mechanisms underlying this form of cell death. This includes understanding the roles of key regulators such as GPX4, FSP1, NRF2, NADPH oxidase, and p53 in ferroptosis.

Hitherto, an array of compounds targeting essential proteins have been deployed to either promote or inhibit ferroptosis, though little has been found in clinical application. Hence, the development of effective therapeutic strategies to modulate ferroptosis could have significant implications for the treatment of a wide range of diseases, including cancer and neurodegenerative disorders. The potential of ferroptosis inhibitors in protecting against pathological conditions such as acute kidney injury also warrants further exploration. Whether multi-target therapy will also seize a prominent position in this field remains a topic of ongoing research.

Moreover, the identification of ferroptosis markers is crucial in differentiating them from cell death induced by oxidative stress and in guiding the development and evaluation of ferroptosis-specific drugs. Assuring safety, efficacy, minimizing off-target effects, and ensuring effective drug delivery present formidable challenges to ongoing research.

In general, a deeper understanding of the specific mechanisms of ferroptosis in different diseases and interventions targeting ferroptosis at various stages of disease progression will provide valuable insights and inform more accurate prevention and treatment strategies for patients.

## Data Availability

Not applicable.
